# Prosapogenin A induces GSDME-dependent pyroptosis of anaplastic thyroid cancer through vacuolar ATPase activation-mediated lysosomal over-acidification

**DOI:** 10.1038/s41419-024-06985-z

**Published:** 2024-08-13

**Authors:** Yunye Liu, Yawen Guo, Qian Zeng, Yiqun Hu, Ru He, Wenli Ma, Chenhong Qian, Tebo Hua, Fahuan Song, Yefeng Cai, Lei Zhu, Xinxin Ren, Jiajie Xu, Chuanming Zheng, Lingling Ding, Jingyan Ge, Wenzhen Wang, Haifeng Xu, Minghua Ge, Guowan Zheng

**Affiliations:** 1 Otolaryngology & Head and Neck Center, Cancer Center, Department of Head and Neck Surgery, Zhejiang Provincial People’s Hospital (Affiliated People’s Hospital), Hangzhou Medical College, Hangzhou, Zhejiang China; 2 Zhejiang Key Laboratory of Precision Medicine Research on Head & Neck Cancer, Hangzhou, China; 3 Zhejiang Provincial Clinical Research Center for malignant tumor, Hangzhou, Zhejiang China; 4 Key Laboratory of Bioorganic Synthesis of Zhejiang Province, College of Biotechnology and Bioengineering, Zhejiang University of Technology, Hangzhou, China; 5 Bengbu Medical College, Bengbu, Anhui China; 6 The Second Affiliated Hospital of Jiaxing University, Jiaxing, China; 7 Department of Thyroid Surgery, Ningbo Medical Center Lihuili Hospital, Ningbo, Zhejiang China; 8 Department of Thyroid Surgery, The First Affiliated Hospital of Wenzhou Medical University, Wenzhou, Zhejiang China; 9 Department of Thyroid Surgery, The Fifth Hospital Affiliated to Wenzhou Medical University, Lishui Central Hospital, Lishui City, Zhejiang Province China; 10 Department of Clinical Laboratory, The Children’s Hospital, Zhejiang University School of Medicine, National Clinical Research Center for Child Health, Hangzhou, China

**Keywords:** Cancer, Cell death

## Abstract

Anaplastic thyroid cancer (ATC) is among the most aggressive and metastatic malignancies, often resulting in fatal outcomes due to the lack of effective treatments. Prosapogenin A (PA), a bioactive compound prevalent in traditional Chinese herbs, has shown potential as an antineoplastic agent against various human tumors. However, its effects on ATC and the underlying mechanism remain unclear. Here, we demonstrate that PA exhibits significant anti-ATC activity both in vitro and in vivo by inducing GSDME-dependent pyroptosis in ATC cells. Mechanistically, PA promotes lysosomal membrane permeabilization (LMP), leading to the release of cathepsins that activate caspase 8/3 to cleave GSDME. Remarkably, PA significantly upregulates three key functional subunits of V-ATPase—ATP6V1A, ATP6V1B2, and ATP6V0C—resulting in lysosomal over-acidification. This over-acidification exacerbates LMP and subsequent lysosomal damage. Neutralization of lysosomal lumen acidification or inhibition/knockdown of these V-ATPase subunits attenuates PA-induced lysosomal damage, pyroptosis and growth inhibition of ATC cells, highlighting the critical role for lysosomal acidification and LMP in PA’s anticancer effects. In summary, our findings uncover a novel link between PA and lysosomal damage-dependent pyroptosis in cancer cells. PA may act as a V-ATPase agonist targeting lysosomal acidification, presenting a new potential therapeutic option for ATC treatment.

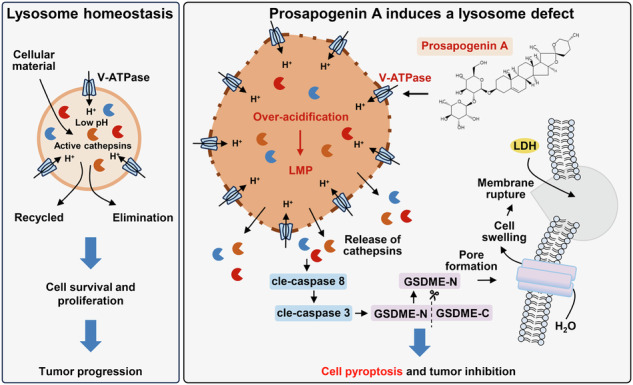

## Introduction

Anaplastic thyroid cancer (ATC) is a rare and highly malignant form of thyroid cancer characterized by its undifferentiated state and extremely poor prognosis [1]. Patients often succumb to the disease due to rapid distal metastasis and a rapidly enlarging neck mass causing compressive symptoms, with disease-specific mortality rates nearing 100% [2]. Despite the availability of various therapies—such as surgery, radiotherapy, chemotherapy, targeted therapy, and immunotherapy—none have been proven effective, resulting in a median survival time of only 3 to 7 months and a 1-year survival rate of merely 20% [3, 4]. Even the FDA-approved combination of dabrafenib and trametinib has shown only marginal survival benefits, and only in ATC cases with the BRAF V600E mutation [5]. Consequently, there is an urgent need to explore novel therapeutic strategies to provide more effective treatment options for patients with ATC.

Lysosomes play a crucial role in the survival and proliferation of both normal and tumor cells by acting as regulatory hub for cellular metabolism, protein degradation, and nutrient sensing [6]. Malignant tumor cells often exhibit alterations in lysosomal characteristics—such as quantity, hypertrophy, hydrolase activity, and pH homeostasis—facilitating the degradation of macromolecules to supply nutrients that promote tumor cell proliferation and survival. Additionally, these lysosomes secrete hydrolytic enzymes that digest the extracellular matrix, aiding tumor cell metastasis [7, 8]. However, these alterations also render tumor lysosomes more fragile, increasing the tendency for lysosomal membrane permeabilization (LMP) and presenting a potential therapeutic target for cancer treatment [9]. Research has shown that lysosomal inhibitors and agents inducing LMP can effectively trigger cancer cell death [10]. Broad-spectrum lysosomal inhibitors, such as vacuolar type ATPase (v-ATPase) inhibitors—including bafilomycin A1 (BafA1), concanamycin, and archazolid A [11]—and autophagy inhibitors, such as the antimalarials and their derivatives (chloroquine, CQ; hydroxychloroquine, HCQ), have been used to treat or improve treatments for various tumors [12, 13]. These inhibitors promote lysosomal deacidification, enhancing LMP induction by antineoplastic drugs and ultimately leading to cell death. Prolonged lysosomal deacidification in cancer cells causes the release of lysosomal enzymes (cathepsins) into the cytoplasm, triggering cell death. Conversely, lysosome reacidification restores lysosomal functions and rescues cells [14, 15]. Intriguingly, both our present study and previous research [16] have observed that lysosomal over-acidification can be lethal. However, it remains largely unknown whether lysosomal over-acidification can provoke cell death of ATC cells and serve as a potential anticancer strategy. Exploring this possibility could uncover new avenues for the treatment of ATC.

Traditional Chinese medicine is a treasure trove of natural small molecules with diverse biological activities, many of which have shown significant anticancer properties against various malignancies. Prosapogenin A (PA) is a typical natural steroid saponin derived from *Veratrum nigrum L*. [17], a renowned traditional Chinese herb traditionally used to treat conditions such as hypertension, epilepsy, and lymphangitis. PA exhibits significant anticancer activities against several cancer cell lines in vitro, including human liver cancer, cervical cancer, and breast cancer cells, via downregulation of STAT3 and glycometabolism-related gene expression [17–19]. However, the current understanding of PA’s anticancer mechanisms is limited, mainly focusing on its ability to induce apoptosis and cause cell cycle arrest in vitro. In the current study, we discovered that PA induces pyroptosis in ATC cells and other cancer cells, suggesting that PA may have a distinct anticancer mechanism. This finding highlights the urgent need for in-depth research on the anticancer actions and mechanisms of PA, which could potentially lead to the development of a new candidate drug and therapeutic strategy for the treatment of ATC and other cancers.

The present study shows that PA effectively inhibits the growth of ATC cells both in vitro and in vivo via activating caspase 8/3/GSDME-pyroptosis of ATC cells through the induction of LMP and lysosomal damage. Mechanistically, PA excessively upregulates three key functional subunits of V-ATPase—ATP6V1A, ATP6V1B2, and ATP6V0C—leading to lysosomal over-acidification, which results in LMP and lysosomal damage with the release of cathepsins in ATC cells. These findings reveal a novel role for PA as a V-ATPase agonist that modulates lysosomal acidification, providing preclinical evidence for its potential use in ATC treatment.

## Results

### PA induces ATC cell death without PARP cleavage

**Fig. 1:**
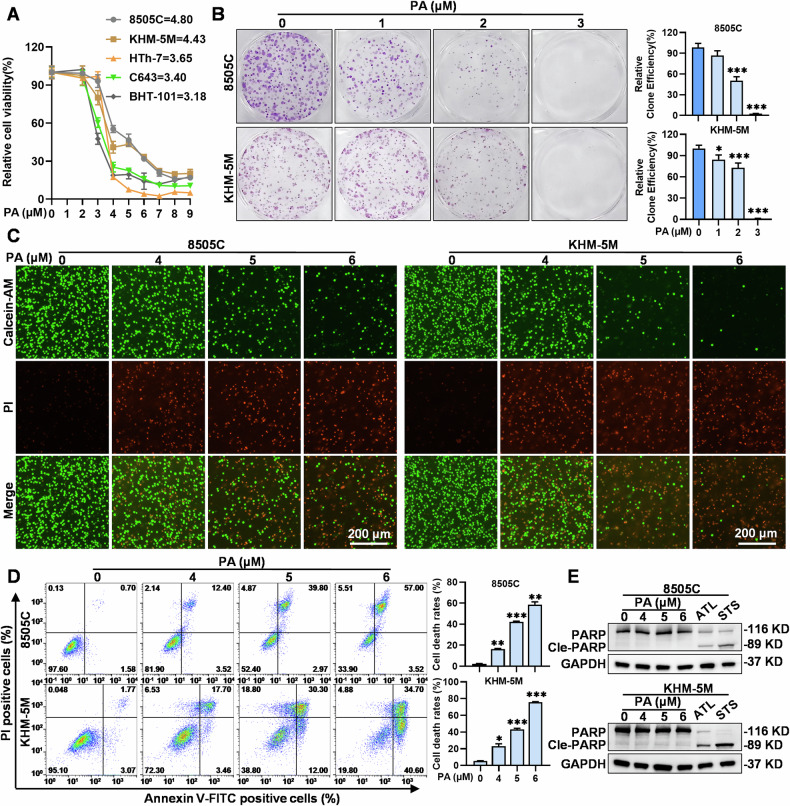
PA induces ATC cell death without PARP cleavage. **A** ATC cells (8505C, KHM-5M, C643, BHT-101, HTh-7) were treated with 0, 2, 3, 4, 5, 6, 7, 8, and 9 μM PA for 24 h. Relative cell viability was assessed by CCK8. **B** The clone formation images of 8505C and KHM-5M after PA treated at 0, 1, 2, 3 μM for 2 weeks, and quantified by the ImageJ software **C** 8505C and KHM-5M cells death was detected by Calcein-AM/PI Double Stain Kit, and morphologies were photographed by an Invitrogen EVOS-M 7000 microscope, after PA treated for 24 h at indicated concentrations, (scale bar: 200 μm). **D** 8505C and KHM-5M cell death was measured by flow cytometry after PA treatment for 24 h at 0, 4, 5, 6 μM. **E** PARP, cleaved-PARP and GAPDH protein levels in PA (0, 4, 5, 6 μM), ATL (20 μM) and STS (0.5 μM) treated for 24 h ATC cells (8505C and KHM-5M) were detected by western blot. Data are shown as mean ± SD for n = 3 (biological replicates). *p < 0.05, **p < 0.01, ***p < 0.001.

To investigate the anticancer effects of PA in ATC cells, we performed a Cell-Counting Kit-8 (CCK-8) assay assess the viability of five human ATC cell lines (8505C, KHM-5M, C643, BHT-101, and HTh-7) and five immortalized human normal tissue cell lines (thyroid follicular epithelial: Nthy-ori 3-1; gastric epithelial: GES-1; hepatocyte: HHL-5; breast epithelial: MCF-10A; keratinocyte: HaCaT) treated with PA at the indicated concentrations and timepoints, respectively (Fig. 1A, Supplementary Figs. S1, S2A). PA significantly inhibited the viability of the tested ATC cells in a dose- and time-dependent manner, and the IC_50_ values ranged from 3.18 μM to 4.80 μM (Fig. 1A, Supplementary Fig. S2A). In contrast, PA also inhibited the viability of the tested immortalized cell lines with higher IC_50_ values in the thyroid (5.68 μM) and breast (6.99 μM) epithelial cells compared to the ATC cells (Supplementary Fig. S1). Additionally, PA treatment significantly reduced the colony formation of 8505C and KHM-5M cells (Fig. 1B) and caused a minor increase in the proportion of G2/M phase cells in ATC cells (Supplementary Fig. S2B). Notably, PA-treated ATC cells exhibited rapid and dramatic morphological changes and detachment from the culture dish (data not shown), suggesting that PA’s cytotoxic activity might be the primary factor inhibiting ATC cell viability. By utilizing Calcein-AM/PI double staining, we confirmed that PA significantly induced concentration-dependent cell death in ATC cells, evidencing by a notable decrease in green viable cells and an increase in red non-viable cells (Fig. 1C). This was consistent with annexin V-FITC/PI assay results using flow cytometry, which showed that PA significantly induced dose- and time-dependent cell death, with visibly increased proportion of both annexin V-positive and PI-positive cells (Fig. 1D, Supplementary Fig. S3A, B). Intriguingly, PA-treated ATC cells exhibited negative PARP cleavage or downregulation, a marker typically associated with apoptosis (Fig. 1E, Supplementary Fig. S3C). In contrast, positive controls, such as alantolactone (ATL, an active ingredient from a traditional Chinese medicine against ATC) [20] and staurosporine (STS, an apoptosis inducer) [21], significantly downregulated pro-PARP protein levels and induced PARP cleavage (Fig. 1E). This suggested that PA-induced non-apoptotic cell death in ATC cells. Overall, these results indicated that PA inhibited the growth of ATC cells by inducing non-apoptotic cell death.

### PA elicits GSDME-dependent pyroptosis in ATC cells

**Fig. 2:**
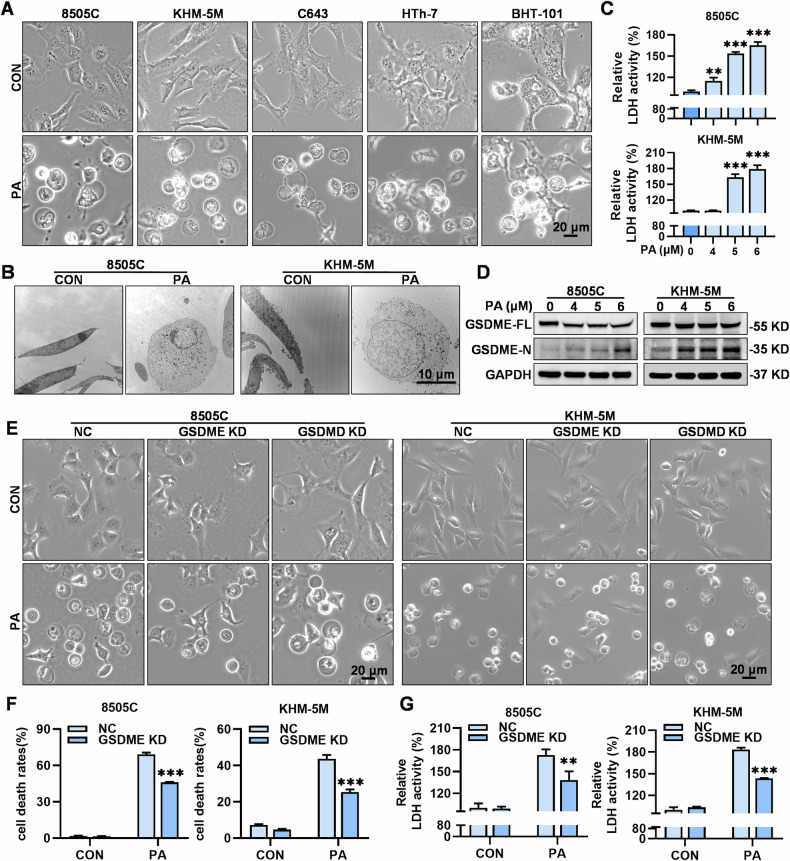
PA elicits GSDME-dependent pyroptosis in ATC cells. **A** Morphologic alterations of 8505C, KHM-5M, C643, BHT-101and HTh-7 cells induced by PA (5 μM) (scale bar: 20 μm). **B** 8505C and KHM-5M cells were treated with PA (5 μM) for 24 h and processed for electron microscopy, (scale bar: 10 μm). **C** After PA (0, 4, 5 and 6 μM) treatment for 24 h, relative LDH activity in culture mediums of 8505C and KHM-5M cells were detected by the LDH assay kit, **D** and the GAPDH, full-length GSDME and GSDME-N terminus protein levels were measured by western blot. **E** After knockdown (KD) GSDME and GSDMD, 8505C and KHM-5M cells were treated with PA (5 μM), morphologies were photographed by a phase contrast microscopy (scale bar: 20 μm). **F** After GSDME-KD, 8505C and KHM-5M cells were treated with PA (5 μM) for 24 h, cell death rates were assessed by flow cytometry, and **G** LDH activitiy in culture mediums was detected by the LDH assay kit. Data are shown as mean ± SD for *n* = 3 (biological replicates). **p < 0.01, ***p < 0.001.

The observation that PA-induced cell death in ATC cells without PARP cleavage prompted us to investigate the specific type of cell death involved. Neither ferroptosis inhibitor ferrostatin-1 nor necroptosis inhibitor necrosulfonamide restored the cell viability in PA-treated ATC cells, indicating that PA induced other forms of cell death (Supplementary Fig. S4A, B). Notably, we observed that all the tested ATC cells (8505C, KHM-5M, C643, HTh-7, and BHT-101) treated with PA exhibited swelling and balloon-like morphology, ultimately leading to resulting in cell membrane rupture (Fig. 2A), a primary phenotypic characteristic of pyroptosis. Similarly, this balloon-like morphology was also observed in other human cancer cell lines (gastric cancer: AGS; pancreatic cancer: PANC-1) treated with PA (Supplementary Fig. S5). Transmission electron microscopy confirmed that PA-induced noticeable cell swelling and pore formation in the membranes of both 8505C and KHM-5M cells (Fig. 2B). Moreover, lactate dehydrogenase (LDH) activity in the culture supernatant of PA-treated ATC cells was significantly increased in a dose- and time-dependent manner (Fig. 2C, Supplementary Fig. S6A). Previous studies have utilized the increase in annexin V^−^/PI^+^ and/or annexin V^+^/PI^+^ cells as one of the auxiliary indicators of pyroptosis [22, 23]. Consequently, we further analyzed the annexin V-FITC/PI assay results for PA-treated ATC cells. PA significantly elevated the proportion of both annexin V^−^/PI^+^ and annexin V^+^/PI^+^ cells (Supplementary Fig. S6B), indicating that pyroptosis might be involved in PA-induced ATC cell death. All these results demonstrated that PA-induced extensive pyroptosis in ATC cells.

Previously, we confirmed that GSDME expression was elevated in ATC cell lines, rendering ATC cells GSDME pyroptosis-prone [20]. We further sought to confirm whether PA-induced cleavage of GSDME, including other gasdermin family proteins GSDMC and GSDMD, which have received much attention in tumor research [24]. As expected, PA induced the dose-dependent cleavage of GSDME, but not GSDMC or GSDMD, in ATC cells (8505C and KHM-5M) (Fig. 2D, Supplementary Fig. S7A). Knockdown of GSDME substantially attenuated PA-induced pyroptosis in both 8505 C and KHM-5M cells, as evidenced by a distinct reduction in balloon-like morphology, LDH release, cell death ratio, and proportion of annexin V^−^/PI^+^ and annexin V^+^/PI^+^ cells (Fig. 2E–G, Supplementary Fig. S7B, C, D). Meanwhile, GSDMD knockdown did not reverse PA-induced pyroptosis of ATC cells (Fig. 2E, Supplementary Fig. S8). In addition, GSDME knockdown did not result in the cleavage of either GSDMD or PARP in the PA-treated cells (Supplementary Fig. S7E). These results demonstrated that PA elicited GSDME-dependent pyroptosis in ATC cells.

### PA induced GSDME-dependent pyroptosis through the caspase 8/3 pathway

**Fig. 3:**
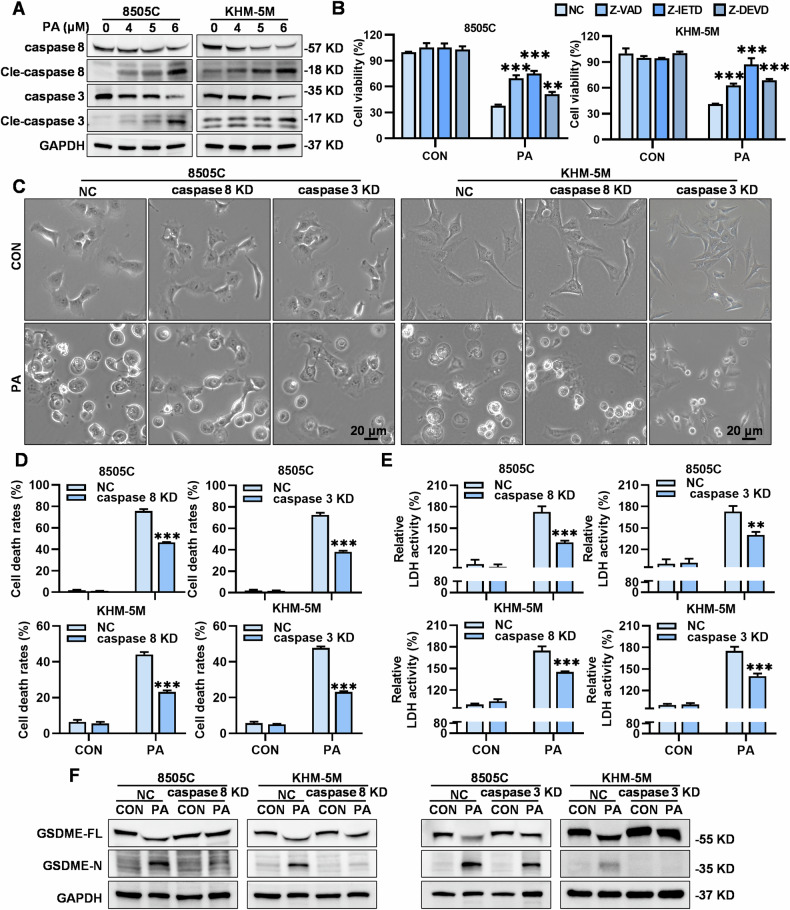
PA induced GSDME-dependent pyroptosis through the caspase 8/3 pathway. **A** After PA (0, 4, 5 and 6 μM) treatment for 24 h in 8505C and KHM-5M cells, the GAPDH, caspase 8/3, and cleaved-caspase 8/3 protein levels were measured by western blot. **B** PA (5 μM) is used in combination with Z-VAD (10 μM, pre-treated for 2 h), Z-IETD (10 μM, pre-treated for 2 h) and Z-DEVD (10 μM, pre-treated for 2 h) for 24 h in 8505C and KHM-5M cells, the cell viability was detected by the CCK8-assay. **C** After knockdown caspase 8/3 of 8505C and KHM-5M cells, and treated with PA (5 μM) for 24 h, morphologies were captured by microscope, (scale bar: 20 μm), and **D** cell death rates were assessed by flow cytometry, and **E** relative LDH activity in culture mediums detected by the LDH assay kit, and **F** moreover the GAPDH, full-length GSDME and GSDME-N terminus protein levels were measured by western blot. Data are shown as mean ± SD for *n* = 3 (biological replicates). **p < 0.01, ***p < 0.001.

After confirming the role of GSDME in PA-induced pyroptosis, we investigated whether caspase 8/3 or caspase 9/3 activation served as the primary executor of GSDME cleavage in PA-induced pyroptosis. PA dramatically induced the cleavage of caspases 8 and 3 in a dose-dependent manner (Fig. 3A) but did not induce the cleavage of caspase 9 in 8505C and KHM-5M cells (Supplementary Fig. S9A). Further analysis revealed that knockdown of caspase 9 scarcely reduced the PA-induced cell death ratio in both 8505C and KHM-5M cells, indicating that caspase 9 was not involved in PA-induced pyroptosis (Supplementary Fig. S9B, C). Therefore, we employed Z-VAD (a pan-caspase inhibitor), Z-IETD (a caspase 8 inhibitor), and Z-DEVD (a caspase 3 inhibitor) to evaluate the role of caspase 8/3 cleavage in PA-induced pyroptosis. Indeed, inhibition of pan-caspases, caspase 8, or caspase 3 significantly reversed the inhibitory effects of PA on 8505C and KHM-5M cells, including the restoration of cell viability (Fig. 3B, Supplementary Fig. S10A), reduction of “bubble-like” cells (Supplementary Fig. S10B), and decreased LDH release (Supplementary Fig. S10C). These results were consistent with the effects observed following the specific knockdown of caspase 8 or caspase 3 in PA-treated 8505C and KHM-5M cells (Supplementary Fig. S11A), which showed significant declines in “bubble-like” cells (Fig. 3C), cell death rates (Fig. 3D, Supplementary Fig. S11B), LDH release (Fig. 3E), and GSDME cleavage (Fig. 3F). Furthermore, caspase 8 knockdown did not result in the cleavage of caspase 9 but significantly reversed the cleavage of caspase 3 (Supplementary Fig. S11C). Taken together, these results revealed that PA-induced caspase 8/3/GSDME-dependent pyroptosis in ATC cells.

### PA mediated lysosome-related pyroptosis in ATC cells

**Fig. 4:**
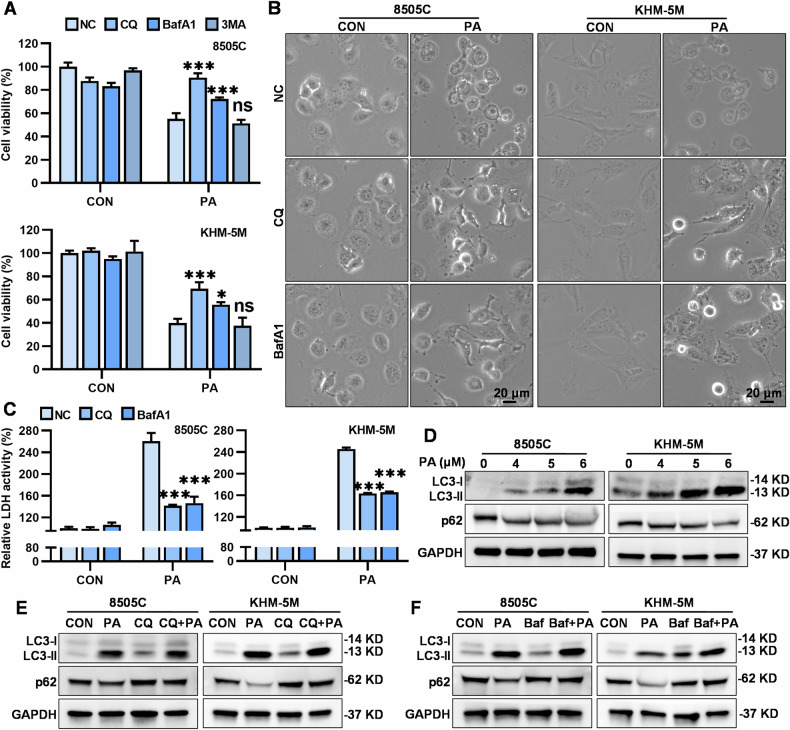
CQ and BafA1, instead of 3-MA, protected ATC cells from PA-induced pyroptosis. **A** In 8505C and KHM-5M cells, PA (5 μM) is used in combination with 3-MA (3 mM, pre-treated for 2 h), BafA1 (100 nM, pre-treated for 2 h) and CQ (10 μM, pre-treated for 2 h) for 24 h, cell viability was measured by the CCK-8 assay. In 8505C and KHM-5M cells, PA (5 μM) is used in combination with BafA1 (100 nM, pre-treated for 2 h) and CQ (10 μM, pre-treated for 2 h) for 24 h, **B** morphologies were captured by microscope (scale bar: 20 μm), **C** relative LDH activity in culture mediums detected by the LDH assay kit. **D** After PA (0, 4, 5 and 6 μM) treatment for 24 h, the GAPDH, LC3-I/II, and p62 protein levels were measured by western blot. **E**, **F** In 8505C and KHM-5M cells, PA (5 μM) is used in combination with BafA1 (100 nM, pre-treated for 2 h) and CQ (10 μM, pre-treated for 2 h) for 24 h, and the GAPDH, LC3-I/II, and p62 protein levels were measured by western blot. Data are shown as mean ± SD for *n* = 3 (biological replicates). *p < 0.05, ***p < 0.001, ns, *p* > 0.05.

PA induced caspase 8 cleavage rather than caspase 9 cleavage, suggesting that PA probably induced mitochondria-independent pyroptosis in ATC cells [25, 26]. We thus proceeded to investigate the specific mechanisms by which PA-activated caspase 8/3/GSDME-dependent pyroptosis of ATC cells. We explored several potential pathways known to be involved in cell death processes, including the classic death receptor pathway [27], RIPK1 pathway [28], autophagy-lysosome pathway [29], and reactive oxygen species (ROS) [30]. Notably, only the autophagy inhibitor Bafilomycin A1 (BafA1, which inhibits the fusion of autophagosomes with lysosomes by targeting V-ATPase) and chloroquine (CQ, which inhibits the fusion of autophagosomes with lysosomes by increasing lysosomal pH) [31, 32] significantly restored the cell viability and reversed pyroptosis phenotypes and the activation of the caspase 8/3-GSDME pathway in PA-treated 8505 C and KHM-5M cells (Fig. 4A–C, Supplementary Fig. S12A, B). Similar restoration of cell viability by BafA1 and CQ was observed in PA-treated AGS and PANC-1 cells (Supplementary Fig. S13), indicating a potentially common mechanism across various cancer cell lines. PA did not activate the death receptors (DR4 and DR5) and RIPK1 (Supplementary Fig. S14A), and neither the ROS scavenger N-acetyl cysteine (Supplementary Fig. S4A) nor RIPK1 knockdown (Supplementary Fig. S14B, C) could reverse the anti-ATC action of PA.

It appeared that PA induced a form of “autophagic cell death” in ATC cells by activating the autophagy-lysosome pathway. Autophagic cell death is a type of cell death that can be suppressed by inhibiting autophagy using autophagy inhibitors (e.g., 3-methyladenine (3MA), which inhibits class III Ptdln3K (phosphatidylinositol 3-kinase) and reduces the formation of Ptdln3P on the phagophore membrane, which is needed for the extension of phagophore membrane and the initiation of autophagy) [33] or through genetic ablation of essential autophagy genes [34]. We observed a significant increase in LC3-II protein level and a decrease in p62 protein expression in both PA-treated 8505C and KHM-5M cells (Fig. 4D), in addition, when PA was combined with CQ and BafA1, the protein levels of LC3-II and p62 were further enhanced (Fig. 4E, F), indicating increased autophagic flux. However, the autophagy inhibitor 3-methyladenine could not restore the cell viability of PA-treated ATC cells (Fig. 4A), although the autophagy flow activated by PA was blocked. These results suggested that PA-induced cell death in ATC cells may be related to lysosomes only.

**Fig. 5:**
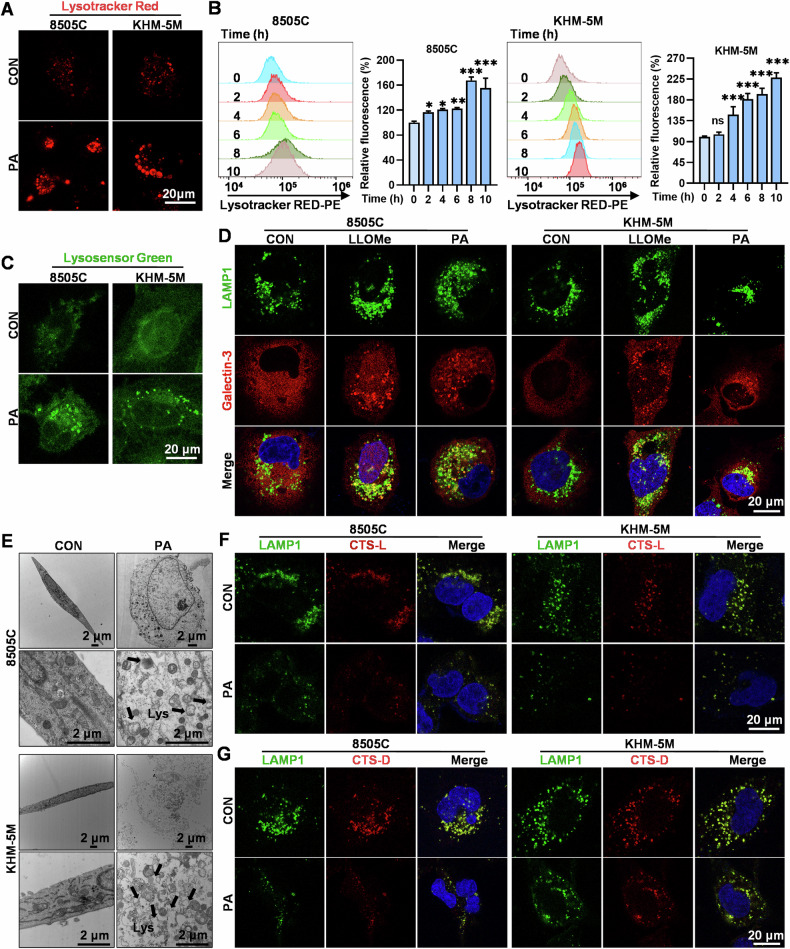
PA mediated lysosome-related pyroptosis in ATC cells. **A**, **C** After treated with PA (5 μM) for 8 h of 8505C and KHM-5M cells, stained by Lysotracker Red and Lysosensor Green DND-189, the morphologies were determined by confocal microscopy (Scale bar: 20 μm). **B** The 8505C and KHM-5M cells treated with 5 μM PA for 0, 2, 4, 6, 8, 10 h were stained by lysotracker Red and detected by flow cytometry, Histograms of corresponding mean fluorescence intensity (MFI) are shown. **D** The 8505C and KHM-5M cells were treated with PA (5 μM) and LLOMe (1 mM) for 12 h respectively, the immunofluorescence analysis was performed with anti-LAMP1 antibody (green), anti-Galectin-3 antibody (red), and DAPI (blue), the morphologies were determined by confocal microscopy, (scale bar: 20 μm). **E** 8505C and KHM-5M cells were treated with 5 μM PA for 24 h, and processed for electron microscopy, Lys, lysosome (scale bar: 2 μm). **F**, **G** The 8505C and KHM-5M cells were treated with PA (5 μM) for 12 h. The immunofluorescence analysis was performed with anti-LAMP1 antibody (green), anti-CTS-L antibody (red), anti-CTS-D antibody (red), and DAPI (blue), the morphologies were determined by confocal microscopy (scale bar: 20 μm). Data are shown as mean ± SD for *n* = 3 (biological replicates). *p < 0.05, **p < 0.01, ***p < 0.001, ns, *p* > 0.05.

Considering that PA-induced autophagy was not involved in pyroptosis induction in ATC cells and the observation that lysosomal inhibition with CQ and BafA1 restored cell viability of ATC cells, we directed our attention toward the changes in lysosomes that might be associated with PA-induced pyroptosis. Indeed, the lysosomes of 8505C and KHM-5M cells tremendously became enlarged and swollen after treatment with PA (Fig. 5A), with significantly increased red fluorescence (Lyso-Tracker Red) in a time-dependent manner (Fig. 5B). This was consistent with the increased green fluorescence in lysosomes (Lysosensor Green DND-189) (Fig. 5C). These results point to a significant increase in lysosomal acidification and lysosomal damage induced by PA, possibly accompanied by significant LMP, one of the most important phenotypes associated with lysosomal damage.

Galectin-3, a β-galactoside-binding cytosolic lectin, translocates from the cytosol to lysosomes during LMP and has been universally recognized as a valuable marker of LMP [35]. Galectin-3 staining identifies individual leaky lysosomes early during lysosomal cell death and proves valuable in determining whether LMP is a primary factor in cell death [36]. By employing galectin-3 immunofluorescence staining, we observed a remarkable translocation of galectin-3 from the cytosol to the lysosomes induced by PA in both 8505C and KHM-5M cells, consistent with observations with the positive control (L-Leucyl-L-Leucine methyl eater, LLOMe, an LMP inducer) (Fig. 5D). Transmission electron microscopy also confirmed evident lysosomal damage in PA-treated 8505C and KHM-5M cells, with many enlarged and swollen lysosomes (Fig. 5E). Furthermore, immunofluorescence staining of cathepsin L (CTS-L) and cathepsin D (CTS-D) (Fig. 5F, G) showed a significant decrease in the fluorescence signal intensity of lysosomes, indicating the marked release of lysosomal cathepsins into the cytosol caused by PA-induced LMP [15]. Upon introducing the cathepsins inhibitors pepstatin A and Z-FY-CHO (inhibit CTS-D and CTS-L, respectively), we observed a significant moderation of both the decline of cell viability (Supplementary Fig. S15A) and the activation of the caspase 8/3-GSDME pathway (Supplementary Fig. S15B) induced by PA in ATC cells. Therefore, these results indicated the released lysosomal cathepsins participated in the activation of the caspase 8/3-GSDME pathway in PA-treated cells. Take together, PA induces lysosomal over-acidification and resultant exacerbation of LMP and lysosomal damage, which ultimately leads to pyroptosis of ATC cells.

### V-ATPase overactivation-mediated lysosomal over-acidification and LMP drives pyroptosis in PA-treated ATC cells

**Fig. 6:**
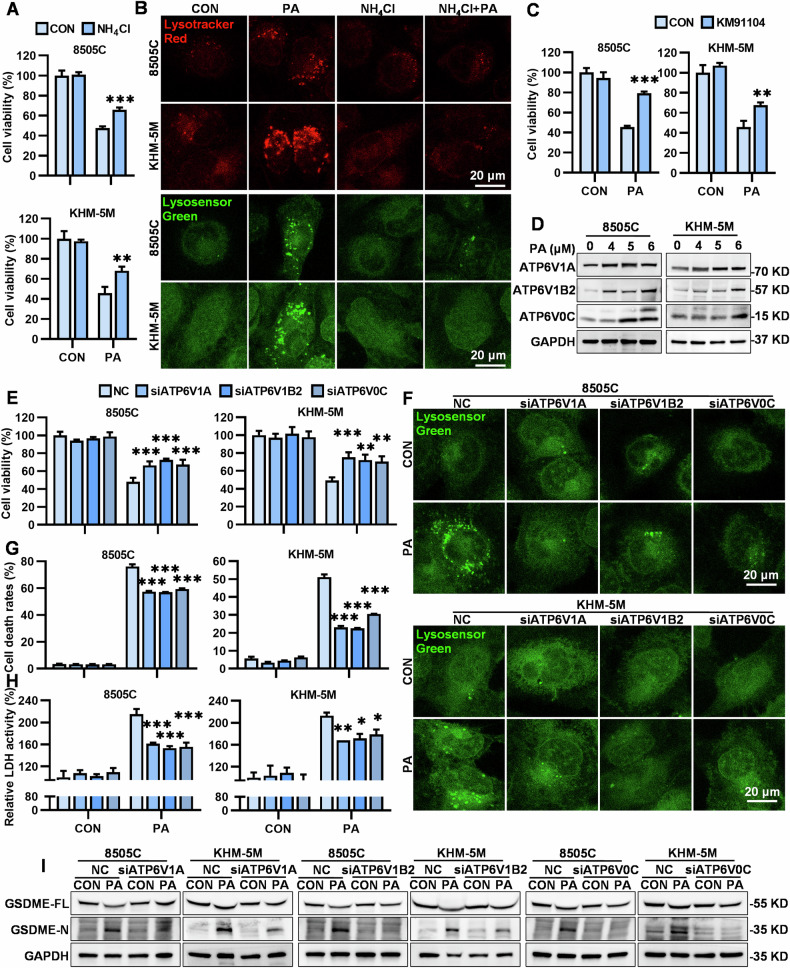
V-ATPase overactivation mediated lysosomal over-acidification and LMP drives pyroptosis in PA-treated ATC cells. **A** In 8505C and KHM-5M cells, cell viabilities following treatment with PA (5 μM) for 24 h in the presence or absence of NH_4_Cl (20 μM, pre-treated for 2 h) determined by the CCK-8 assay kit. **B** In 8505C and KHM-5M cells, PA (5 μM) was used in combination with NH_4_Cl (20 μM, pre-treated for 2 h) for 8 h, and stained by Lysotracker Red and Lysosensor Green DND-189, the morphologies were captured by confocal microscopy, (scale bar: 20 μm). **C** In 8505C and KHM-5M cells, cell viabilities following treatment with PA (5 μM) for 24 h in the presence or absence of KM91104 (1 nM, pre-treated for 2 h) determined by the CCK-8 assay kit. **D** After PA (0, 4, 5 and 6 μM) treatment for 24 h, the GAPDH, ATP6V1A, ATP6V1B2 and ATP6V0C protein levels were measured by western blot. **E** The 8505C and KHM-5M cells transfected with ATP6V1A, ATP6V1B2 and ATP6V0C si-RNA, and were treated with 5 μM PA for 24 h, the cell viabilities were measured by the CCK-8 assay. **F** The 8505C and KHM-5M cells transfected with ATP6V1A, ATP6V1B2 and ATP6V0C si-RNA, and were treated with 5 μM PA for 8 h, and stained by Lysosensor Green DND-189, morphologies were captured by confocal microscopy to observe the lysosomal pH, (scale bar: 20 μm). **G** The 8505C and KHM-5M cells transfected with ATP6V1A, ATP6V1B2, ATP6V0C si-RNA, were treated with 5 μM PA for 24 h, cell death rates were assessed by flow cytometry, **H** relative LDH activity in culture mediums detected by the LDH assay kit, and **I** the GAPDH, full-length GSDME and GSDME-N terminus protein levels were measured by western blot. Data are shown as mean ± SD for *n* = 3 (biological replicates). *p < 0.05, **p < 0.01, ***p < 0.001.

As both BafA1 and CQ are supposed to increase the lysosomal pH and significantly restore the viability of PA-treated ATC cells, we introduced a weak base, ammonium chloride (NH_4_Cl), that rapidly increases lysosomal pH [15], to confirm whether over-acidification drives LMP and the resultant pyroptosis of ATC cells. Indeed, NH_4_Cl strongly reversed the cell viability of PA-treated 8505C and KHM-5M cells (Fig. 6A, Supplementary Fig. S16A). Additionally, a substantial reversal of cell viability by NH_4_Cl was observed in both PA-treated AGS and PANC-1 cells (Supplementary Fig. S13). Moreover, NH_4_Cl significantly reversed PA-induced positive lysosomal staining of Lyso-Tacker Red and Lysosensor Green DND-189 in both 8505C and KHM-5M cells (Fig. 6B), which indicated that NH_4_Cl increased the lysosomal pH. Furthermore, NH_4_Cl prevented the release of lysosomal CTS-L and CTS-D into the cytosol of PA-treated ATC cells (Supplementary Fig. S16B, C), demonstrating that NH_4_Cl restored the functions of lysosomes. Furthermore, NH_4_Cl significantly attenuated the activation of the caspase 8/3-GSDME pathway in PA-treated 8505C and KHM-5M cells (Supplementary Fig. S16D). All these results revealed that PA induced lysosomal over-acidification, which then led to LMP and pyroptosis of ATC cells.

V-ATPase, a crucial proton pump responsible for lysosomal acidification, maintains a low acidic range within the lysosome, which is essential for normal lysosomal functions and behaviors [37]. As a specific inhibitor of V-ATPase, BafA1 significantly restored the cell viability of PA-treated 8505C and KHM-5M cells and prevented ATC cells from pyroptosis induced by PA (Fig. 4A–C). This was consistent with observations using another specific inhibitor of V-ATPase, KM91104 (Fig. 6C, Supplementary Fig. S17A, B). Additionally, KM91104 also significantly reversed the cell viability of PA-treated PANC-1 cells (Supplementary Fig. S13), but not AGS cells (Supplementary Fig. S13), suggesting that there are functional differences in V-ATPase subunits among different tumor cell lines and that the subunits targeted by BafA1 might be more critical for PA’s pharmacological action. BafA1 reportedly binds to the ATPV0C subunit of the V-ATPase macro-complex and consequently inhibits acidification of the lysosome, and KM91104 specially targets the interaction between the a3 and B2 subunits of V-ATPase [38]. Based on the above findings, we speculated that V-ATPase activation might be involved in PA-induced over-acidification, which constituted the initial step of ATC cell pyroptosis. Therefore, we focused on the three subunits of V-ATPase ATP6V1A (the primary subunits of V-ATPase), ATP6V1B2, and ATP6V0C (which are the subunits of V-ATPase inhibited by BafA1 and KM91104). PA significantly upregulated the expression of the three subunits at both the mRNA and protein levels (Fig. 6D, Supplementary Fig. S18A), suggesting that V-ATPase was overactivated by PA. Furthermore, knockdown of each subunit (Supplementary Fig. S18A, B) restored the cell viability (Fig. 6E) of PA-treated 8505C and KHM-5M cells by preventing PA-induced lysosomal over-acidification (Fig. 6F) and PA-induced pyroptosis of ATC cells, with reversal of GSDME cleavage and LDH release (Fig. 6G–I, Supplementary Fig. S18C, D). The data above confirmed that PA overactivated V-ATPase by upregulating the three key subunits: ATP6V1A, ATP6V1B2, and ATP6V0C, resulting in lysosomal over-acidification, which promoted LMP and ultimately induced pyroptosis of ATC cells.

To further clarify that V-ATPase overactivation-mediated lysosomal over-acidification is a critical precursor event in PA-induced ATC pyroptosis, we conducted experiments focusing on the chronological order of the events, including the expression of the three key subunits of V-ATPase (ATP6V1A, ATP6V1B2, and ATP6V0C), lysosomal acidification, activation of the caspase 8/3-GSDME pathway, and autophagy activation. As we anticipated, ATP6V1A, ATP6V1B2, and ATP6V0C were all significantly upregulated initially at 4 h and remained steadily upregulated until 24 h under PA treatment (Supplementary Fig. S19A), accompanied by a notable increase in lysosomal acidification (Supplementary Fig. S19B). In contrast, the cleavage of caspase 8, caspase 3, and GSDME occurred significantly later, after 20 h of PA treatment (Supplementary Fig. S19C), which was also consistent with the timing of cell viability inhibition, induction of cell death, and LDH release (Supplementary Figs. S2A, S3A, B, S6A). This indicated that the activation of caspase 8/3-GSDME is a subsequent event following V-ATPase overactivation and lysosomal over-acidification. In addition, we observed an increase in LC3-II and a decrease in p62 (Supplementary Fig. S19D), which occurred much later than V-ATPase overactivation and lysosomal over-acidification, almost after 16 h of PA treatment, although they occurred slightly earlier than the activation of caspase 8/3-GSDME. This further suggested that autophagy might be a reactive and accompanying response to PA treatment. Collectively, the above data demonstrated that PA-induced V-ATPase overactivation and lysosomal over-acidification were crucial for inducing the activation of the caspase 8/3-GSDME pathway, subsequently triggering pyroptosis in ATC cells.

### PA treatment suppresses tumor growth in vivo

**Fig. 7:**
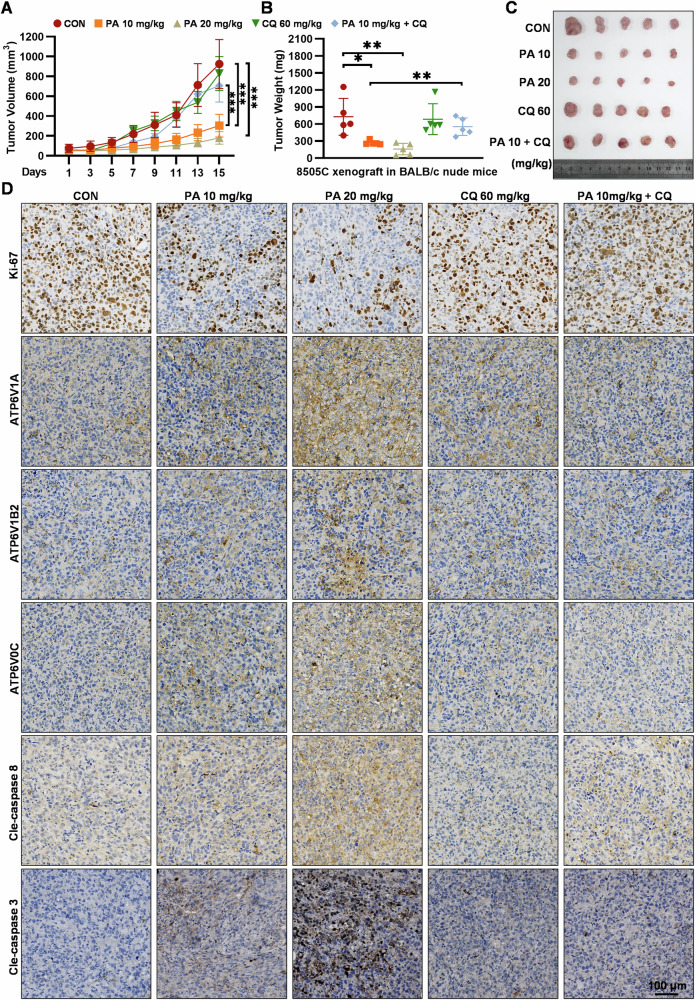
PA treatment suppresses tumor growth in vivo. **A** Growth curves of 8505C xenograft tumors in BALB/c nude mice treated with PA (10 mg/kg, 20 mg/kg), CQ (60 mg/kg), and PA (10 mg/kg) in combination with CQ (60 mg/kg) (*n* = 5/group), xenograft tumor volumes were measured every other day. **B** The tumors were dissected and weighted at day 15 after treatment. **C** Representative photographs of isolated tumors at day 15 after treatment. **D** Ki-67, ATP6V1A, ATP6V1B2, ATP6V0C, cleaved-caspase 8/3 expression levels in xenograft tumors as measured by IHC staining assay, (scale bar: 100 μm). Data are shown as mean ± SD for *n* = 5 (biological replicates). *p < 0.05, **p < 0.01, ***p < 0.001.

To explore the therapeutic effect of PA and confirm the anti-ATC mechanism of PA in vivo, a subcutaneous tumor model was constructed with 8505C cells. CQ was expected to be employed as an antagonist of PA in vivo, given its widespread use in clinical practice. Treatment with PA effectively suppressed tumor growth in a dose-dependent manner, as evidenced by a significant reduction in both tumor volume and weight compared with those in the control group (Fig. 7A–C). Notably, CQ significantly restored the growth of tumors treated with PA, whereas single CQ hardly affected the tumor growth (Fig. 7A–C). Meanwhile, each treatment had little effect on the body weight of nude mice (Supplementary Fig. S20A), pathological morphology of the liver, heart, and kidney tissues, and biochemical markers (aspartate aminotransferase, alanine aminotransferase, and creatinine) of liver and kidney functions (Supplementary Fig. S20B, C), indicating high safety and tolerance of PA in nude mice. Consistent with the in vitro findings, immunohistochemical staining demonstrated that treatment with PA promoted the expression of ATP6V1A, ATP6V1B2, ATP6V0C, and cleaved caspase 8/3, with the suppression of Ki-67, whereas CQ simultaneously relieved these effects of PA (Fig. 7D). Collectively, our findings demonstrated that PA could inhibit the growth of ATC tumors and activated V-ATPase-mediated lysosomal over-acidification-dependent pyroptosis of ATC cells without apparent drug toxicity, which indicated that PA might act as a promising candidate drug for treating ATC.

## Discussion

ATC is a highly aggressive and undifferentiated tumor, with a low patient survival rate and almost confirmed fatality [39]. The traditional treatments include surgery, radiation, and conventional chemotherapy. Despite recent advancements in targeted therapy and immunotherapy for ATC, treatment outcomes are often limited by the rapid development of tumor escape mechanisms and the emergence of drug resistance [40]. Consequently, there is a critical and pressing need to identify novel therapeutic targets or active compounds that can pave the way for innovative treatment strategies against ATC. These efforts are essential for improving patient outcomes and addressing the clinical challenges posed by this aggressive malignancy.

PA is a steroid saponin extracted from *Dioscorea zingiberensis*, *Veratrum*, and other plants that exhibits significant pharmacological effects [17, 41]. According to the Pharmacopoeia of the People’s Republic of China (1990 version), the traditional Chinese medicine “Fenbei Bixie,” which has been widely used to treat cervical, bladder, and kidney tumors, contains the tubers of *D. collettii var. hypoglauca*, known to contain PA [42]. While previous studies have highlighted PA’s ability to inhibit proliferation and induce apoptosis in human liver, nasopharyngeal, breast, and other cancer cells, its anticancer mechanisms remain poorly understood [17]. Particularly lacking are studies on its anti-ATC action and mechanism. In the present study, we found that PA significantly inhibited the growth of ATC cells by inducing activated caspase 8/3-GSDME-dependent pyroptosis without PARP cleavage. PA was found to activate V-ATPase activity by upregulating the three key subunits ATP6V1A, ATP6V1B2, and ATP6V0C, leading to the increased acidity of the lysosomal lumen, which resulted in LMP and lysosomal damage in ATC cells. Consequently, cathepsins in lysosomes were released into the cytoplasm, which activated cleavage of caspase 8 and caspase 3, ultimately inducing pyroptosis in ATC cells. Consistent with the in vitro results, PA effectively restrained the growth of subcutaneous xenograft ATC tumors, exhibiting similar mechanistic effects without causing any adverse effects. To our knowledge, this study is the first to comprehensively elucidate the anticancer actions and mechanisms of PA, particularly revealing its novel role in inducing lysosomal acidification-dependent pyroptosis in ATC cells. These findings suggest PA as a promising candidate for ATC treatment, opening new avenues for its anticancer application.

Pyroptosis was initially identified as a programmed cell death triggered by the inflammasome, in which GSDMD is cleaved by caspase 1 or caspase 11/4/5 into the N-terminal domain (PFD) and C-terminal domain (RD). PFD forms holes in the cell membrane, causing the cell to swell and decompose, accompanied by the release of pro-inflammatory contents such as IL-1β and IL-18 [43]. Specifically, caspase 3/GSDME cleavage-mediated pyroptosis in tumor cells has provided a new understanding that anticancer drugs inducing apoptosis may also have the potential to induce pyroptosis [44]. Additional research has indicated that both conventional chemotherapeutic drugs and Chinese medicine achieve their anticancer effects by inducing either pyroptosis or apoptosis, often accompanied by activated caspase 3/GSDME pyroptosis [20, 45]. Our group previously demonstrated that a natural active sesquiterpene lactone from *Inula helenium L*., ATL, exhibited effective anti-ATC activity by concurrently inducing apoptosis and GSDME-dependent pyroptosis, which has provided a novel perspective on apoptosis and pyroptosis co-mediated by the activation of caspase 3 [20]. In this study, we provided another novel insight into caspase 3-dependent pyroptosis of ATC cells induced by PA without apoptosis. In the same ATC cells, based on the different characteristics of apoptotic and pyroptotic cells, as well as PARP and GSDME cleavage status, PA-induced caspase 3/GSDME-dependent pyroptosis only, whereas ATL induced both caspase 3/PARP-dependent apoptosis and caspase 3/GSDME-dependent pyroptosis. This demonstrates an elegant modulation of caspase 3 in both apoptosis and pyroptosis. Identification of additional pyroptotic effectors in PA-induced ATC cell pyroptosis may require further investigation, as the knockdown of GSDME did not completely reverse the PA-induced pyroptotic phenotype, while GSDMC and GSDMD have been eliminated as candidates. Pyroptosis is considered a form of immunogenic cell death that activates anticancer immune responses, which can enhance anticancer immunotherapy [46]. This attribute may endow PA with additional potential anticancer effects when combined with anticancer immunotherapy.

Cytoprotective autophagy activation has been recognized in numerous cancer therapies, especially in chemotherapy and targeted therapy [47]. Through autophagy, cancer cells subjected to anticancer agents degrade subcellular organelles to provide energy and metabolic precursors, sustaining cell survival and avoiding elimination [47]. Inhibiting cytoprotective autophagy through pharmacologic or genetic approaches can enhance the therapeutic effects of anticancer agents [15, 36]. In the present study, we observed that PA-induced autophagy in ATC cells, and we hypothesized that this autophagy might provide protection against the cytotoxic effects of PA. However, our results that both CQ and BafA1 rather than 3MA restored the cell viability of PA-treated ATC cells and protected the cells from pyroptosis, indicating that inhibiting the initial stage of autophagy does not affect the anti-ATC action of PA. Given that CQ and BafA1 inhibit lysosomal activity and alter lysosomal pH, we are inclined to believe that the autophagy induced by PA might be a form of stress-related response.

Lysosomes are acidic organelles containing acid hydrolases, often referred to as “suicidal bag of cells,” playing a crucial role in regulating cell death [48]. Their functions are highly dependent on the acidic luminal pH, maintained in a narrow pH range (pH 4.5–5.0), essential for hydrolases activity [37]. This acidic environment is maintained by V-ATPase, which pumps H^+^ from the cytosol into the lysosomal lumen [10]. The activity of calpains and caspases aggrandizes the level of ROS, and the inhibition of V-ATPase induced LMP and lysosome damage, causing the leakage of internal environment components into the cytoplasm and resulting in cell death [49]. V-ATPase is extensively reported as a key regulatory factor for maintaining lysosomal functions. Pharmacologic or genetic inhibition of V-ATPase leads to LMP and various forms of cell death, including apoptosis, ferroptosis, anoikis, and alkaliptosis [50]. In contrast, herein, PA-induced activation of V-ATPase and the resultant pyroptosis of ATC cells were reversed by the V-ATPase inhibitor BafA1 and KM91104 as well as knockdown of the three key subunits ATP6V1A, ATP6V1B2, and ATP6V0C, respectively. This indicates that inhibition of V-ATPase protected ATC cells from the lethal effects of PA. Additionally, PA-induced lysosomal over-acidification was significantly reversed by these V-ATPase inhibitors. Concurrently, lysosomal over-acidification was neutralized by CQ and the weak base NH_4_Cl, which strongly restored the cell viability of PA-treated ATC cells. Messner et al. showed that cadmium contributes to hyper-acidification and permeabilization of lysosomes, ultimately inducing cell death; however, they did not indicate that over-acidification was the cause of the lysosomal permeabilization [16]. Chen et al. found that upregulation of ATP6V0D1, a V-ATPase subunit, altered lysosomal homeostasis and induced alkaliptosis by restraining signal transducer and activator of transcription 3 [51]. This is inconsistent with the idea that impaired lysosomal acidification by inhibiting V-ATPase leads to lysosome-dependent cell death, and lysosomal over-acidification by over-activating V-ATPase would also cause cell death. Furthermore, we provided a novel insight into LMP-dependent pyroptosis in PA-treated ATC cells caused by the activation of V-ATPase and lysosomal over-acidification.

## Materials and methods

### Cell culture and reagents

Human ATC cell lines (C643, KHM-5M, BHT-101, and HTh-7); human immortalized cell lines, including breast epithelial cell line MCF 10A, gastric epithelial cell line GES-1, and keratinocyte cell line HaCaT; human gastric cancer cell line AGS; and pancreatic cancer cell line PANC-1 were purchased from the National Collection of Authenticated Cell Cultures (Shanghai, China). Human immortalized hepatocyte cell line HHL-5 was a gift from Xinxin Ren [52]. Human ATC cell line 8505C was purchased from Deutsche Sammlung von Mikroorganismen und Zellkulturen (DSMZ), and human thyroid epithelial cell line Nthy-ori 3-1 was acquired from Procell Life Science & Technology (Wuhan, China). All cells had undergone STR authentication and were grown in RPMI-1640 medium supplemented with 10% fetal bovine serum (FBS) in a 37 °C humidified incubator with 5% CO_2_. PA was purchased from Chengdu Must Bio-Technology Co., Ltd (China); ATL was purchased from Topscience (China); N-acetyl cysteine (#S007) was purchased from Beyotime Institute of Biotechnology (China); 3-methyladenine (#T1879), CQ (#T0194), BafA1 (#T6704), necrosulfonamide (#T6904), Z-IETD (#T7019), PEG300 (#T7022), LLOMe (#T7739), KM91104 (#T9230), STS (#T6680), pepstatin A (#T3695), and Tween 80 (#T13947) were obtained from TargetMOI (USA); Z-VAD (#A1902) and Z-DEVD (#A1920) were purchased from ApexBio (USA); NH_4_Cl (#254134) was purchased from Sigma-Aldrich (USA); and Z-FY-CHO (CAS No: 167498-29-5) was purchased from MedChemExpress (USA).

### Cell viability and colony formation assay

PA cytotoxicity against ATC cells was assessed using CCK8 (#K1018, ApexBio, USA). The cells were seeded in 96-well plates (6000 cells/well) for 24 h, then treated with different concentrations (2, 3, 4, 5, 6, 7, 8, 9, 10 μM) of PA for 24 h. DMSO (1 μL/mL or less) was used for the control group. Next, 10 μL of CCK8 and 90 μL of RPMI-1640 containing 10% FBS were added to each well and incubated for ~2 h. Optical density at 450 nm was measured using Synergy LX Multi-Mode Reader (BioTek Instruments, USA), and the relative cell viability was calculated.

For the colony formation assay, 8505C and KHM-5M cells (2000 cells/well) were seeded in 6-well plates and treated with PA (0, 1, 2, 3 μM) for 2 weeks. Surviving cells were immobilized in methanol, stained with crystal violet, and photographed. The results were quantitatively analyzed using ImageJ software (National Institutes of Health, USA).

### Cell live/death assay

Briefly, 8505C and KHM-5M cells (2.0 × 10^5^ or 1.8 × 10^5^ cells per well) were seeded in 6-well plates, respectively. After treatment with indicated concentrations of PA for 24 h, an Annexin V-FITC/PI Kit (MultiSciences, Hangzhou, China) was used following the manufacturer’s instructions, and data were collected by a flow cytometer; FlowJo was used to analyze the data. Further, under the same PA treatment conditions, the cells were stained with a calcein-AM/PI double-stain kit (Yeasen Biotech Co., Ltd, Shanghai, China). Cells with green fluorescence were considered viable, and those with red fluorescence were considered dead. All images were captured using a fluorescence microscope (Thermo Fishier, Evos M7000, MA, USA).

### Cell morphological analysis

Briefly, 8505C, BHT-101, KHM-5M, C643, and HTh-7 cells (1.8 × 10^5^, 2.0 × 10^5^ cells per well) were seeded in 6-well plates, respectively. After PA (5 μM) treatment, the morphology of each cell with pyroptosis characteristics was examined using a phase-contrast inverted microscope (Nikon, Japan; Carl Zeiss, Germany).

For transmission electron microscopy, 8505C and KHM-5M cells were seeded in a 100 mm culture dish (2 × 10^6^ per dish). After treatment with PA (5 μM), cells were fixed with glutaraldehyde for 1 h (HaoKe Biotechnology Co.Ltd, Hangzhou, China) and post-fixed with osmium. Subsequently, the samples were dehydrated using a gradient ethanol and permeabilized with SPURR-containing acetone. Polymerization was performed at 70 °C for 24 h. Samples were cut into 60–80 nm ultra-thin slices and stained with 2% diuranium-acetate saturated alcohol solution and lead citrate. Finally, the cell plasma membrane and lysosomes were examined using a transmission electron microscope (Hitachi, Japan).

### LDH release assay

Pyroptosis was partially determined by examining the activity of LDH released into the cell culture supernatant. After 8505C and KHM-5M cells were treated with PA (0, 4, 5, 6 μM) for 24 h, the supernatant was collected and tested using an LDH assay kit (#A020-1; Nanjing Jiancheng Bioengineering Institute, Nanjing, China) according to the manufacturer’s protocol. The optical density at 450 nm was measured using Synergy LX Multi-Mode Reader and analyzed using GraphPad Prism software version 8.

### Lysosome staining and analysis

Changes in lysosomes in ATC cells (8505C and KHM-5M) were analyzed by staining with lysosomal fluorescent probes. After PA (5 μM) treatment, cells were incubated with a commercial lysosome staining dye Lyso-Tracker Red (Beyotime Institute of Biotechnology, Shanghai, China) and a commercial lysosomal pH-sensitive probe Lysosensor Green DND-189 (Yeasen Biotech Co., Ltd, China), respectively, at 37 °C for 30 min in the dark. After washing with phosphate-buffered saline (PBS), the processed cells were analyzed by flow cytometry (Agilent NovoCyte Advanteon, California, USA) or detected by confocal microscopy (Leica TCS SP8, Wetzlar, Germany).

### Immunofluorescence staining

ATC cells 8505C (5 × 10^4^ per well) and KHM-5M (3 × 10^4^ per well) were seeded in a quadrant culture dish. They were then treated with PA (5 μM), fixed with 4% paraformaldehyde for 15 min, and blocked with TBST (containing 3% BSA and 4% gelatin) for 1 h. The cells were then incubated with the following primary antibodies: anti-CTS-D (#ET1608-49, HuaAn Biotechnology, China), anti-CTS-L (#ET1703-44, HuaAn Biotechnology, China), anti-LAMP1 (#15665S, Cell Signaling Technology, USA), and anti-galectin-3 (#87985S, Cell Signaling Technology, USA) overnight at 4 °C. Subsequently, the samples were washed with PBS, incubated with an appropriate fluorescent secondary antibody at 25 °C in the dark for 1 h, and finally stained with DAPI (4 ‘, 6-Diaminidine-2 ‘-phenylindole) for 10 min. Images were captured by confocal microscopy.

### CRISPR/Cas9-mediated knockdown

The CRISPR/Cas9 lentivirus plasmids and corresponding lentiviral particles were constructed by Applied Biological Materials Inc., Canada. The sequences of the gRNAs used were 5′-GTATAACTCAATGACACCGT-3′ for GSDME, 5′-GGTAGTCCGGAGAGTGGTCC-3′ for GSDMD, 5′-ATTGTGGAATTGATGCGTGA-3′ for caspase 3, 5′-GCTCTTCCGAATTAATAGAC-3′ for caspase 8, 5′-GAACAGCTCGCGGCTCAGCAGGG-3′ for caspase 9, and 5′-GGCACCGCTAAGAAGAATGG-3′ for RIPK1. To generate knockdown cell lines, 8505C and KHM-5M cells were seeded in 6-well plates at a density of 1 × 10^5^ cells/well, and the lentiviral particles were transfected at a multiplicity of infection of 100. After 48 h, 10 μg/mL puromycin was added for screening and knockdown efficiency was verified by western blot. The highly efficient knockout cells were used for relevant rescue experiments without generating single cell-derived knockout clones.

### siRNA-mediated knockdown

For siRNA-mediated knockdown, 8505C and KHM-5M cells were plated in 6-well plates (2.0 × 10^5^ or 1.8 × 10^5^ cells per well) for 24 h. ATP6V1A siRNA: 5′-CAAAGACCTTTGTCGGATA-3′, ATP6V1B2 siRNA: 5′-CCCTCACTATCACGGTTAA-3′, ATP6V0C siRNA: 5′-CCAGCTATCTATAACCTTA-3′ or control siRNA was transfected with Lipofectamine™ 3000 transfection reagent (Invitrogen) following the manufacturer’s instructions; 48 h later, transfected ATC cells were treated with PA and subjected to analyses as indicated.

### Quantitative real-time PCR

Total RNA from the treated cells was extracted using RNAisoPlus (Takara, Japan), and cDNA was synthesized using PrimeScript^TM^ RT Master Mix (Takara, Japan). Quantitative real-time PCR was performed using Hieff^®^ qPCR SYBR Green Master Mix (Yeasen Biotech Co., Ltd, China) on LC-480II (Roche, Swiss). The sequences of the relevant primers were as follows: ATP6V1A (F: 5′-GGGTGCAGCCATGTATGAG-3′, R: 5′-TGCGAAGTACAGGATCTCCAA-3′), ATP6V1B2 (F: 5′-AGTCAGTCGGAACTACCTCTC-3′, R: 5′-CATCCGGTAAGGTCAAATGGAC-3′), and ATP6V0C (F: 5′-TGAATGACGACATCAGCCTCT-3′, R: 5′-CGGCGAAGATGAGAATCAGGAT-3′), and 18 S rRNA (F: 5′-AGGCCCTGTAATTGGAATGAGTC-3′; R: 5′-GCTCCCAAGATCCAACTACCAG-3′), which was used as the internal reference gene. The relative mRNA levels were calculated using the 2^–ΔΔCt^ method.

### Western blot

Cells treated with PA were lysed using western blot and IP lysis buffer (#P0013, Beyotime Institute of Biotechnology, China) containing phenylmethanesulfonylfluoride (PMSF) (100 µM). Total protein concentrations were measured using a bicinchoninic acid protein assay kit (Thermo Fisher Scientific, USA). The samples were separated by SDS-PAGE, transferred onto a PVDF membrane, and then blocked with TBST containing 5% skim milk for 1 h. The following primary antibodies diluted in TBST (containing 4% gelatin and 3% BSA) were used: anti-ATP6V1A (#17115-1-AP, 1:3000, Proteintech), anti-ATP6V1B2 (#15097-1-AP, 1:3000, Proteintech), anti-GAPDH (#60004-1-Ig, 1:5000, Proteintech), anti-caspase 8 (#13423-1-AP, 1:3000, Proteintech), anti-p-RIPK1 (#66854-1-Ig, 1:2000, Proteintech), anti-Cle-caspase 8 (#8592, 1:2000, Cell Signaling Technology), anti-DR4 (#42533, 1:2000, Cell Signaling Technology), anti-DR5 (#8074, 1:2000, Cell Signaling Technology), anti-RIPK1 (#3493, 1:2000, Cell Signaling Technology), anti-MLKL (#14993, 1:3000, Cell Signaling Technology), anti-p-MLKL (#91689, 1:2000, Cell Signaling Technology), anti-AT6V0C (#A23757, 1:3000, ABclonal), anti-active caspase 3 (#ET-1602-47, 1:500, HuaAn), anti-caspase 9 (#9502, 1:3000, Cell Signaling Technology), anti-PARP (#9532, 1:3000, Cell Signaling Technology), anti-GSDME (#ab215191, 1:2000, Abcam), anti-GSDMD (#ab209845, 1:2000, Abcam), anti-p62 (#ab207305, 1:3000, Abcam), anti-LC3B (#ab192890, 1:3000, Abcam), anti-caspase 3 (#ab32351, 1:3000, Abcam), and anti-GSDMC (#ap10771c, 1:500, Abcepta). After a 4 °C overnight incubation with the primary antibodies, the membranes were washed with TBST and incubated with the following secondary antibodies for 1 h at 25 °C: goat anti-rabbit (#A0208, 1:3000, Beyotime) and anti-mouse (#A0216, 1:3000, Beyotime) HRP IgG, and washed. Immunodetection was performed using an enhanced chemiluminescence kit (Fdbio Science, Hangzhou, China) and detected by SH-Cute 523 (Shenhua Biotech, Hangzhou, China).

### In vivo xenograft tumor model

Female nude mice (BALB/c, 3–4 weeks old) were purchased from GemPharmatech (Nanjing, China) and maintained at the laboratory animal center at Zhejiang Provincial People’s Hospital (Approval NO. IACUC-A20230309001) according to the requirements of feeding. One week later, subcutaneous implantation of 8505C cells (5 × 10^6^ cells/100 µL) was performed on the right axilla of the mice. Upon forming palpable tumors (~60 mm^3^), all mice were randomly divided into five groups (five in each group) with different treatments: vehicle (5% DMSO + 10% Tween-80 + 30% PEG300 + 55% PBS); PA (10 mg/kg) dissolved in 5% DMSO + 10% Tween-80 + 30% PEG300 + 55% PBS; PA (20 mg/kg) dissolved in the same solvent; CQ (60 mg/kg) dissolved in PBS; and CQ (60 mg/kg) + PA (10 mg/kg). These compounds were administered daily via an intraperitoneal injection. Moreover, the volume (0.5 × (shortest diameter)^2^ × (longest diameter)) and body weight were recorded every other day. After 14 days of treatment, all mice were sacrificed, and the tumors were removed and weighed. The tumor, heart, liver, and kidney tissues were fixed with 4% paraformaldehyde. Tumor tissues were analyzed using hematoxylin and eosin and immunohistochemical staining. The heart, liver, and kidneys were fixed with 4% paraformaldehyde, embedded in paraffin, stained with hematoxylin and eosin, and then used to evaluate the safety of PA in vivo.

### Immunohistochemical staining

Briefly, 4 μm-thick sections were cut from the paraffin-embedded tissue blocks, deparaffinized, rehydrated, and treated with citrate buffer (pH 6.0) for antigen retrieval. After incubation with 3% BSA for 30 min, the sample was incubated with the indicated primary antibody at 25 °C for 1 h and the corresponding HRP-labeled secondary antibody at room temperature for 30 min. Nuclei were stained with hematoxylin (blue), and positive 3,3’-diaminobenzidine expression was detected by a brownish-yellow color. Images were captured using a fluorescence microscope (Konfoong Biotech, Ningbo, China).

### Statistical analysis

All statistical analyses were performed using GraphPad Prism software version 8. The difference between groups was compared using one-way/two-way ANOVA or *t*-test. Data are shown as mean ± SD for *n* = 3, and *P*-value < 0.05 was considered statistically significant.

### Supplementary information

Original western blots

supplement figure

## Data Availability

The datasets used and/or analysis during the current study are available from the corresponding author on reasonable request.
