# Supplementary material for: Prosapogenin A induces GSDME-dependent pyroptosis of anaplastic thyroid cancer through vacuolar ATPase activation-mediated lysosomal over-acidification
Source: Cell Death Dis. 2024 Aug 13;15(8):586. doi: 10.1038/s41419-024-06985-z (PMC11322489; doi:10.1038/s41419-024-06985-z)

Original Western Blots for Figure 1E

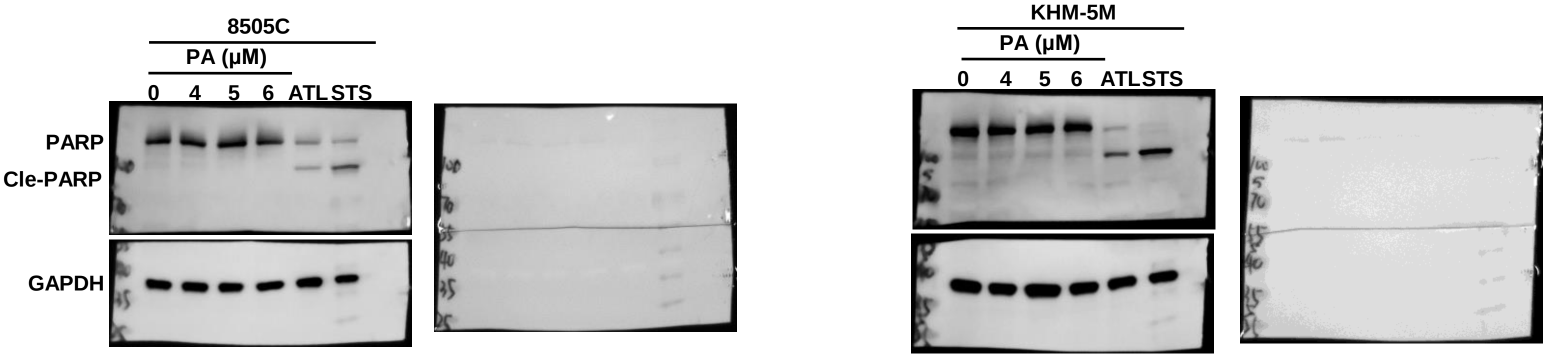

Original Western Blots for Figure 2D

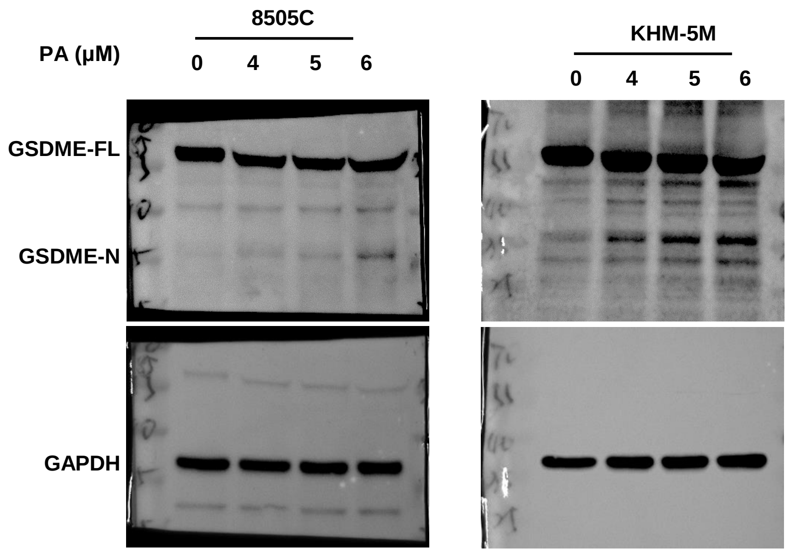

Original Western Blots for Figure 3A

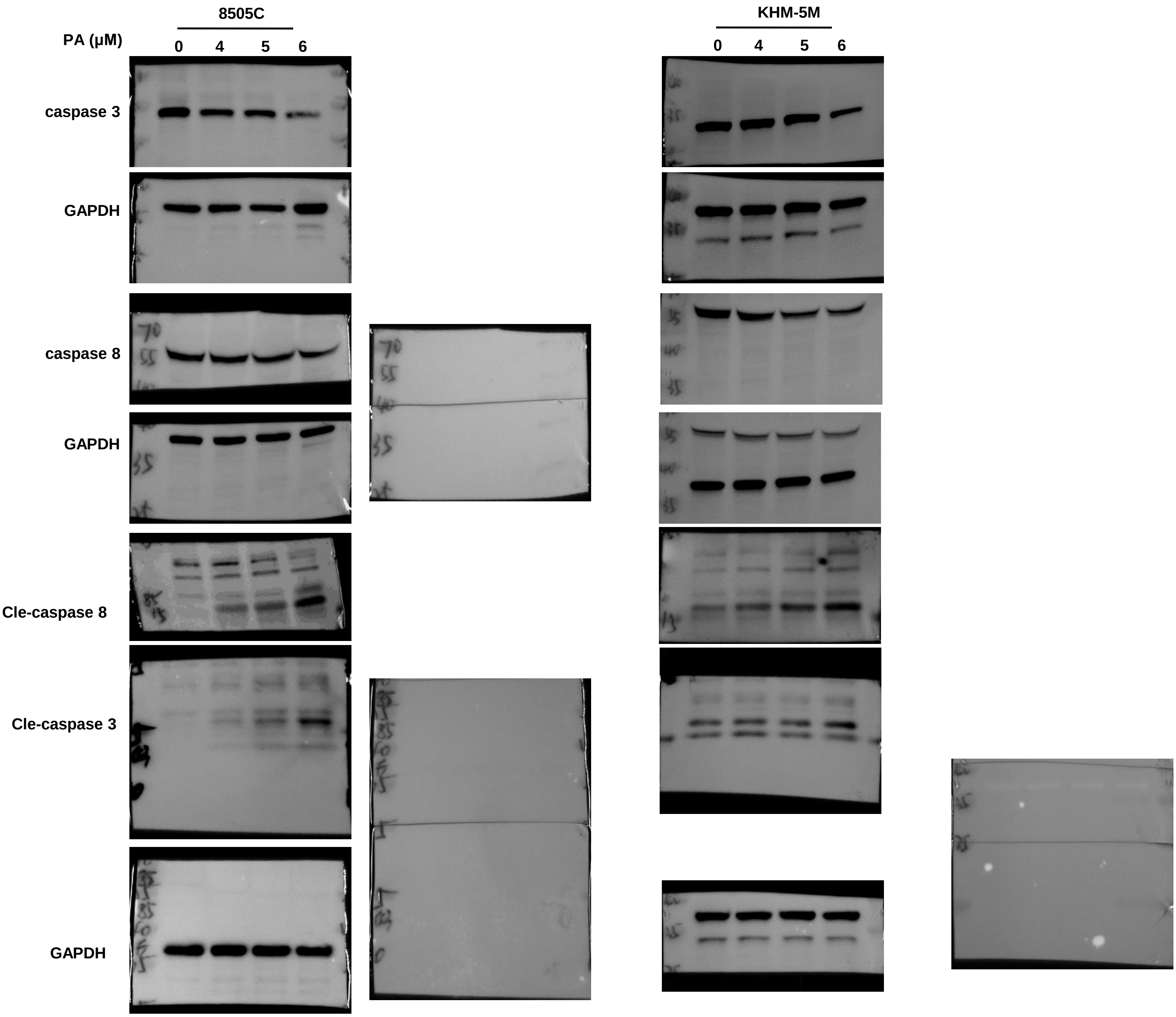

Original Western Blots for Figure 3F

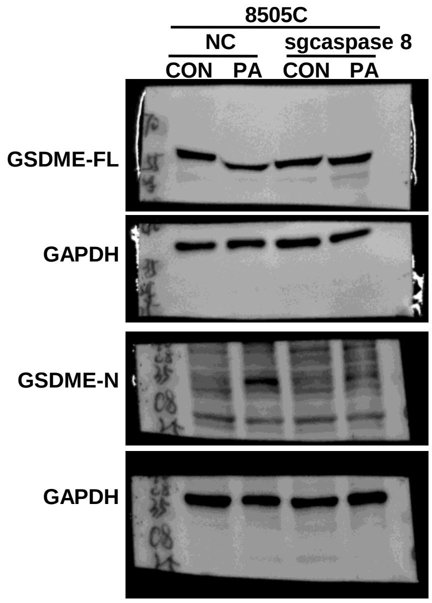

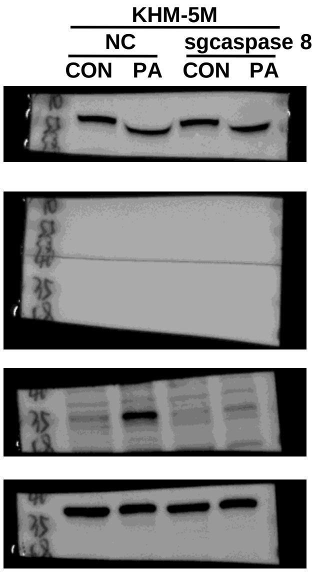

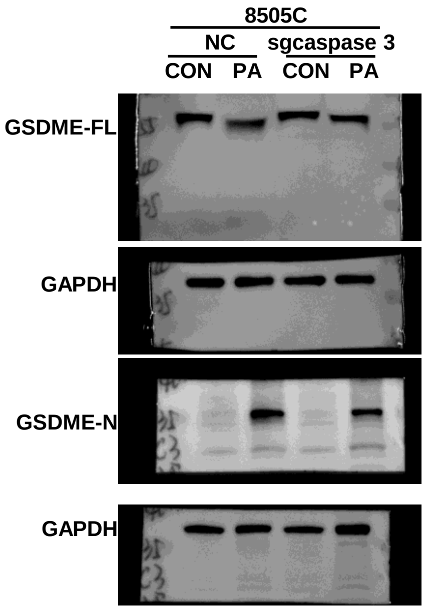

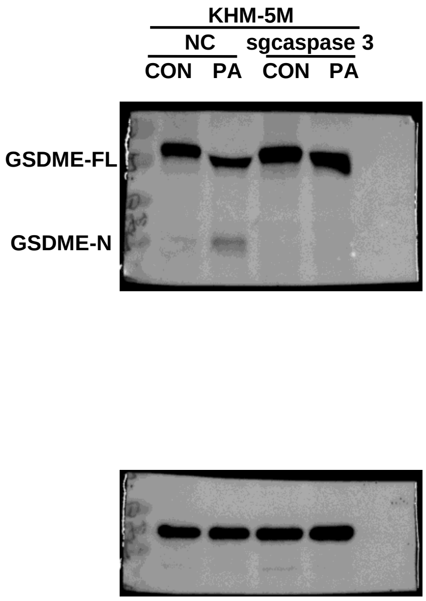

Original Western Blots for Figure 4D

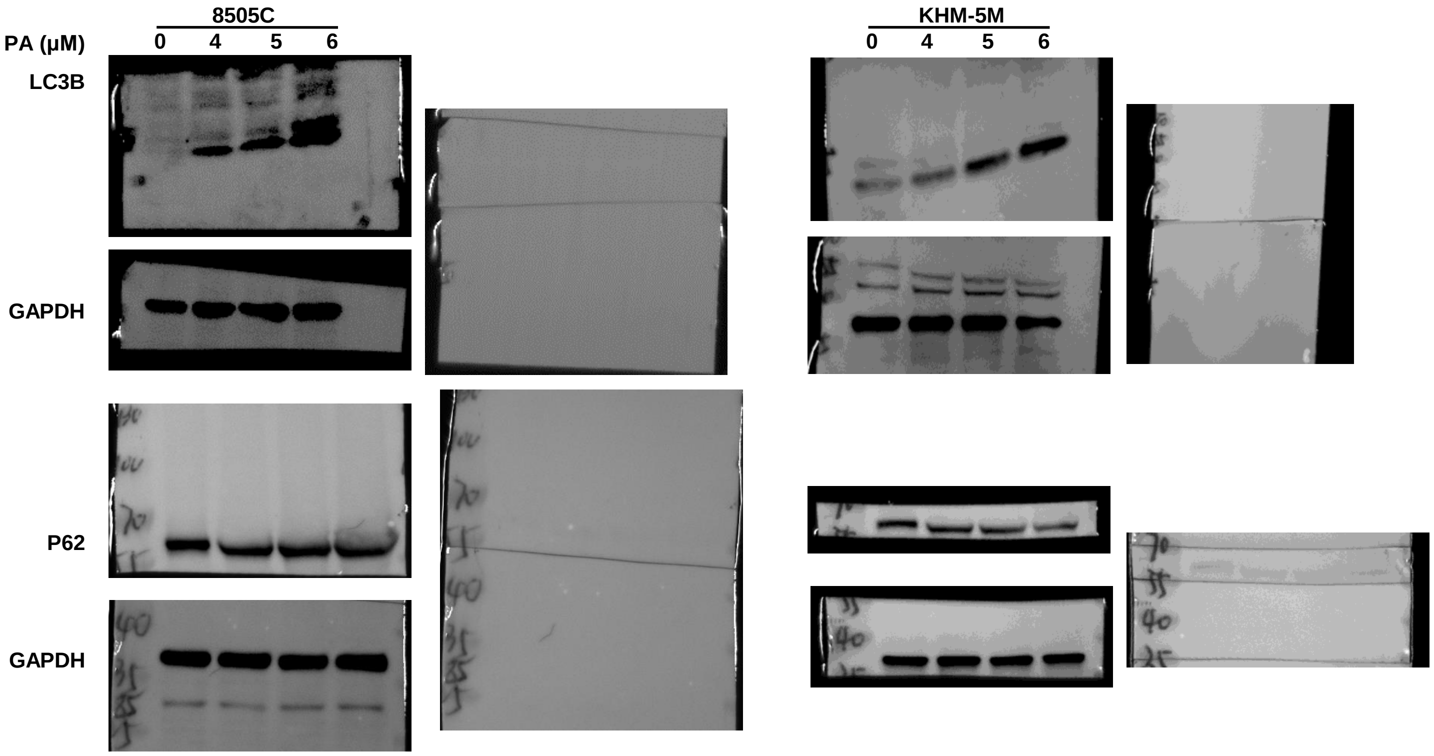

Original Western Blots for Figure 4E

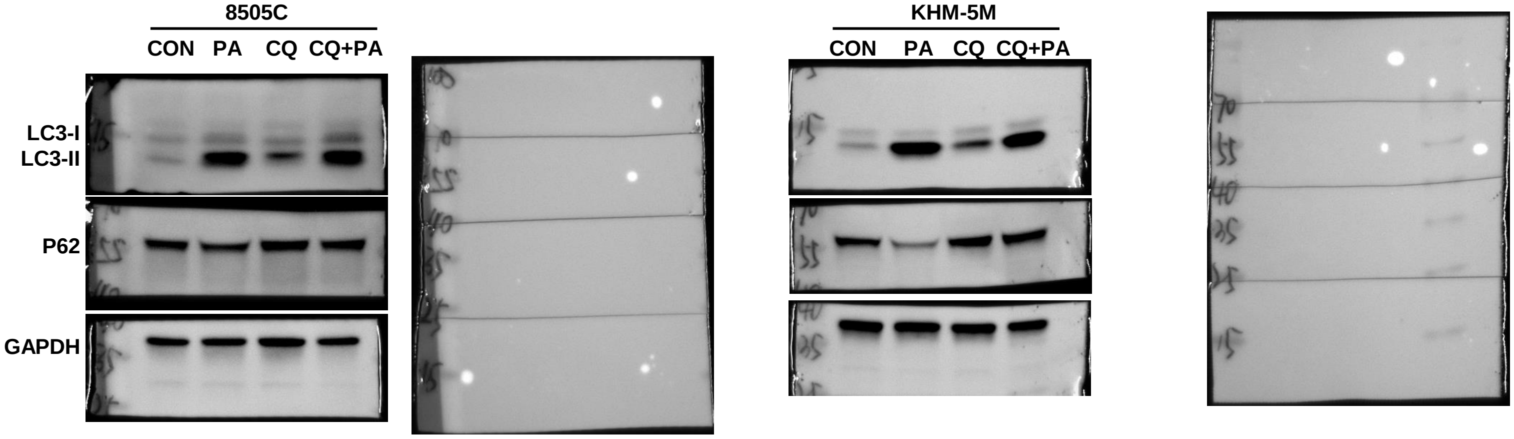

Original Western Blots for Figure 4F

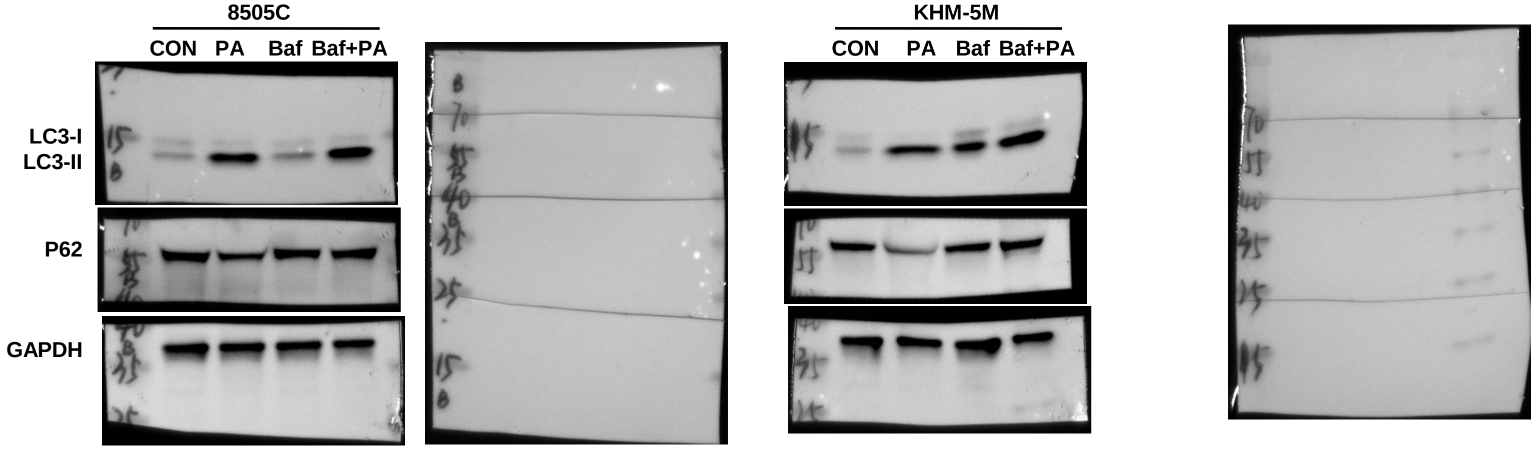

Original Western Blots for Figure 6D

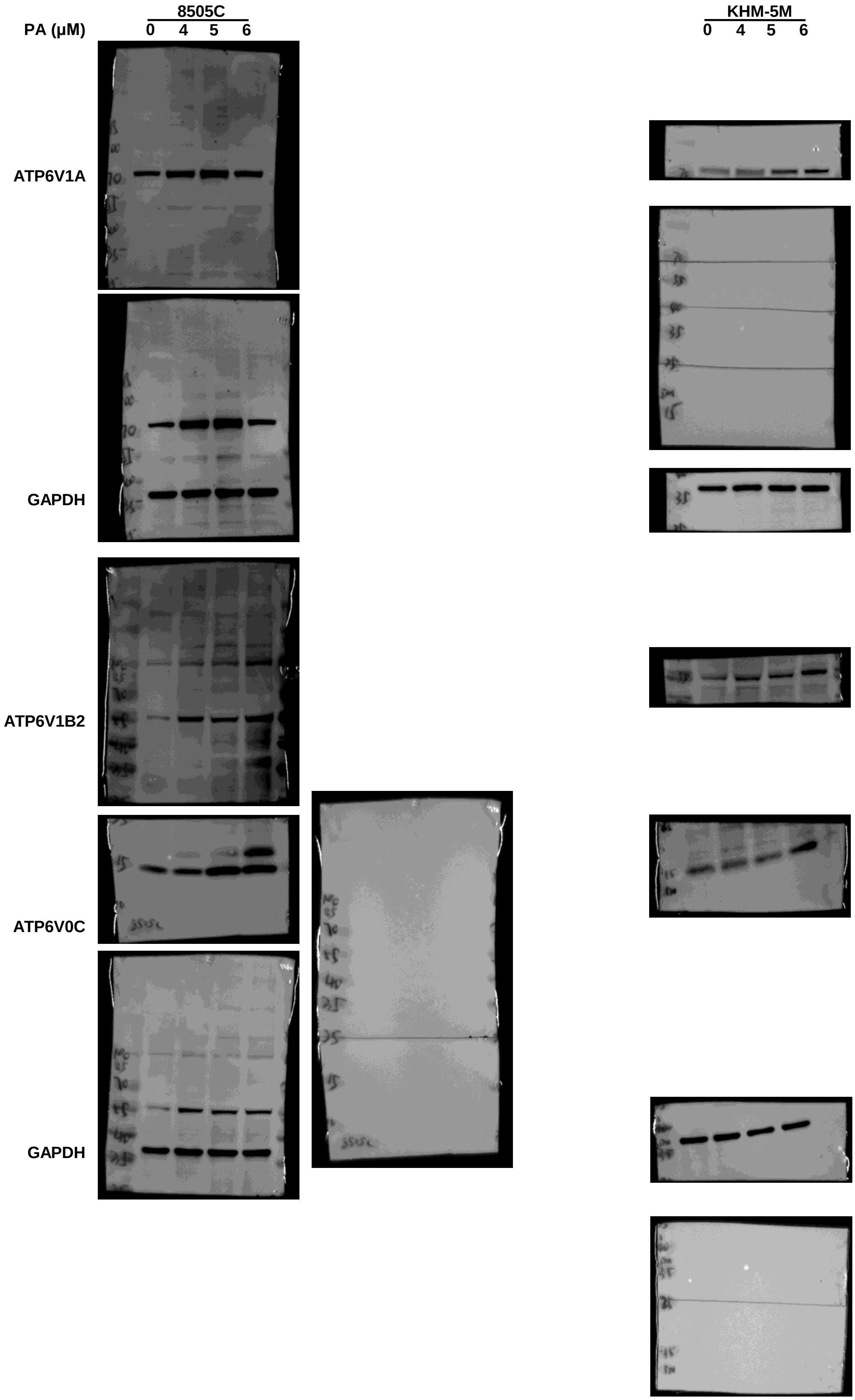

Original Western Blots for Figure 6I

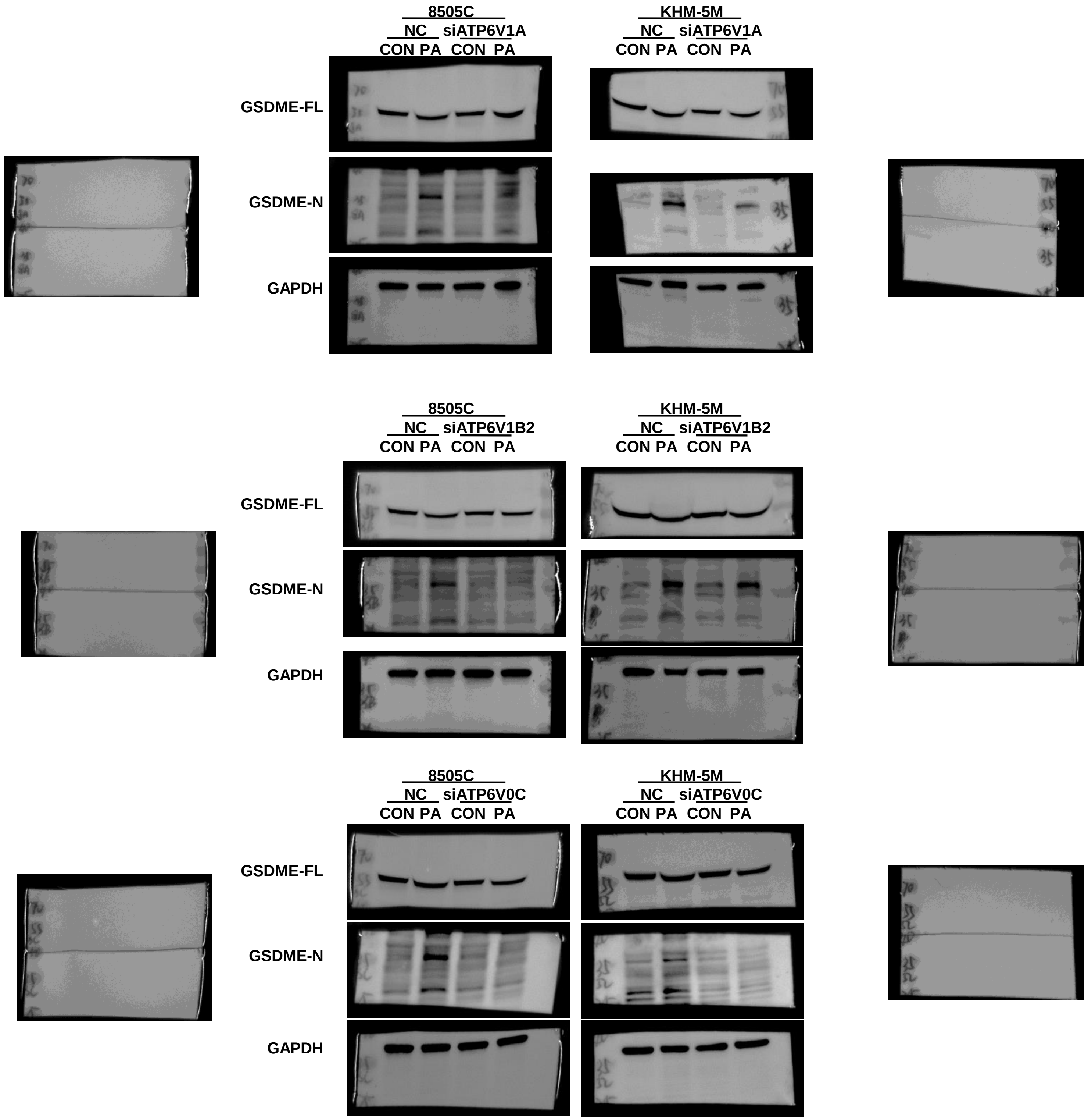

Original Western Blots for Figure S3C

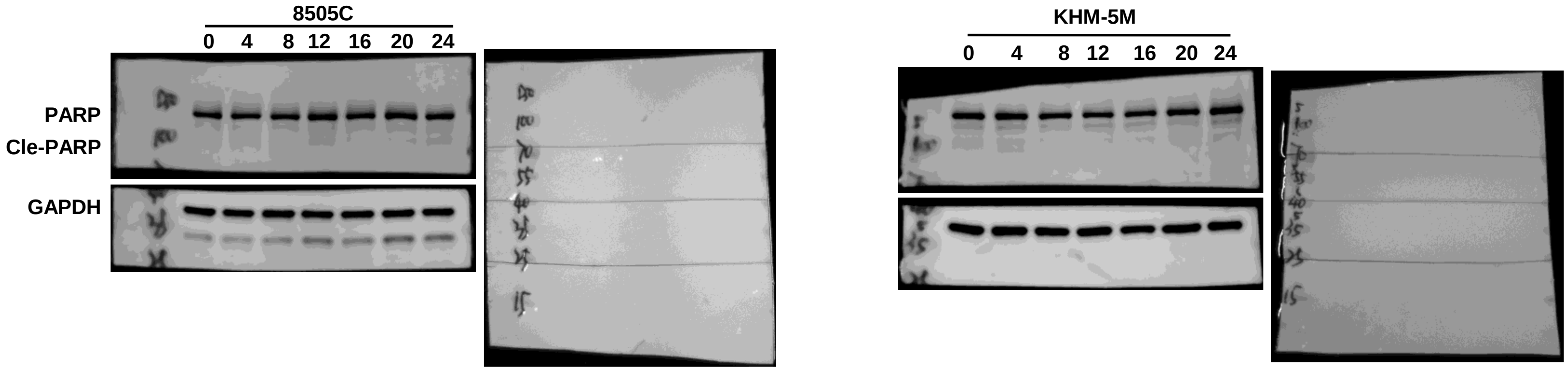

Original Western Blots for Figure S7A

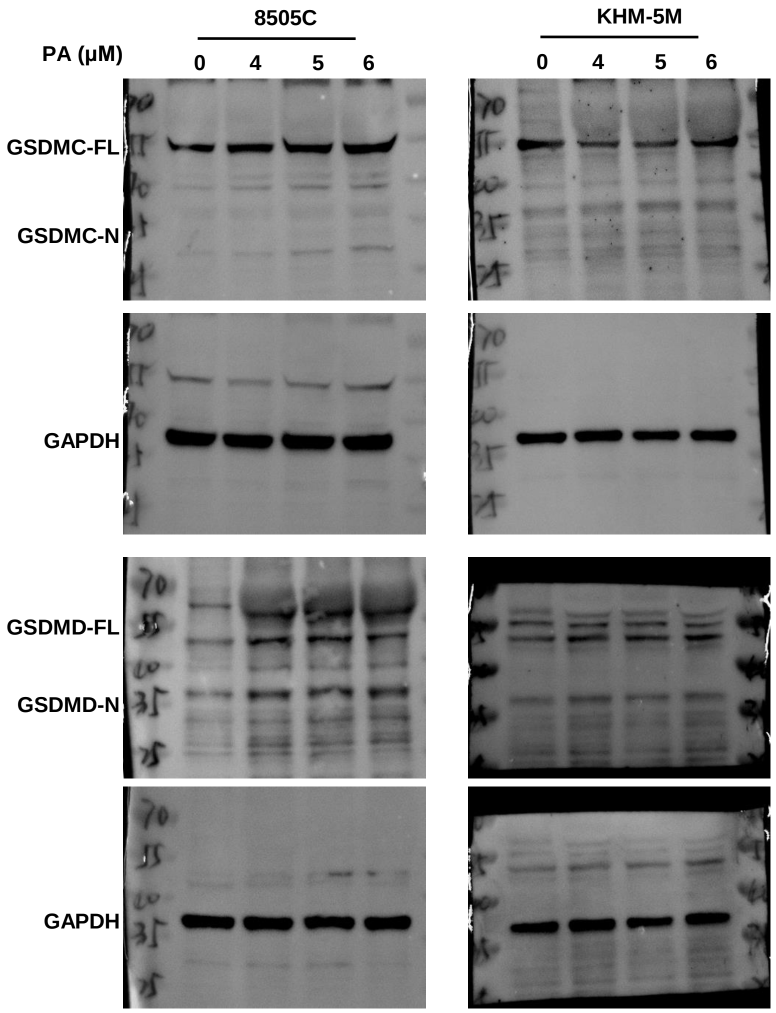

Original Western Blots for Figure S7B

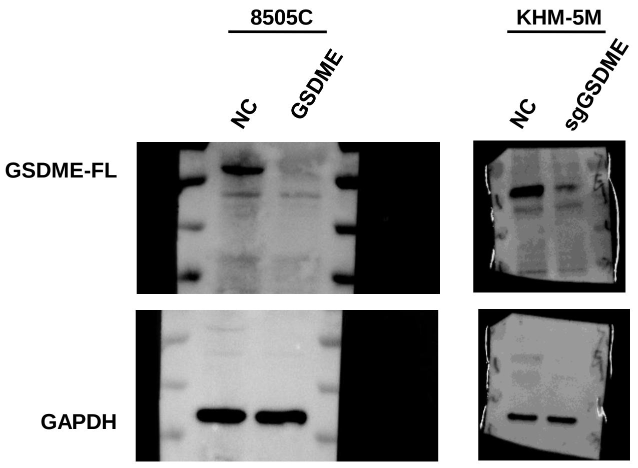

Original Western Blots for Figure S7E

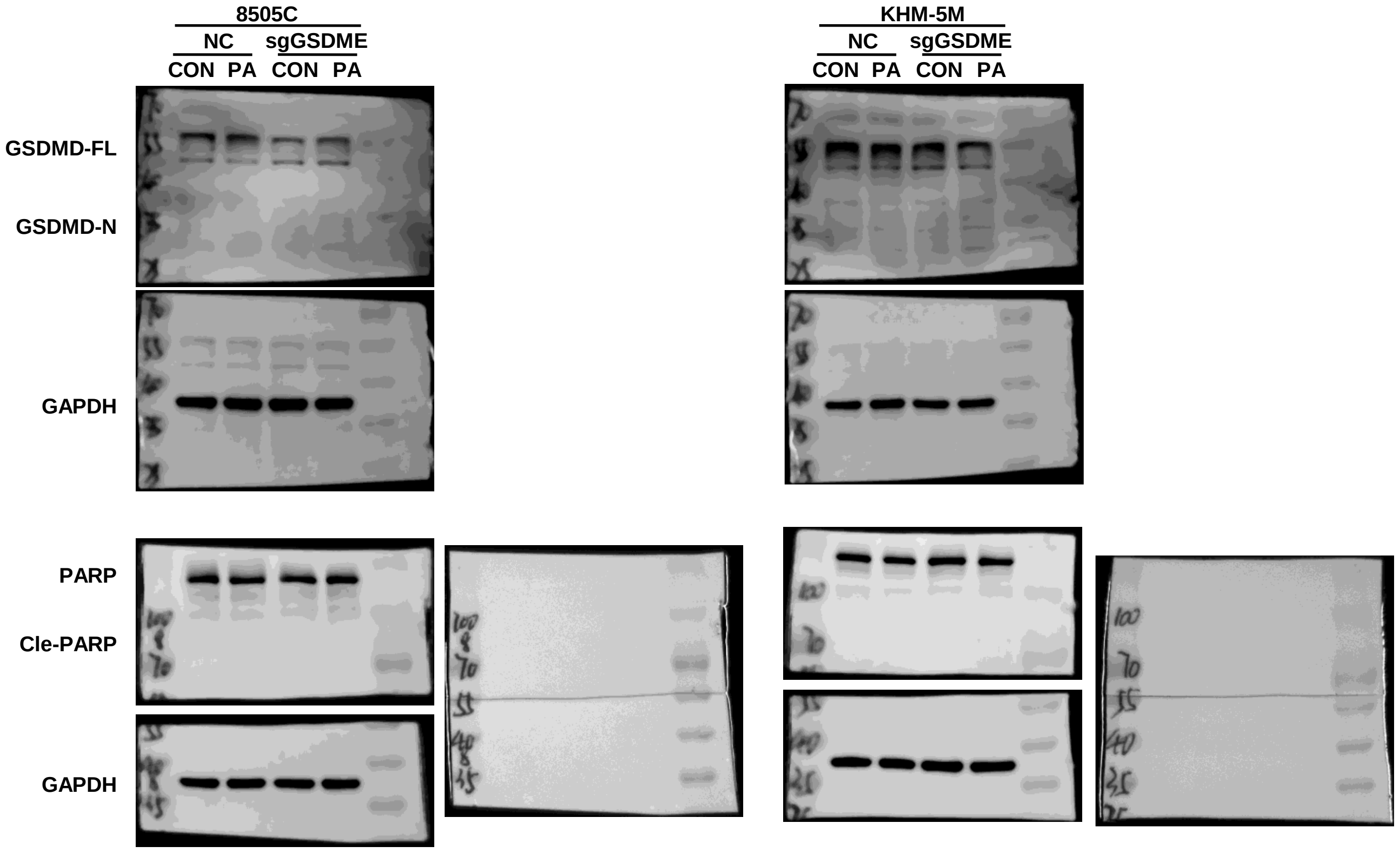

Original Western Blots for Figure S8A

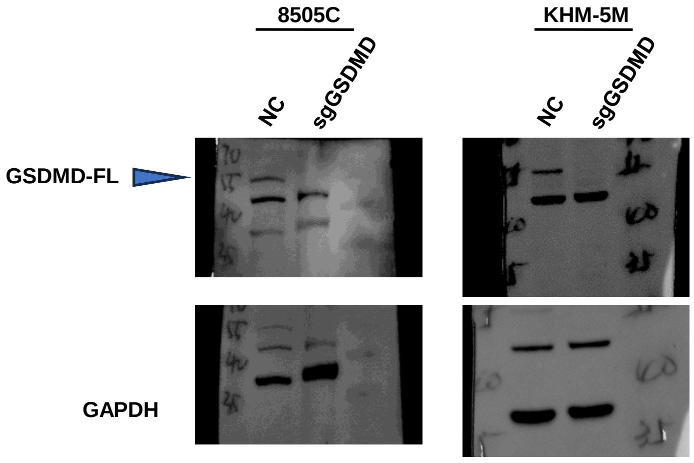

Original Western Blots for Figure S9A

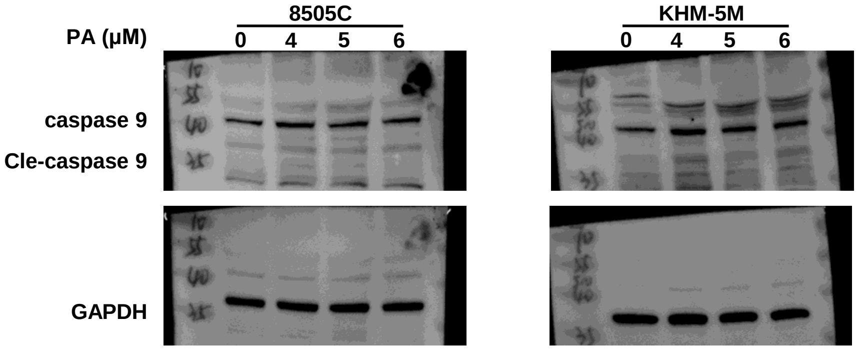

Original Western Blots for Figure S9B

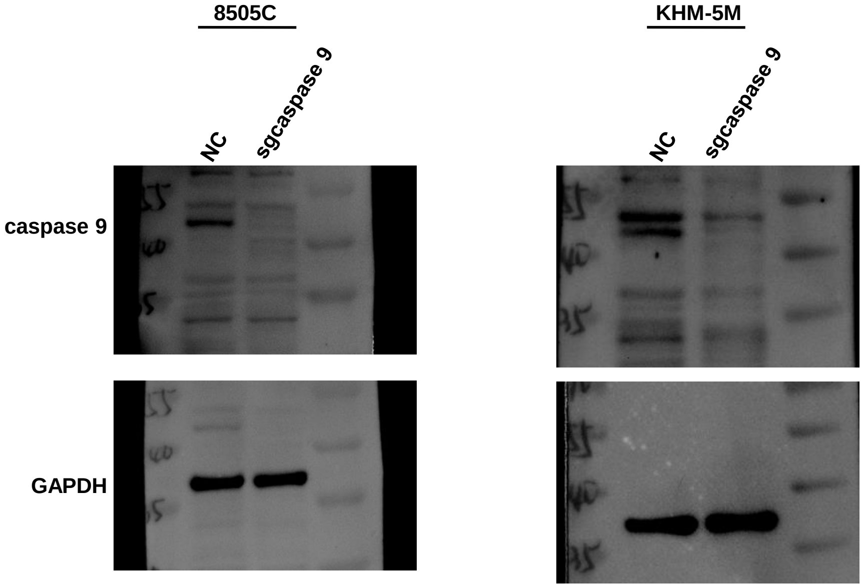

Original Western Blots for Figure S11A

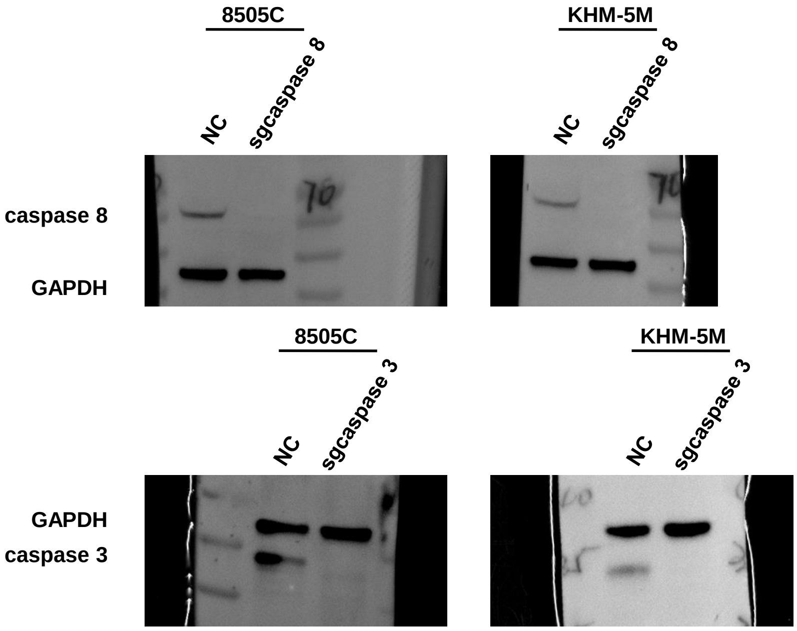

Original Western Blots for Figure S11C

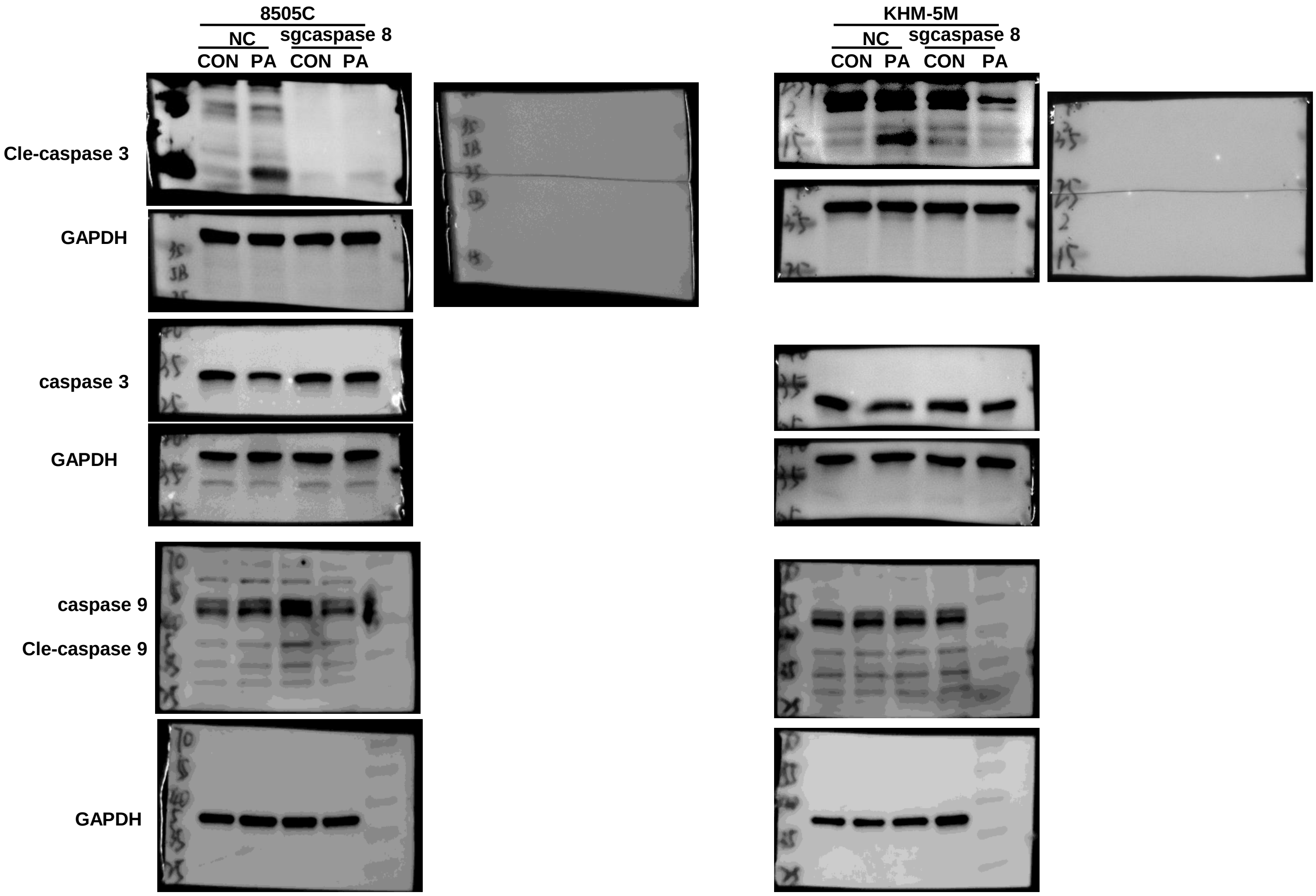

Original Western Blots for Figure S12B

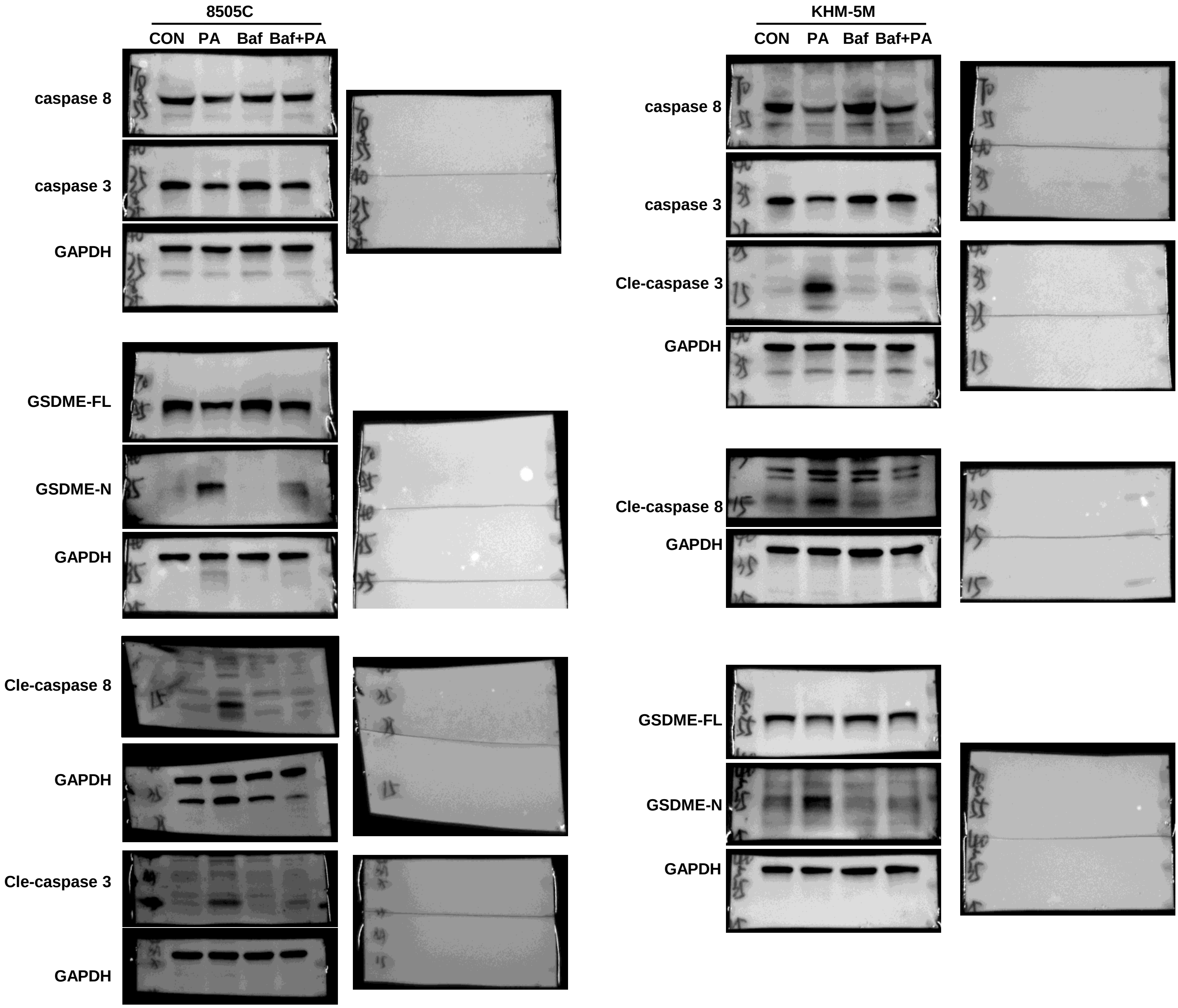

Original Western Blots for Figure S12B

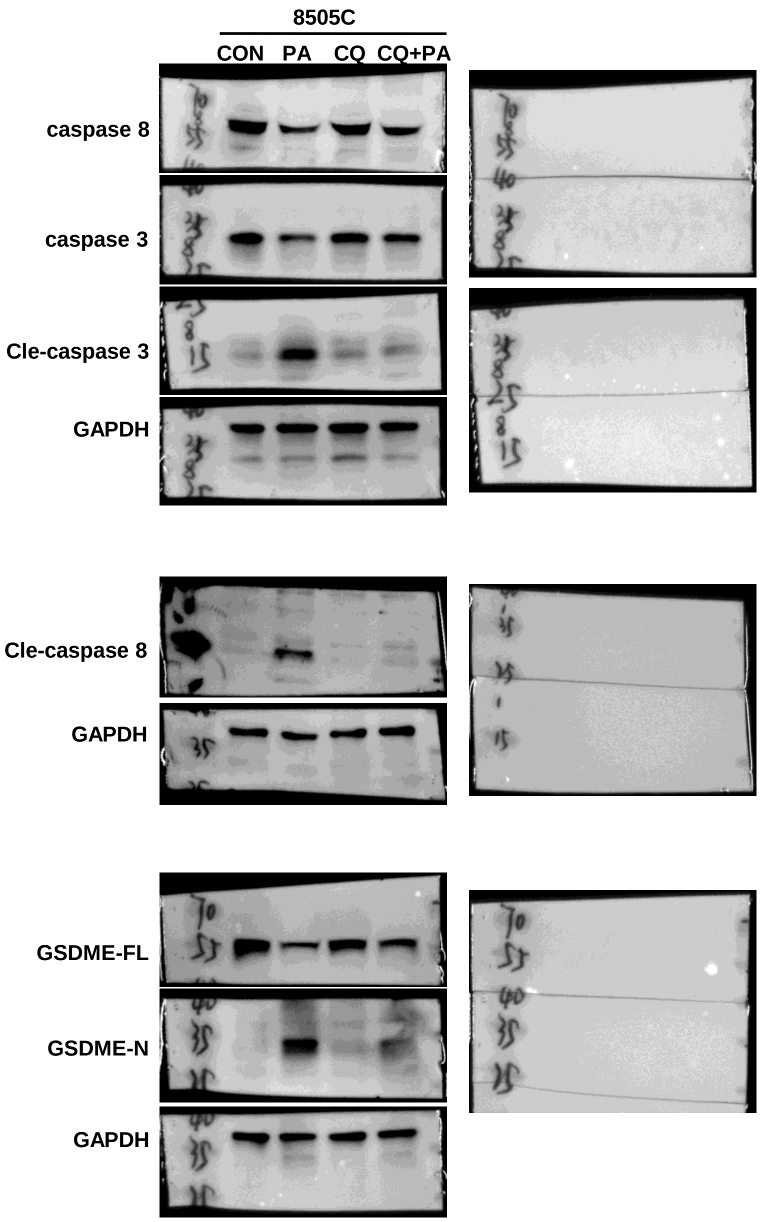

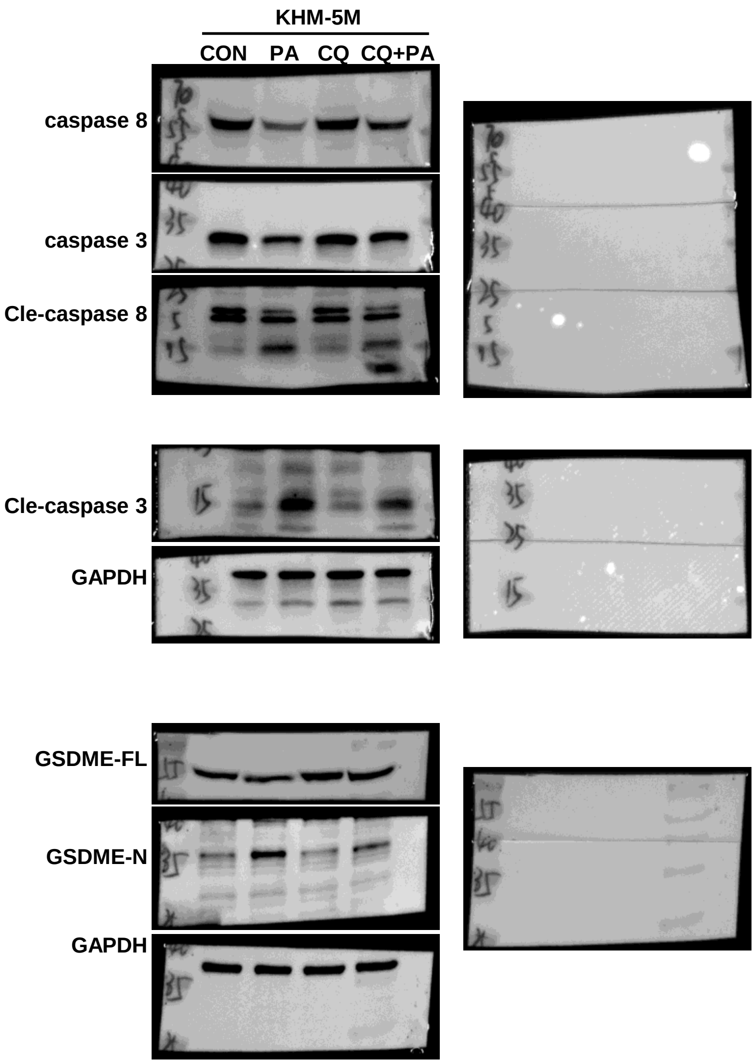

Original Western Blots for Figure S14A

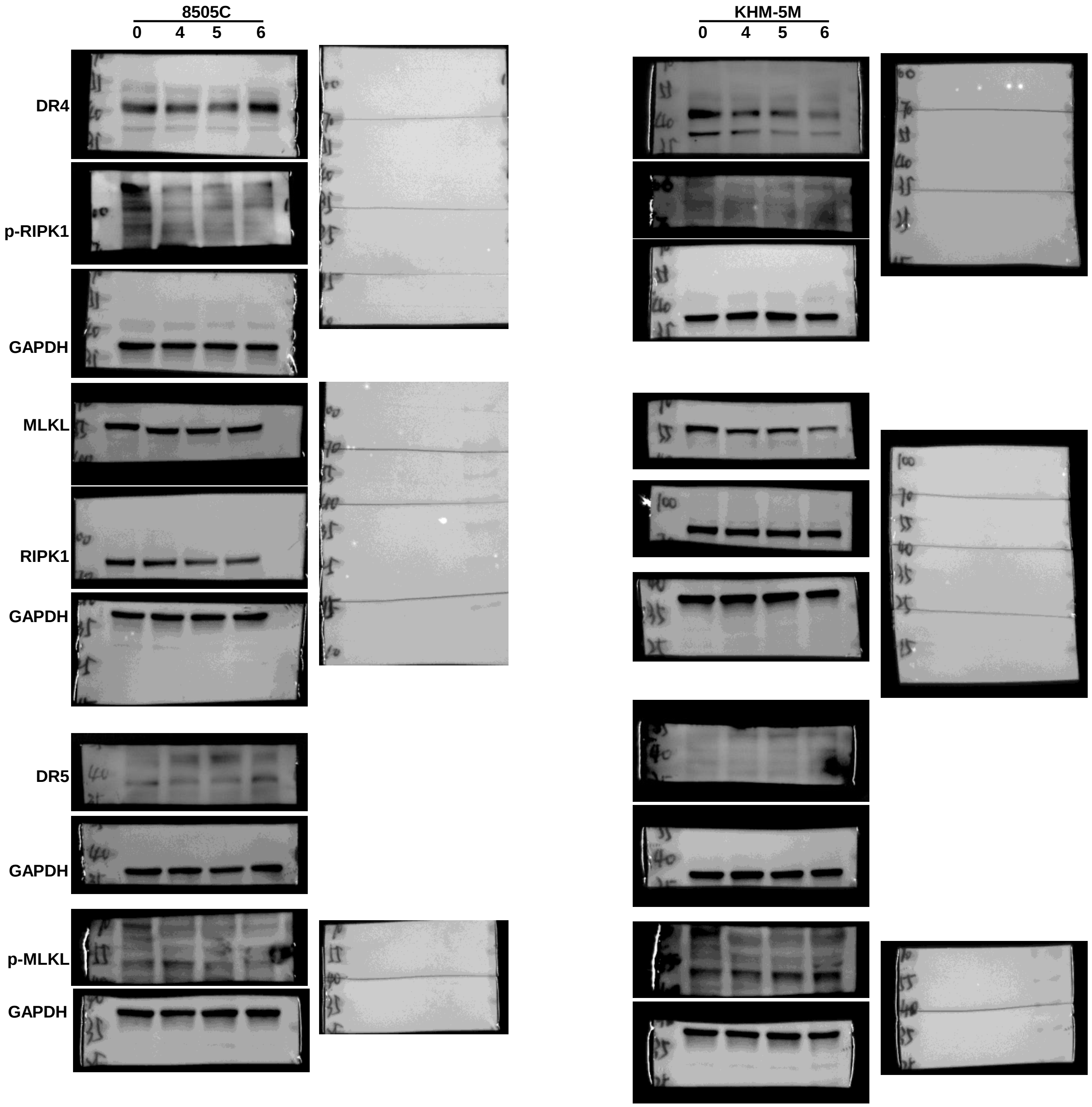

Original Western Blots for Figure S14B

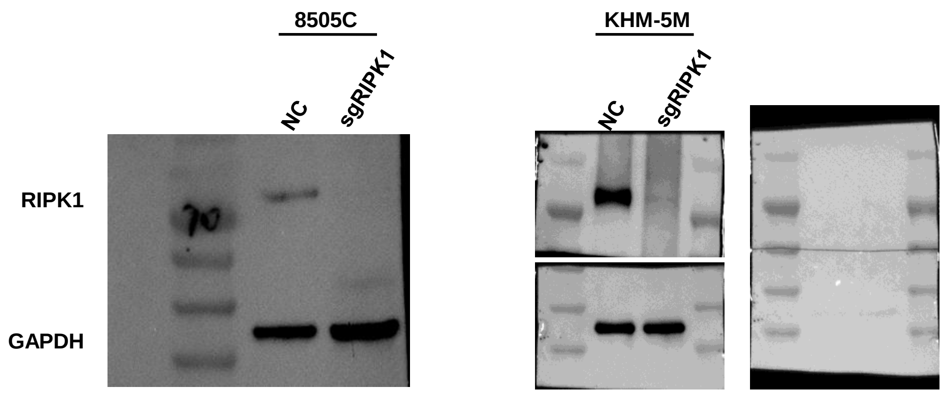

Original Western Blots for Figure S15B

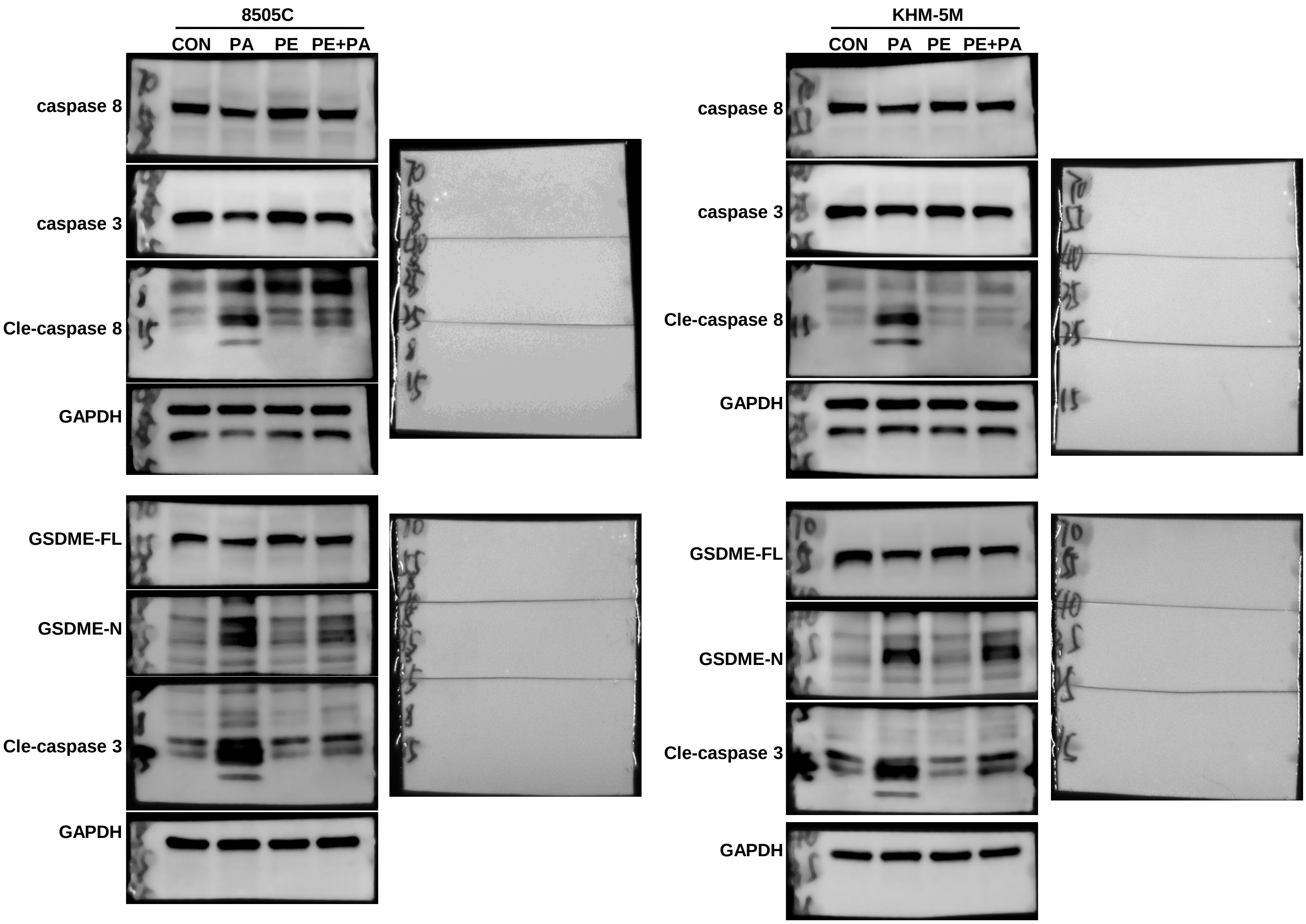

Original Western Blots for Figure S15B

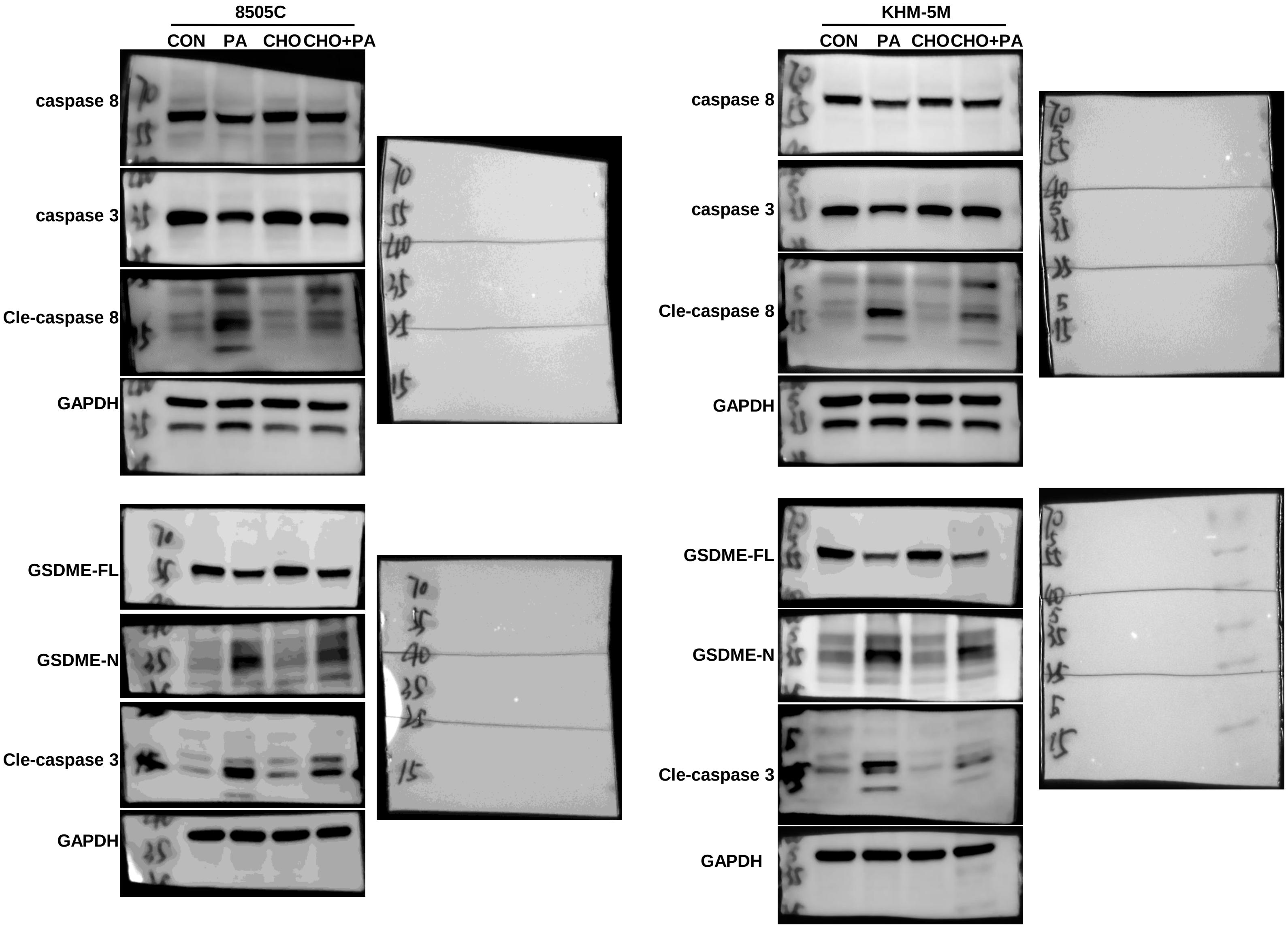

Original Western Blots for Figure S16D

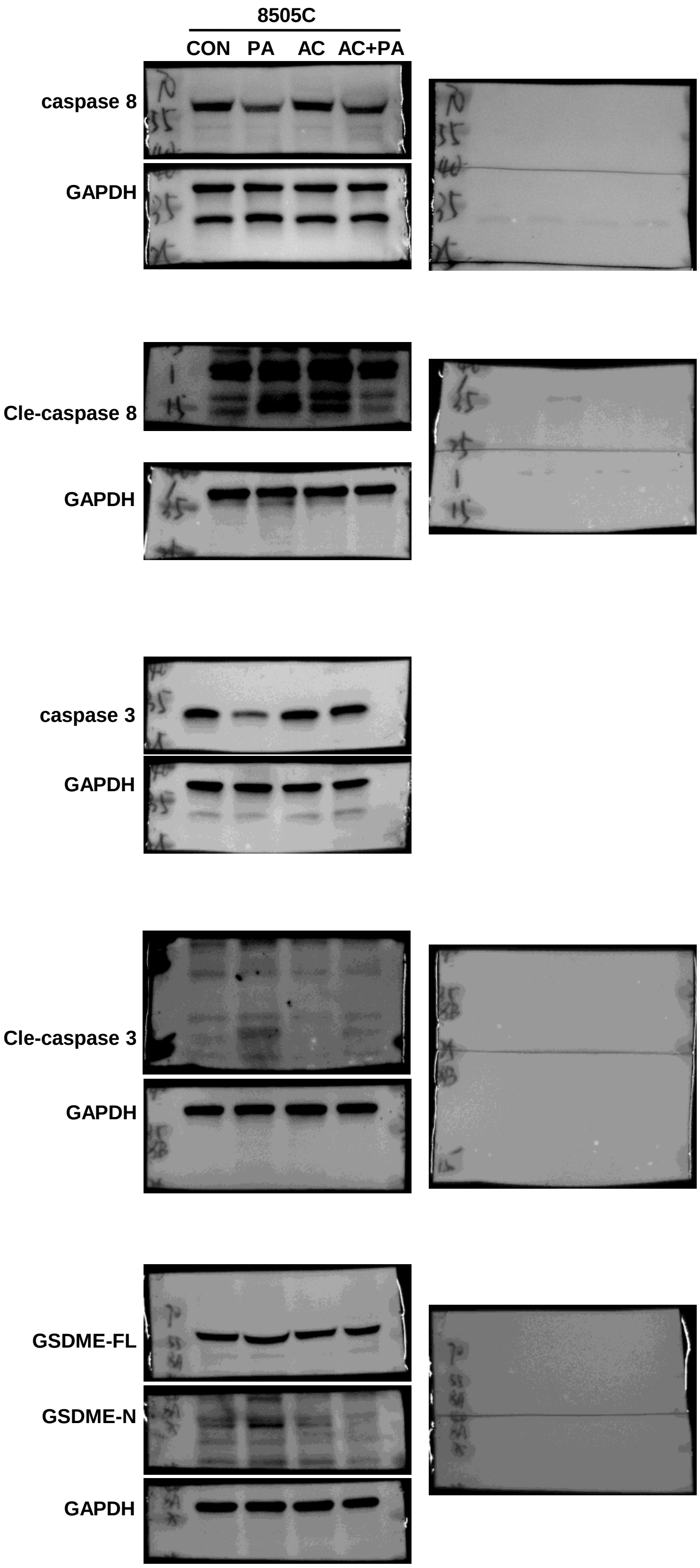

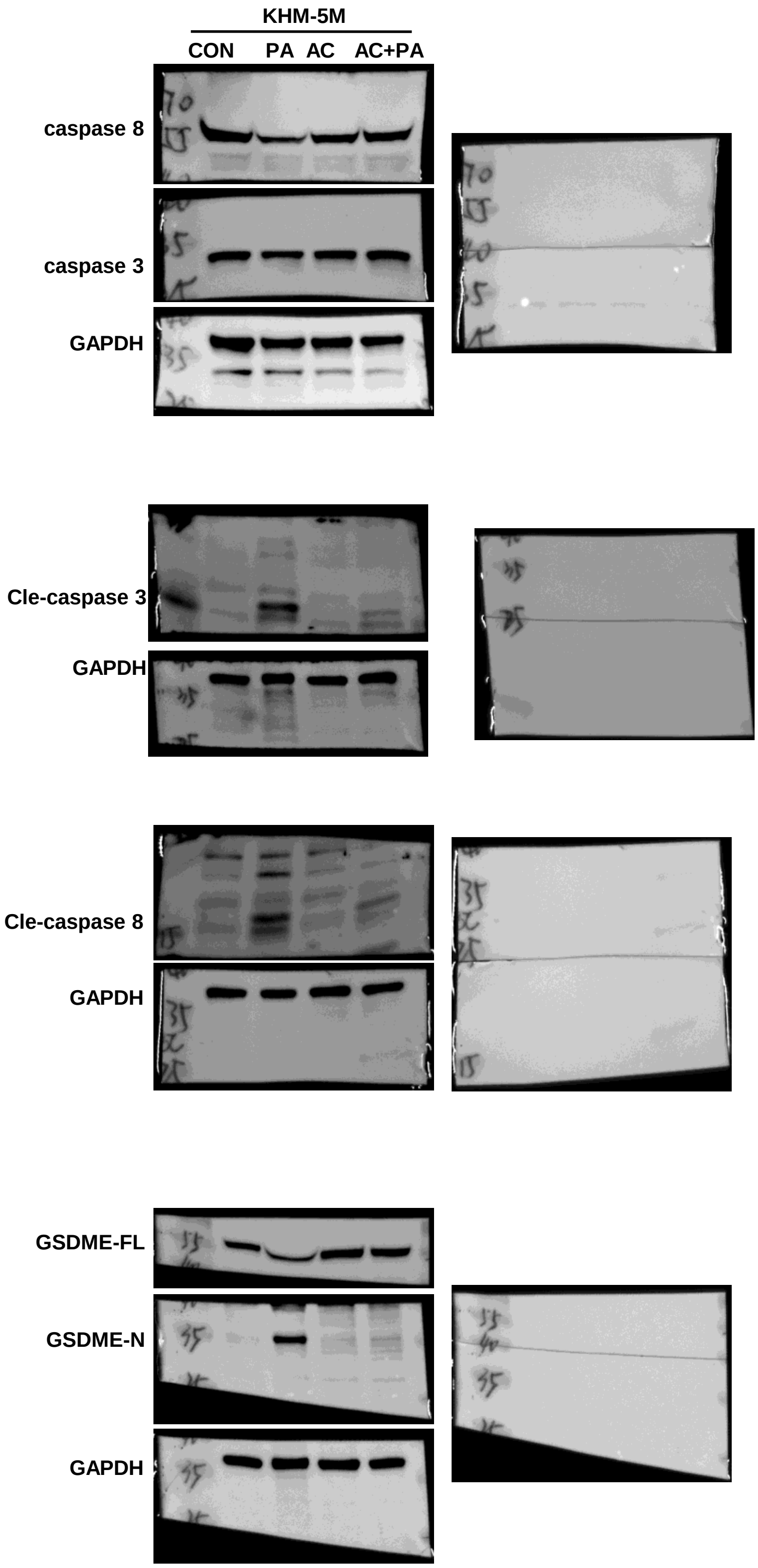

Original Western Blots for Figure S17B

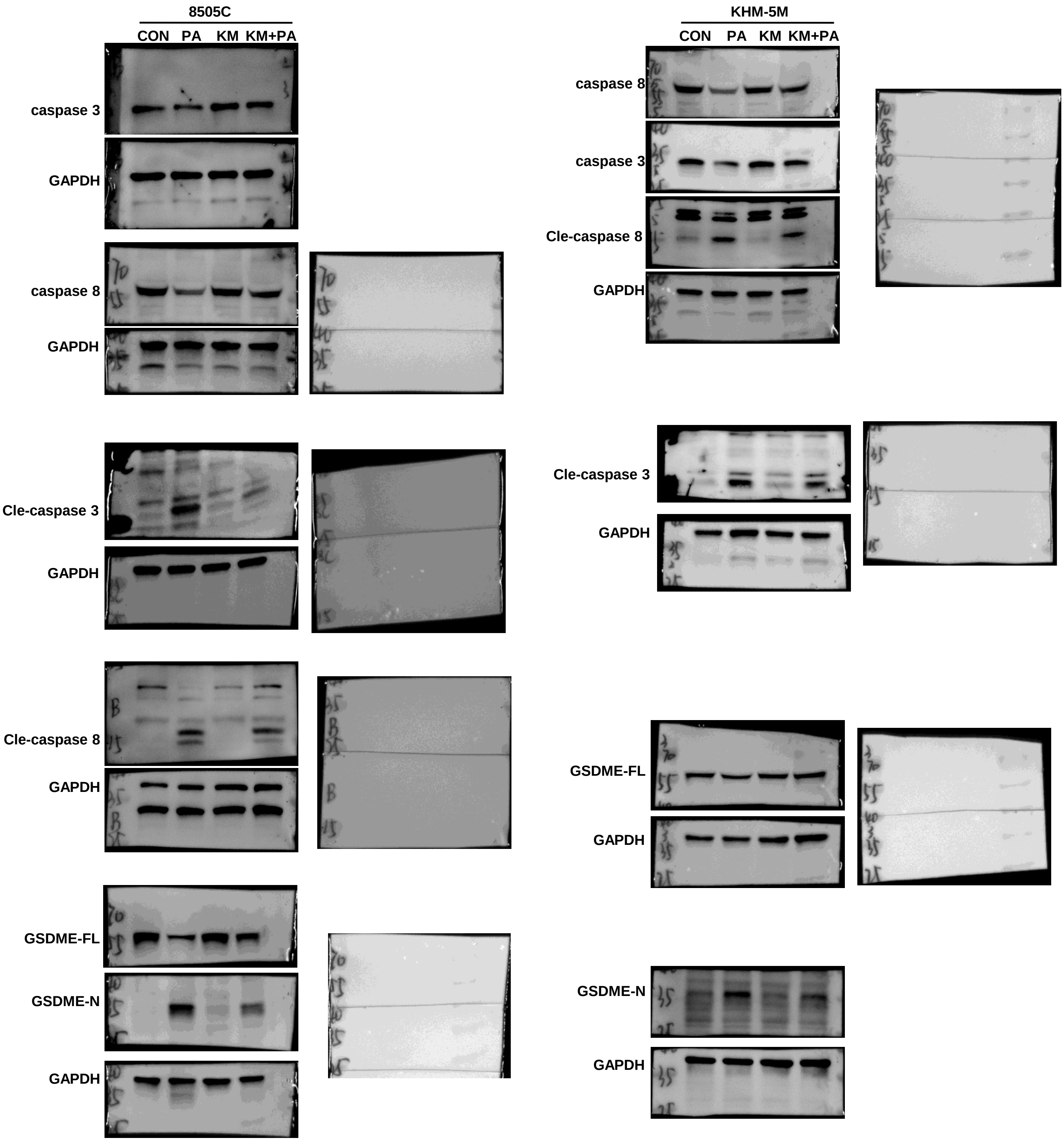

Original Western Blots for Figure S18B

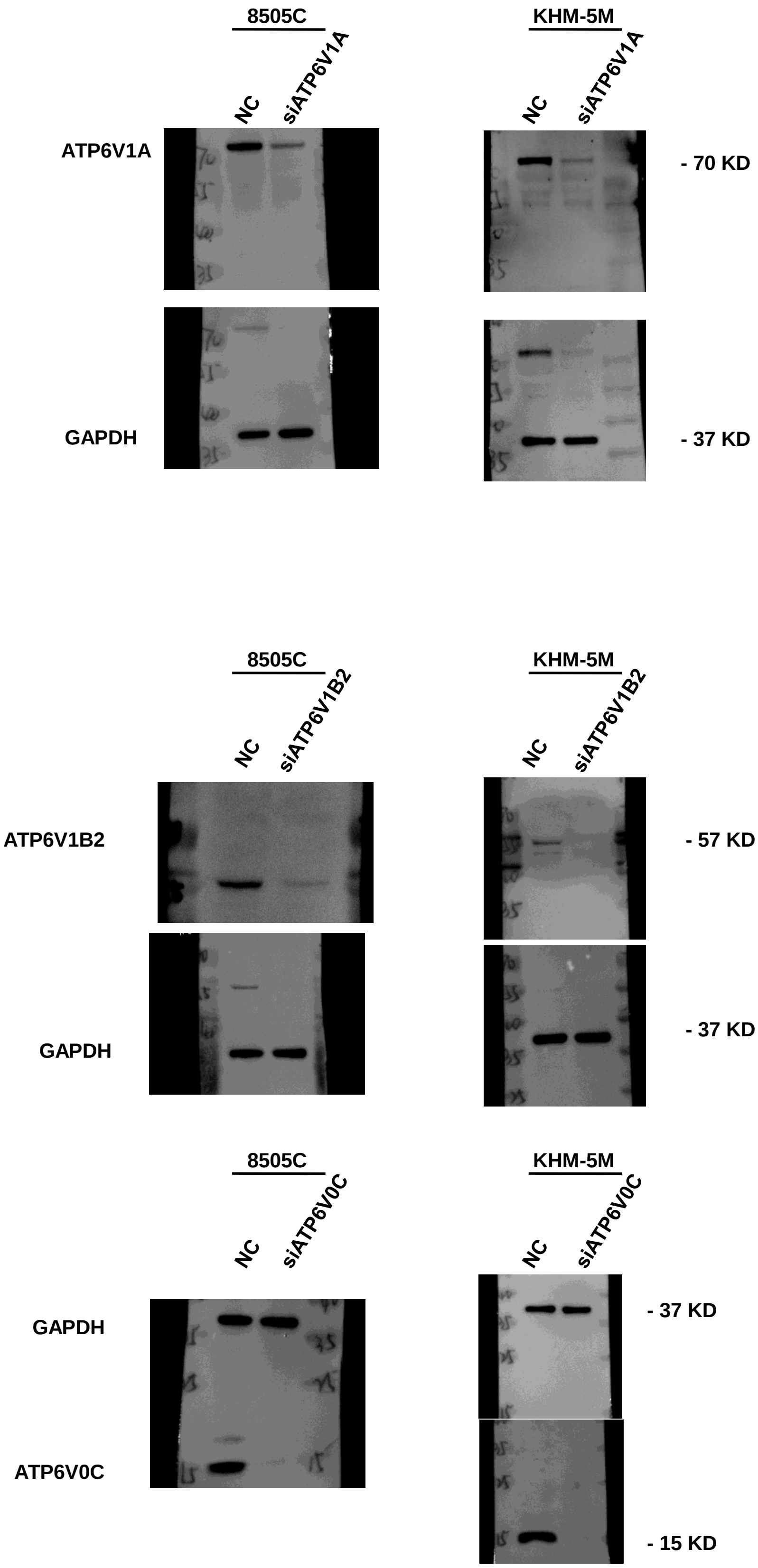

Original Western Blots for Figure S19A

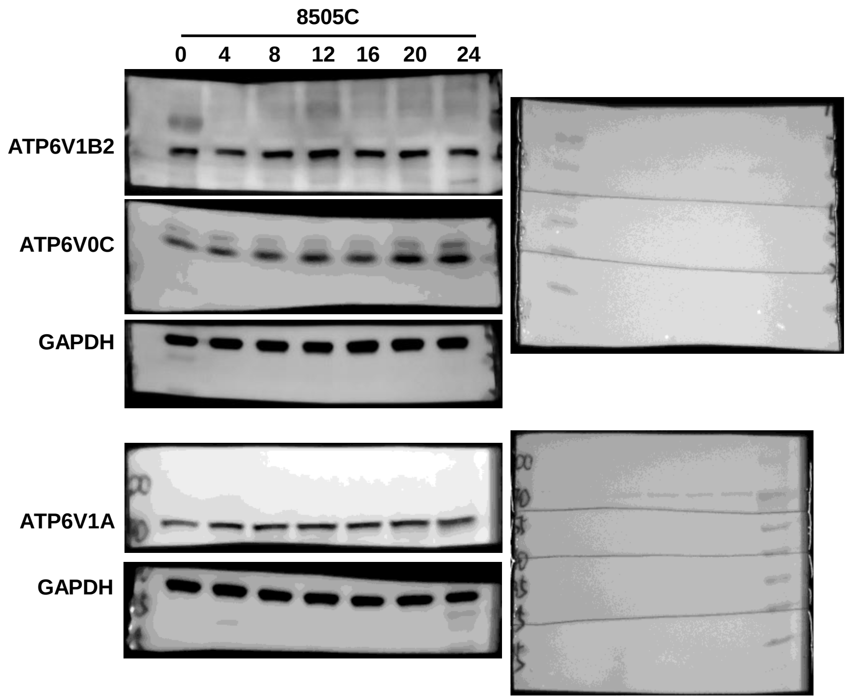

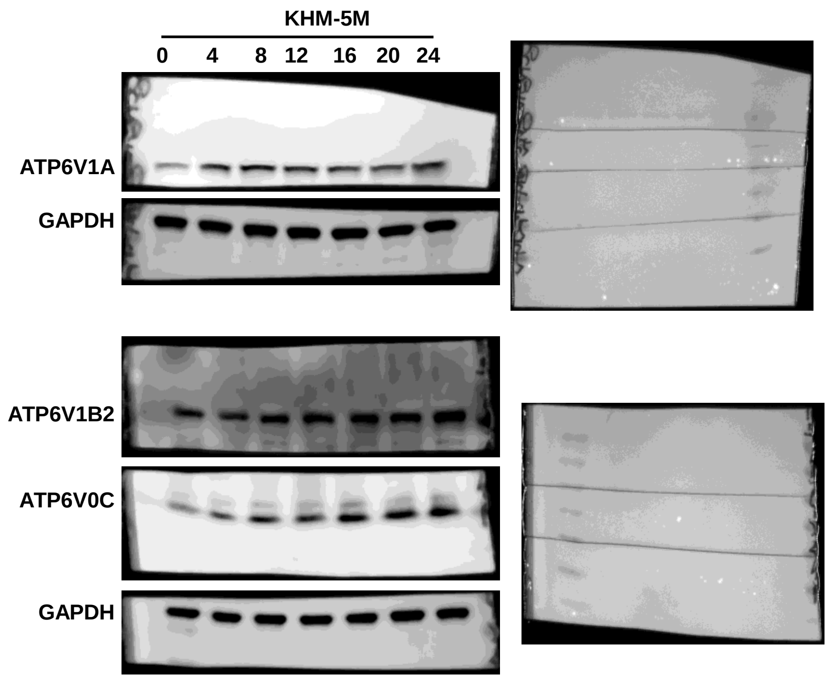

Original Western Blots for Figure S19C

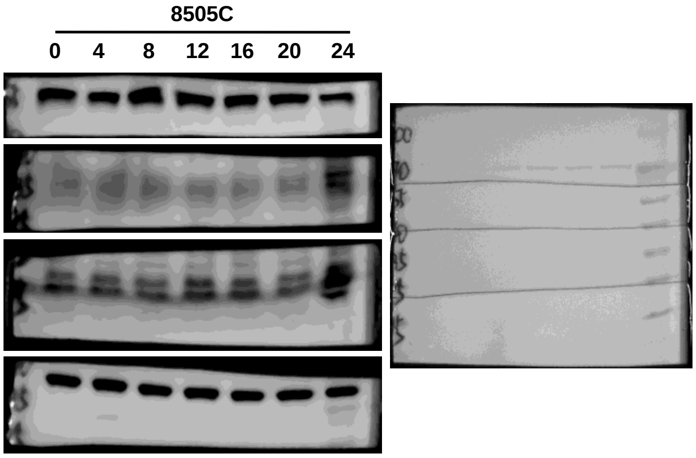

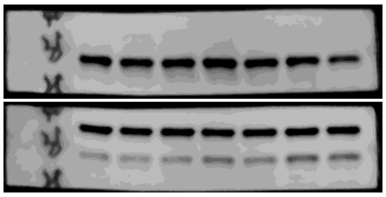

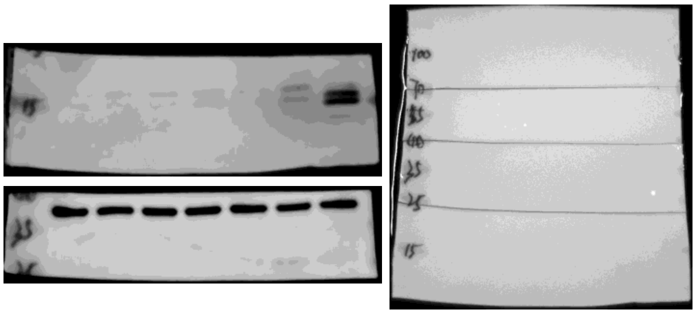

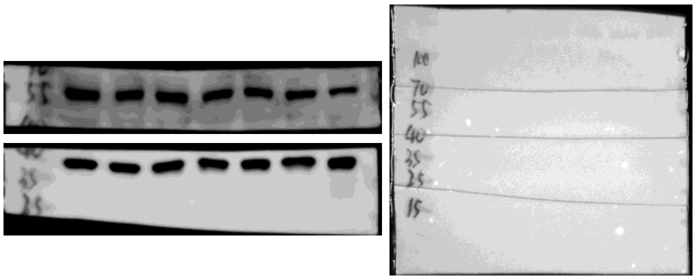

Original Western Blots for Figure S19C

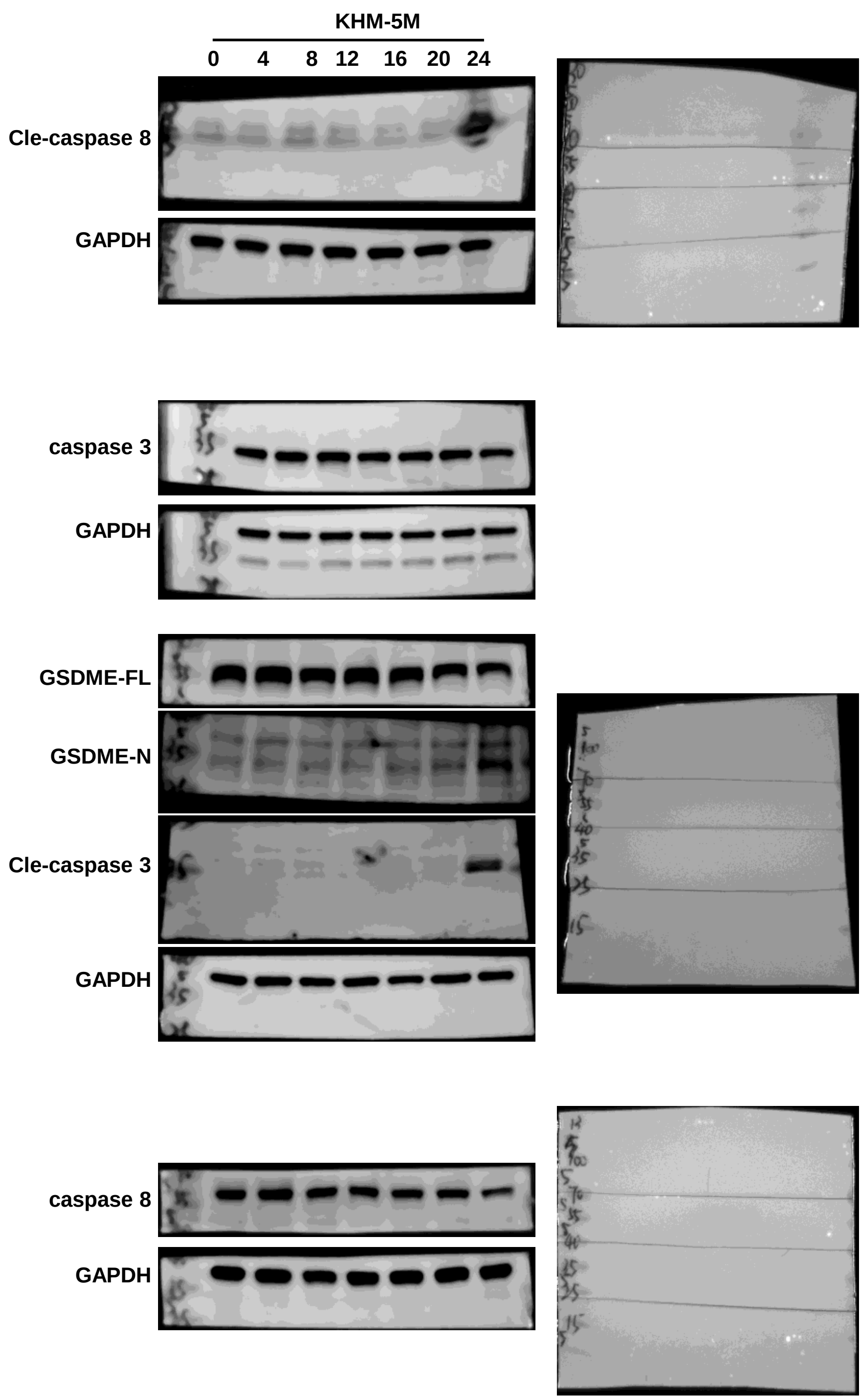

Original Western Blots for Figure S19D

8505C

0 4 8 12 16 20 24

LC3-I
LC3-II

GAPDH

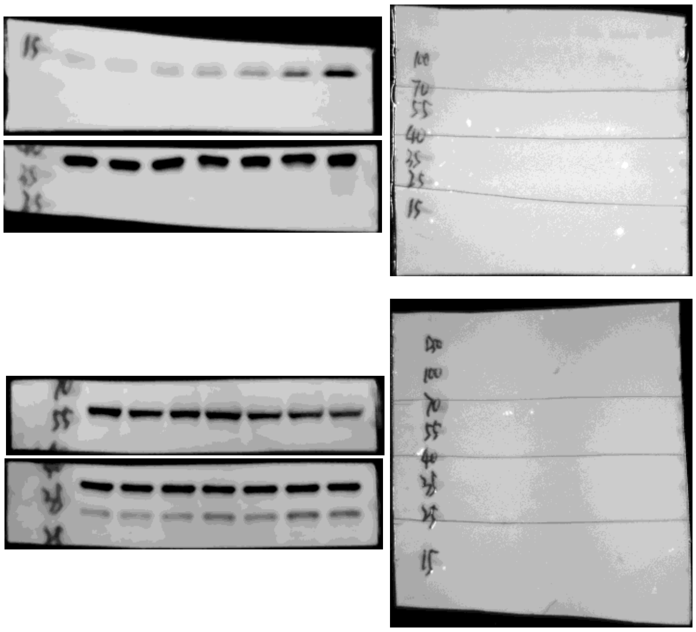

KHM-5M

0 4 8 12 16 20 24

LC3-I
LC3-II

GAPDH

P62

GAPDH

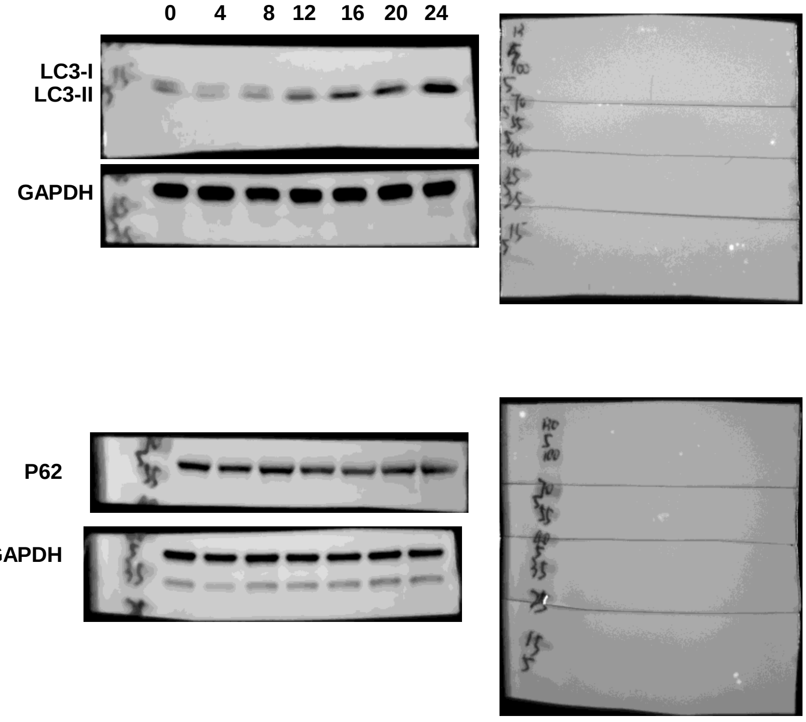

Supplement: Supplementary file 1 — Original western blots [file 41419_2024_6985_MOESM1_ESM.pdf]
