# Supplementary material for: Prosapogenin A induces GSDME-dependent pyroptosis of anaplastic thyroid cancer through vacuolar ATPase activation-mediated lysosomal over-acidification
Source: Cell Death Dis. 2024 Aug 13;15(8):586. doi: 10.1038/s41419-024-06985-z (PMC11322489; doi:10.1038/s41419-024-06985-z)
Supplement: Supplementary file 2 — supplement figure [file 41419_2024_6985_MOESM2_ESM.docx]

**Supplementary Materials for**

Prosapogenin A induces GSDME-dependent pyroptosis of anaplastic thyroid cancer through vacuolar ATPase activation-mediated lysosomal over-acidification

Yunye Liu†, Yawen Guo†, Qian Zeng, Yiqun Hu, Ru He, Wenli M, Chenhong Qian, Tebo Hua, Fahuan Song, Yefeng Cai, Lei Zhu, Xinxin Ren, Jiajie Xu, Chuanming Zheng, Lingling Ding, Jingyan Ge, Wenzhen Wang, Haifeng Xu, Minghua Ge*, Guowan Zheng*

*Corresponding authors.

E-mail address: geminghua@hmc.edu.cn, guowan.zheng@ki.se


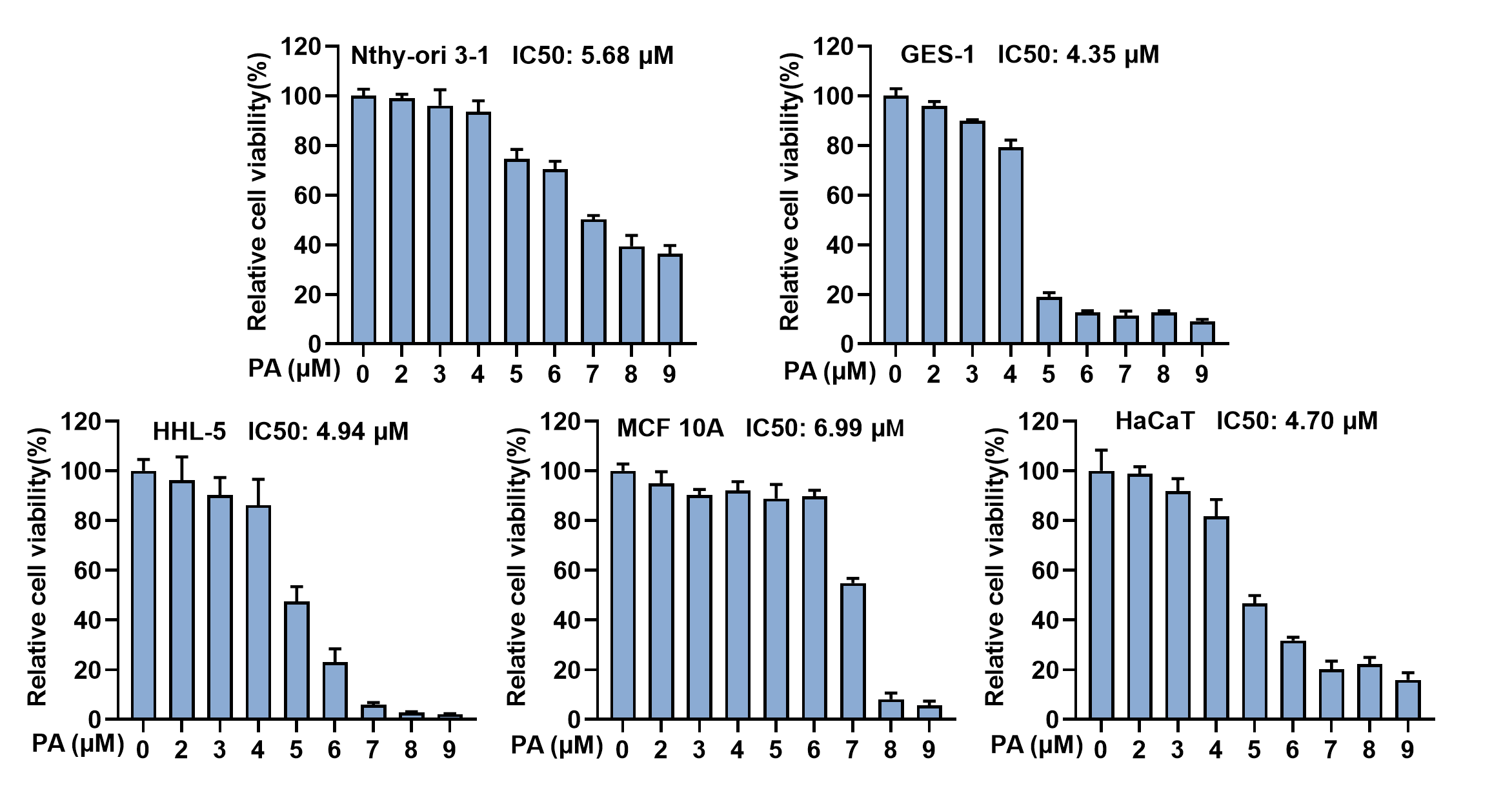


**Figure S1 Effects of PA on immortalized non-malignant cells.** Nthy-ori 3-1, GES-1, HHL-5, MCF 10A and HaCaT cells were treated with 0, 2, 3, 4, 5, 6, 7, 8 and 9 μM PA for 24 h, and relative cell viability was assessed by CCK8.

**
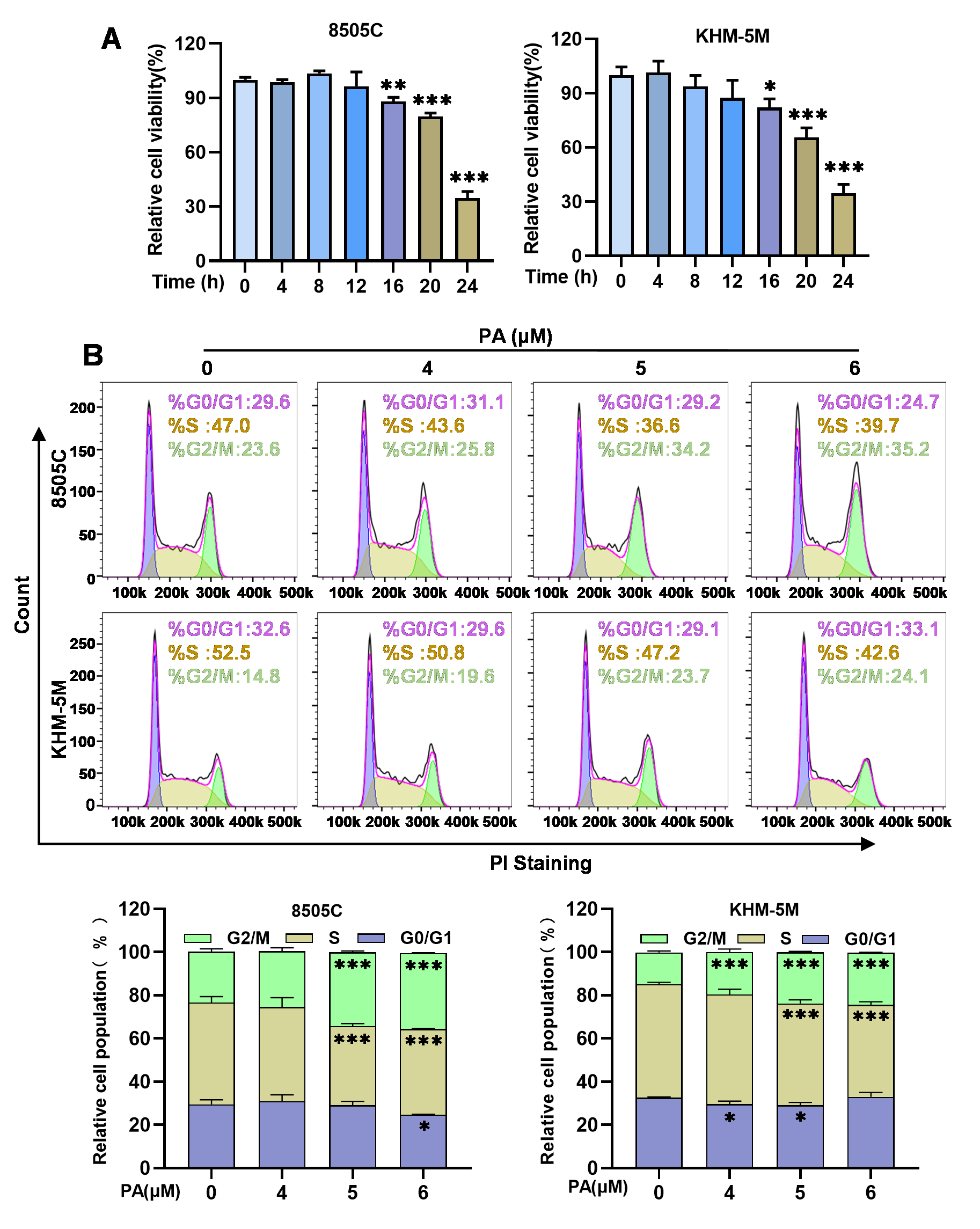
**

**Figure S2 PA increases the proportion of G2/M phase and cell death in ATC cells. (A)** After PA (5 μM) treated for 0, 4, 8, 12, 16, 20, 24 h in 8505C and KHM-5M cells, the cell viabilities were measured by the CCK-8 assay. **(B)** After 8505C and KHM-5M cells were treated with PA for 8 h, at indicated concentrations (0, 4, 5 and 6 μM), the proportions of cell populations at each cell cycle phase relative to total phases were indicated. Data are shown as mean ± SD for n = 3 (biological replicates). **p* < 0.05, ***p* < 0.01, ****p* < 0.001.

**
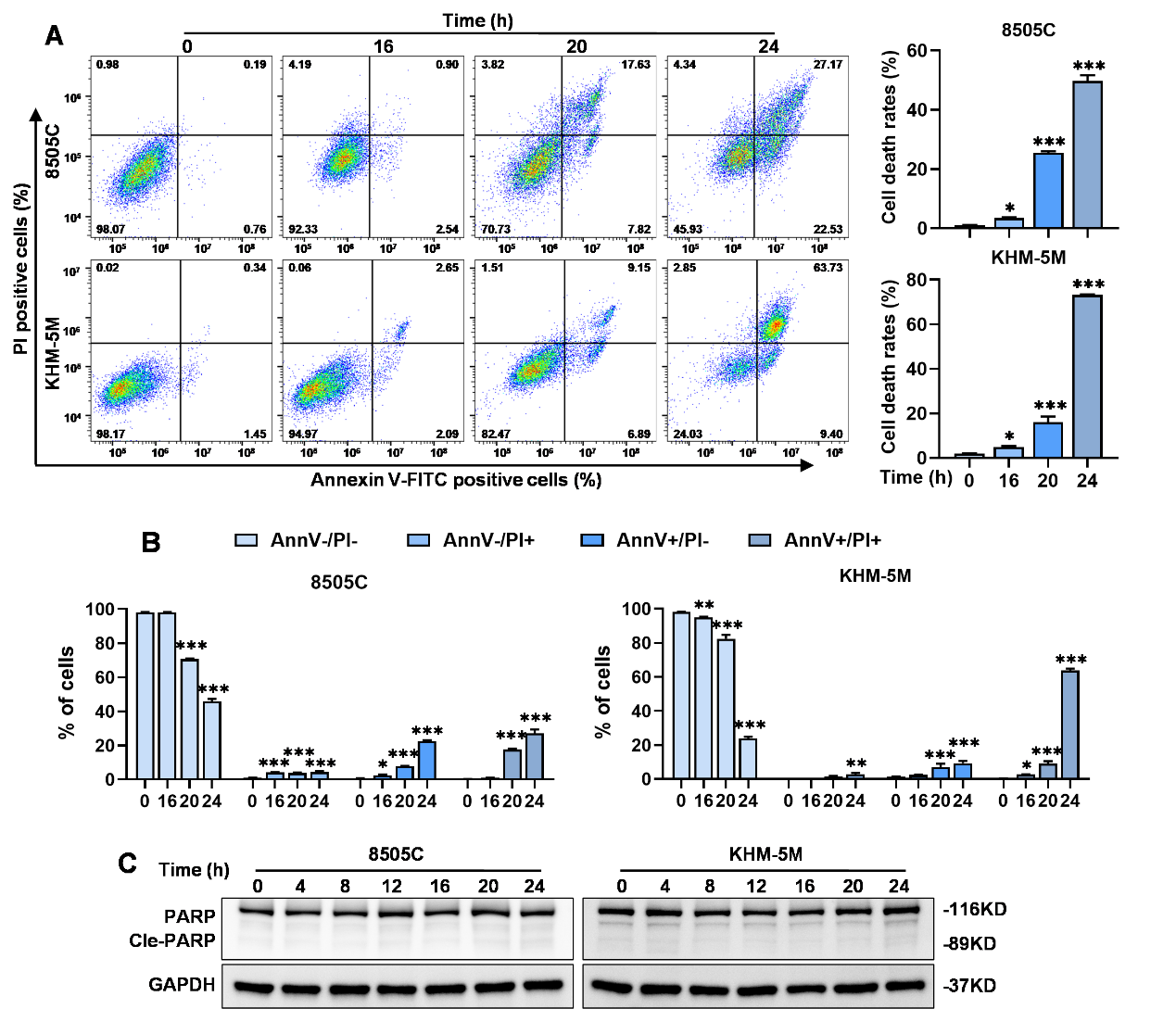
**

**Figure S3 PA induced ATC cell death in a time-dependent manner. (A)** 8505C and KHM-5M cell death were measured by flow cytometry after PA (5 μM) treatment for 0, 16, 20, 24 h, data was analyzed using GraphPad prism, and **(B)** percentage of AnnV^-^/PI^-^, AnnV^-^/PI^+^, AnnV^+^/PI^-^ and AnnV^+^/PI^+^ were indicated. **(C)** After PA (5 μM) treated for 0, 4, 8, 12, 16, 20, 24 h in 8505C and KHM-5M cells, PARP, cleaved-PARP and GAPDH protein levels were detected by western blot. Data are shown as mean ± SD for n = 3 (biological replicates). **p* < 0.05, ***p* < 0.01, ****p* < 0.001.

**
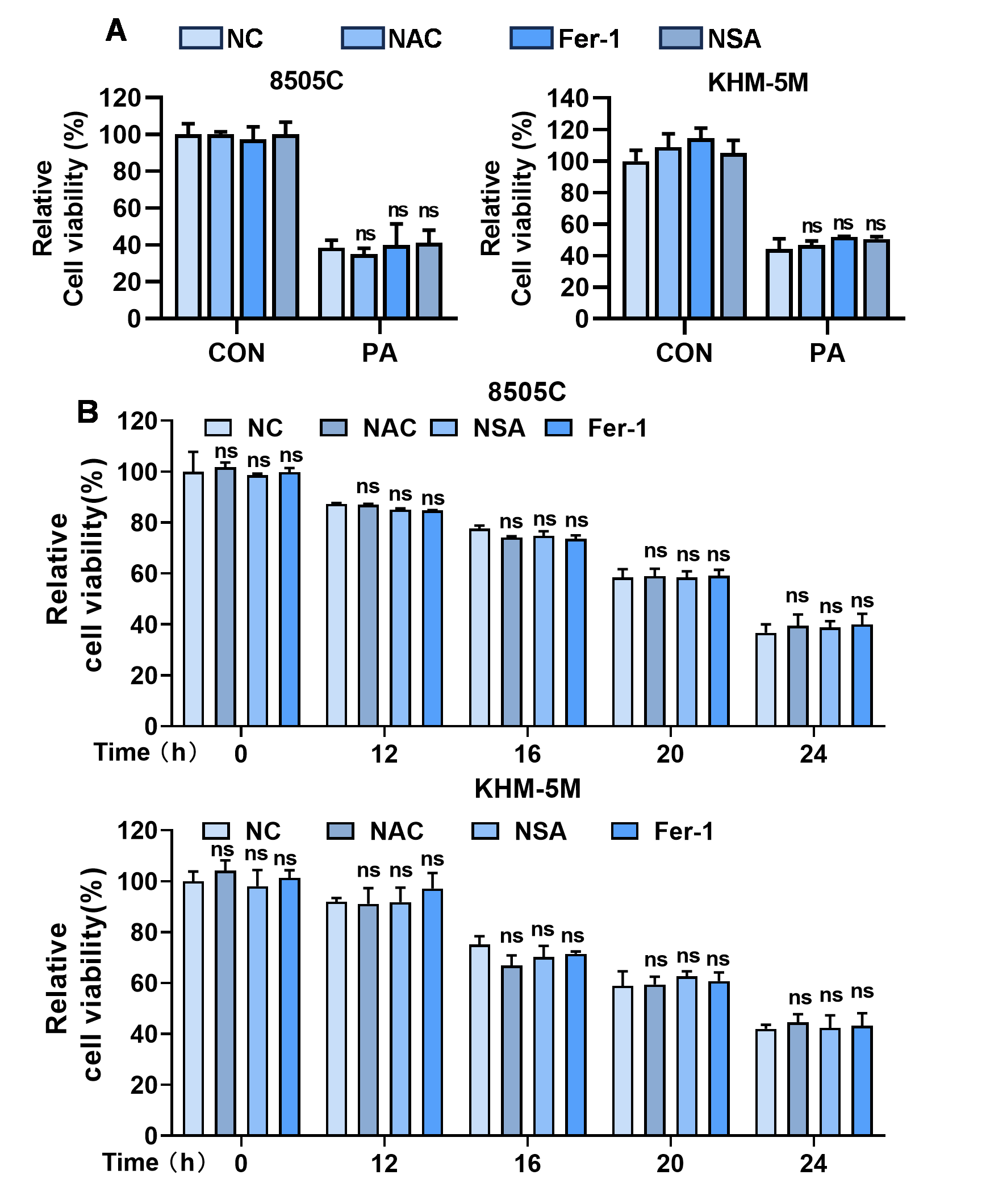
**

**Figure S4 ROS scavenger, Ferroptosis, and necroptosis inhibitor hardly affects cell death induced by PA in ATC cells. (A)** PA (5 μM) is used in combination with an ROS scavenger NAC (2.5 mM, pre-treated for 2 h), a ferroptosis inhibitor Fer-1 (1 μM, pre-treated for 2 h) and a necroptosis inhibitor NSA (1 μM, pre-treated for 2 h) for 24 h, cell viability was measured by the CCK-8 assay. **(B)** PA (5 μM) is used in combination with NAC (2.5 mM, pre-treated for 2 h), NSA (1 μM, pre-treated for 2 h) and Fer-1 (1 μM, pre-treated for 2 h) for 0, 12, 16, 20, 24 h, cell viability was measured by the CCK-8 assay. Data are shown as mean ± SD for n = 3 (biological replicates). ns, *p* ＞ 0.05.

**
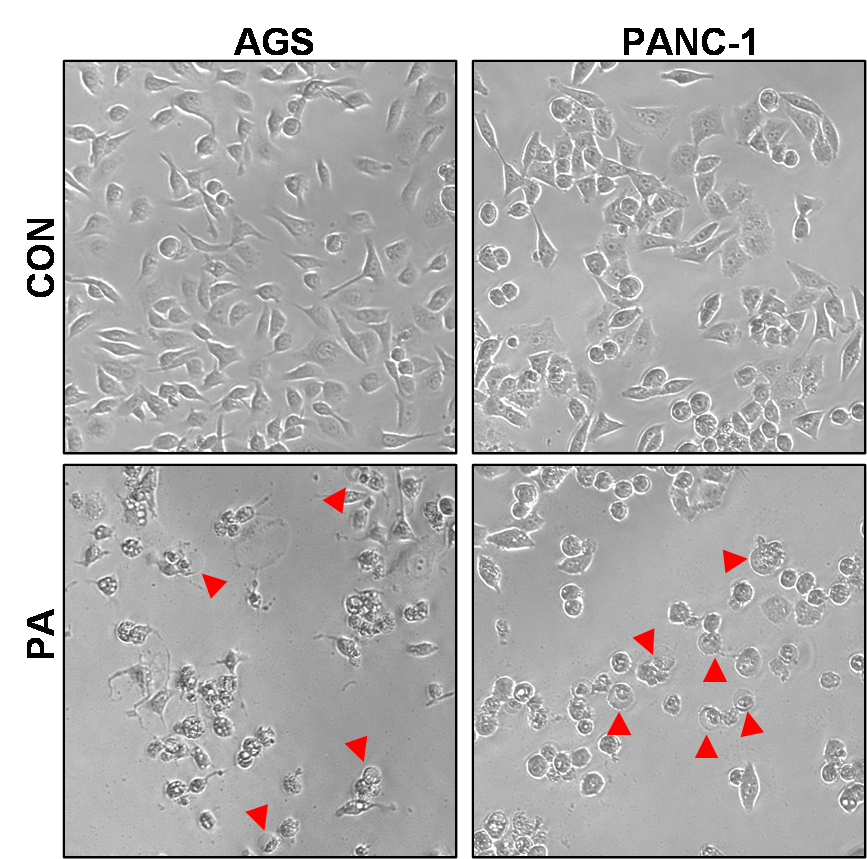
**

**Figure S5 Effects of PA on other human cancer cell lines.** Morphologic alterations of AGS (gastric cancer) and PANC-1 (pancreatic cancer) cells induced by PA (5 μM), the red arrow indicates pyroptosis.

**
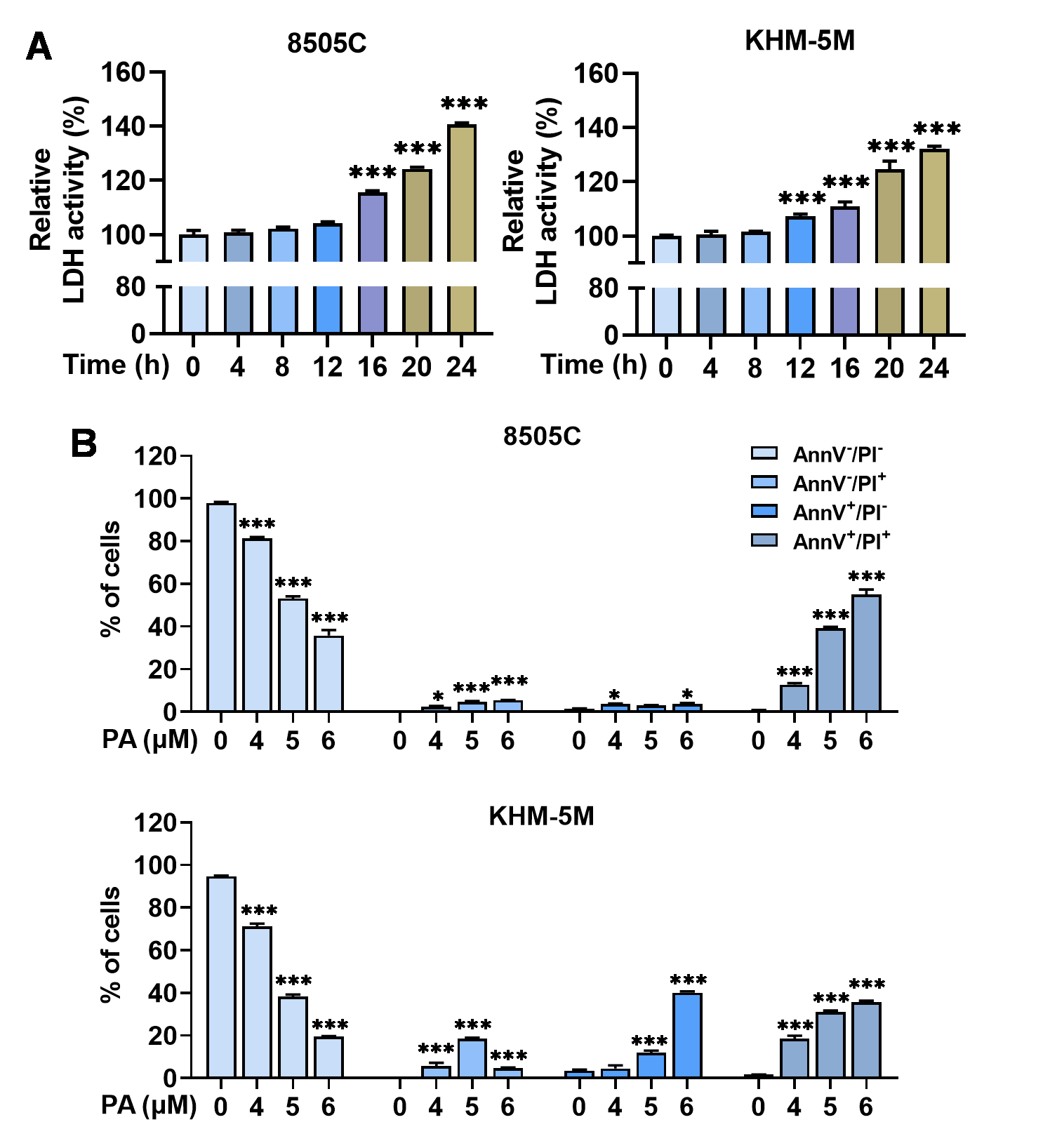
**

**Figure S6 PA induced pyroptosis in ATC cells. (A)** After PA (5 μM) treated for 0，4, 8, 12, 16, 20, 24 h in 8505C and KHM-5M cells, LDH activitiy in culture mediums was detected by the LDH assay kit. **(B)** After 8505C and KHM-5M cells were treated with PA for 24 h, at indicated concentrations (0, 4, 5 and 6 μM), percentage of AnnV^-^/PI^-^, AnnV^-^/PI^+^, AnnV^+^/PI^-^ and AnnV^+^/PI^+^ were indicated. Data are shown as mean ± SD for n = 3 (biological replicates). **p* < 0.05, ****p* < 0.001.

**
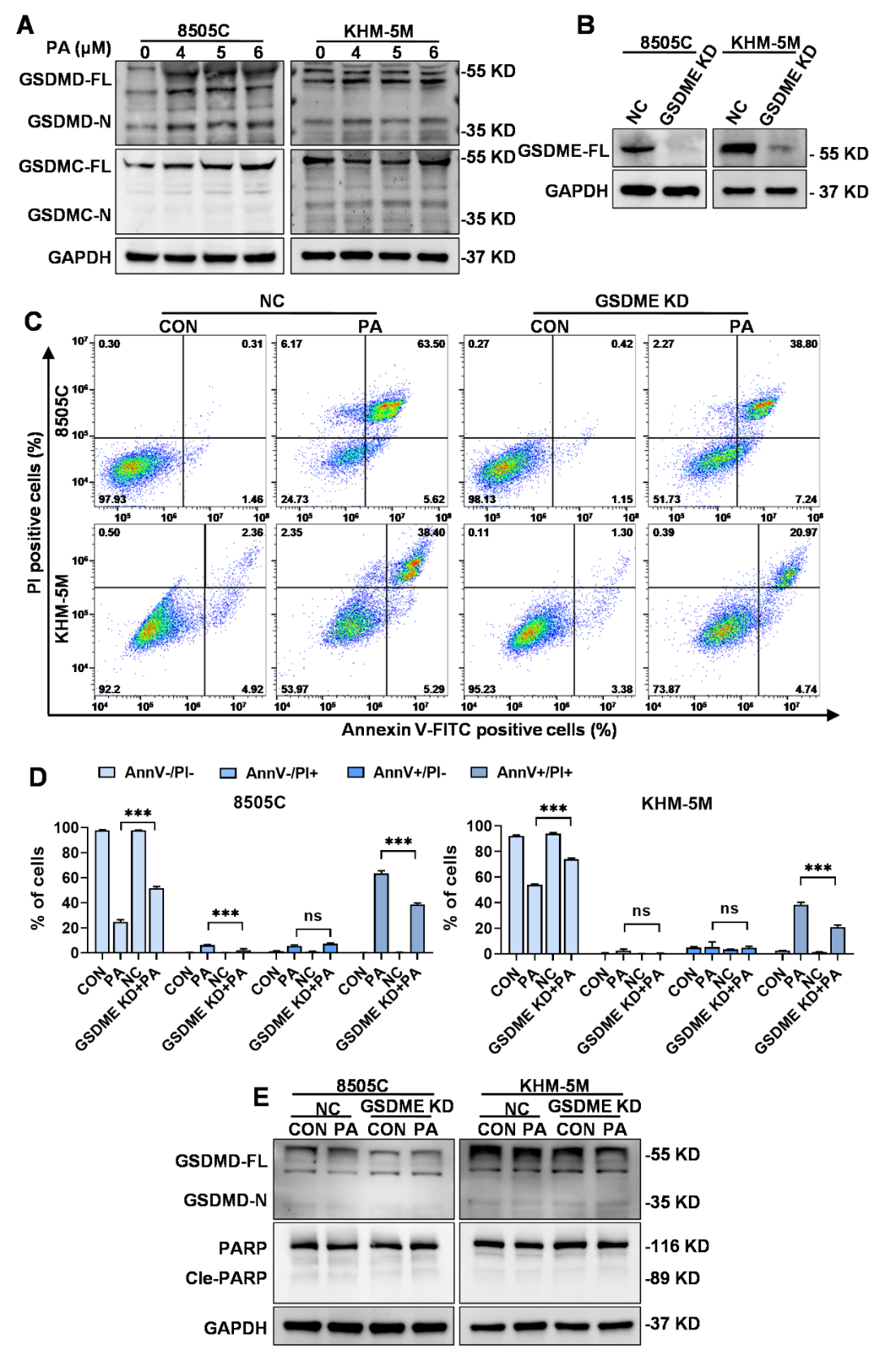
**

**Figure S7 PA elicits GSDME-dependent pyroptosis in ATC cells. (A)** After PA (0, 4, 5 and 6 μM) treatment for 24 h, the GAPDH, full-length GSDMC/D and GSDMC/D-N terminus protein levels were measured by western blot. **(B)** After knockdown GSDME of 8505C and KHM-5M cells, full-length GSDME and GAPDH protein levels were measured by western blot. After knockdown GSDME, 8505C and KHM-5M cells were treated with PA (5μM) for 24 h, **(C)** cell death was assessed by flow cytometry, **(D)** the percentage of AnnV^-^/PI^-^, AnnV^-^/PI^+^, AnnV^+^/PI^-^ and AnnV^+^/PI^+^ were indicated, **(E)** the GAPDH, full-length GSDMD, GSDMD-N terminus, PARP and cleaved-PARP protein levels were measured by western blot. Data are shown as mean ± SD for n = 3 (biological replicates). ****p* < 0.001, ns, *p* ＞ 0.05.

**
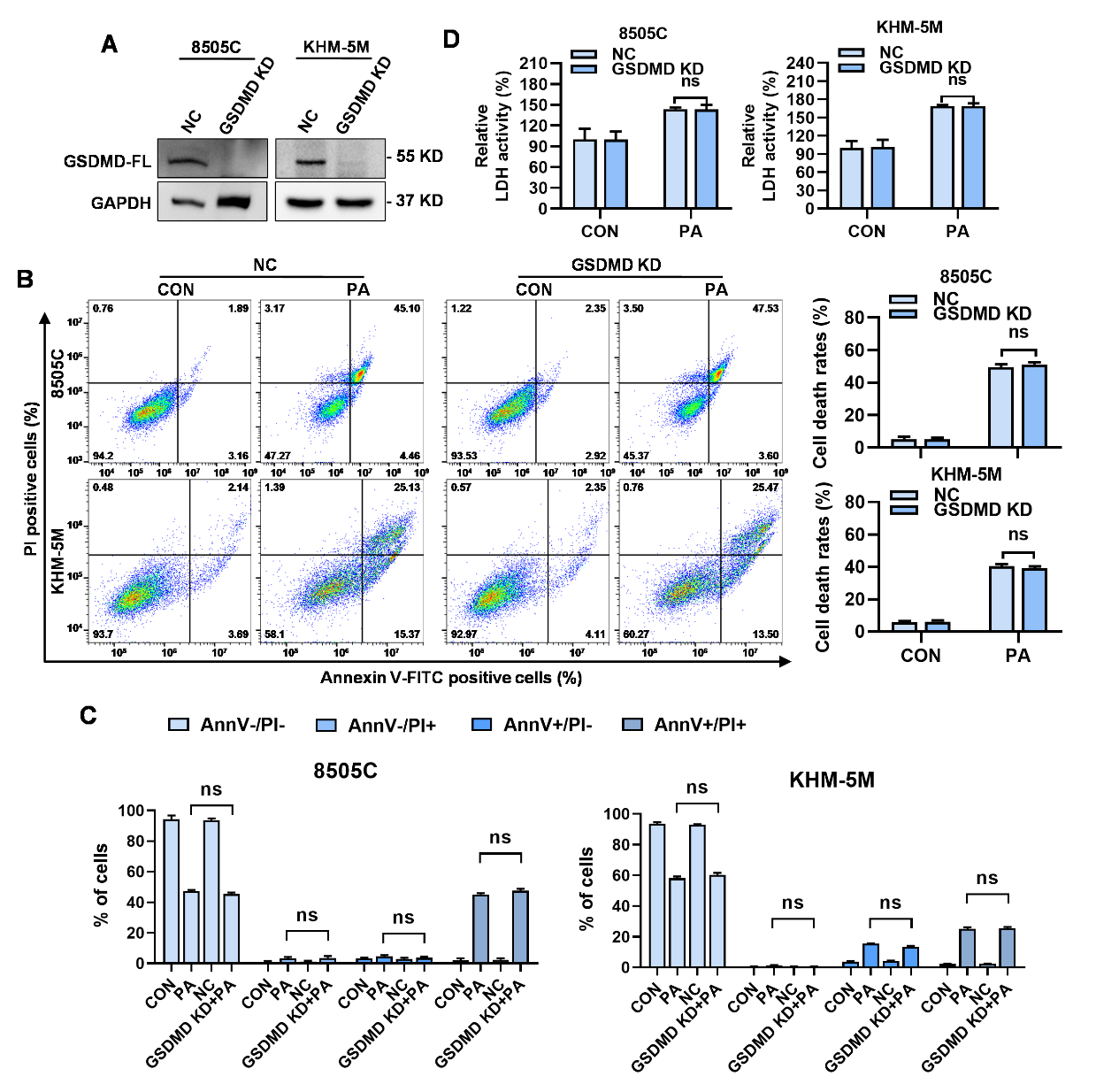
**

**Figure S8** **GSDMD is not involved in PA-induced pyroptosis in ATC cells.** **(A)** After knockdown GSDMD of 8505C and KHM-5M cells, full-length GSDMD and GAPDH protein levels were measured by western blot. After knockdown GSDMD, 8505C and KHM-5M cells were treated with PA (5μM), **(B, C)** cell death was assessed by flow cytometry and the percentage of AnnV^-^/PI^-^, AnnV^-^/PI^+^, AnnV^+^/PI^-^ and AnnV^+^/PI^+^ were presented, **(D)** meanwhile relative LDH activity in culture mediums detected by the LDH assay kit. Data are shown as mean ± SD for n = 3 (biological replicates). ns, *p* ＞ 0.05.

**
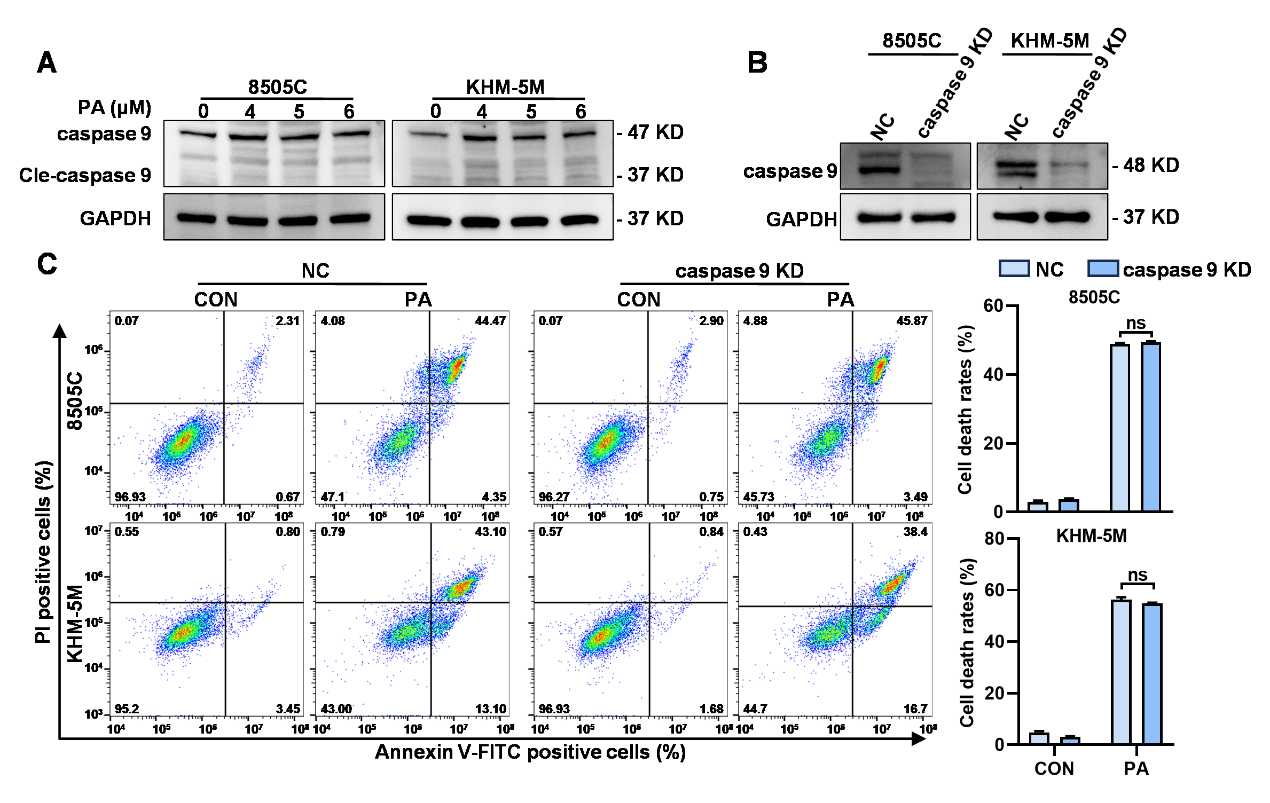
**

**Figure S9 PA induced caspase 9-independent pyroptosis in ATC cells. (A)** After PA (0, 4, 5 and 6 μM) treatment for 24 h, the GAPDH, caspase 9 and cleaved-caspase 9 protein levels were measured by western blot. After knockdown caspase 9 of 8505C and KHM-5M cells, **(B)** caspase 9 and GAPDH protein levels were detected by western blot, **(C)** treated with PA (5 μM) for 24 h, cell death was assessed by flow cytometry. Data are shown as mean ± SD for n = 3 (biological replicates). ns, *p* ＞ 0.05.

**
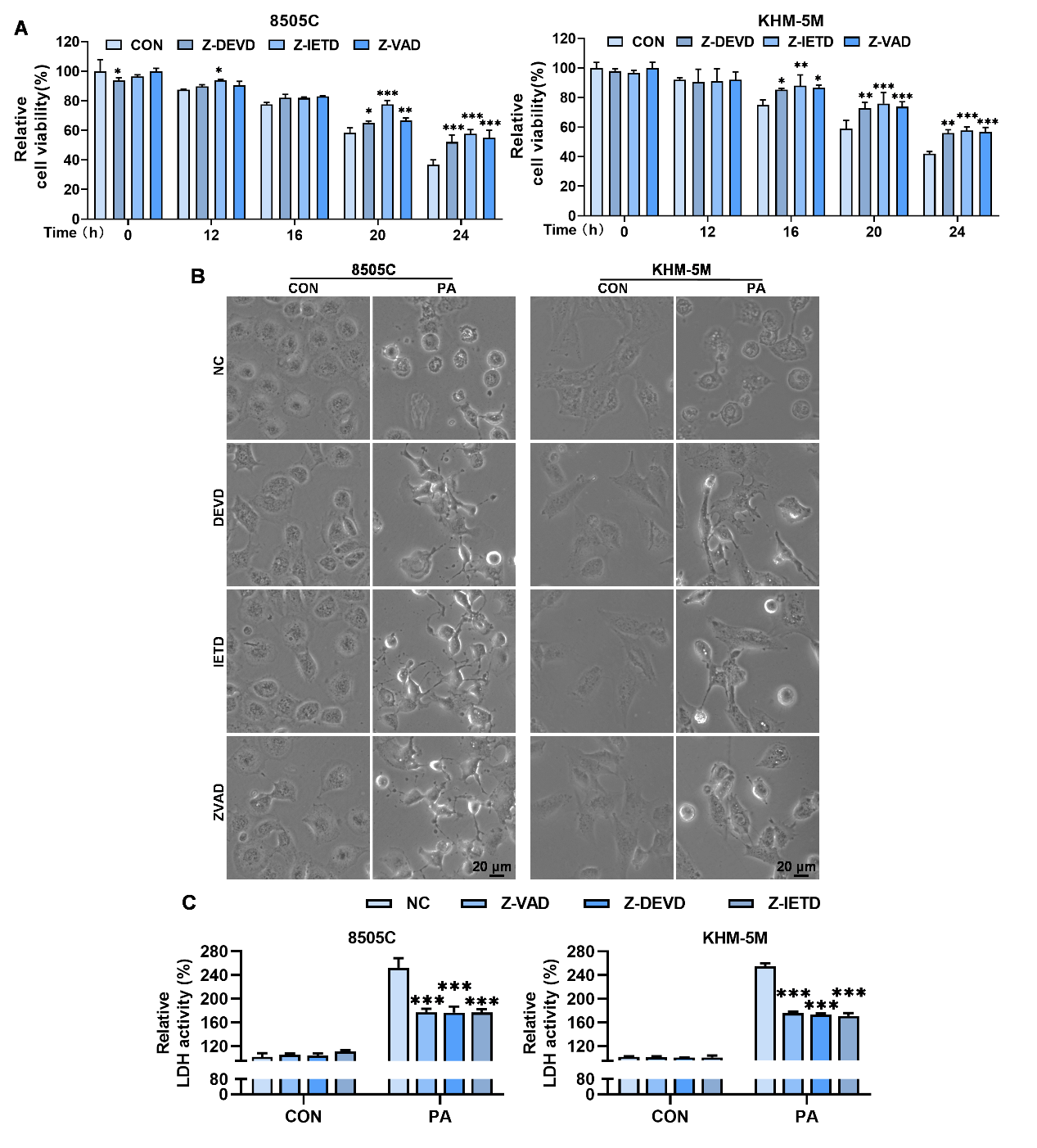
**

**Figure S10 PA induced GSDME-dependent pyroptosis through the caspase 8/3 pathway. (A)** In 8505C and KHM-5M cells, PA (5 μM) is used in combination with Z-VAD (10 μM, pre-treated for 2 h), Z-IETD (10 μM, pre-treated for 2 h) and Z-DEVD (10 μM, pre-treated for 2 h) for 0, 12, 16, 20, 24 h, relative cell viabilities were measured by the CCK-8 assay. **(B)** PA (5 μM) is used in combination with Z-VAD (10 μM, pre-treated for 2 h), Z-IETD (10 μM, pre-treated for 2 h) and Z-DEVD (10 μM, pre-treated for 2 h) for 24 h, morphologies were photographed by a phase contrast microscopy, (scale bar: 20 μm), **(C)** and relative LDH activity in culture mediums detected by the LDH assay kit. Data are shown as mean ± SD for n = 3 (biological replicates). **p* < 0.05, ***p* < 0.01, ****p* < 0.001.

**
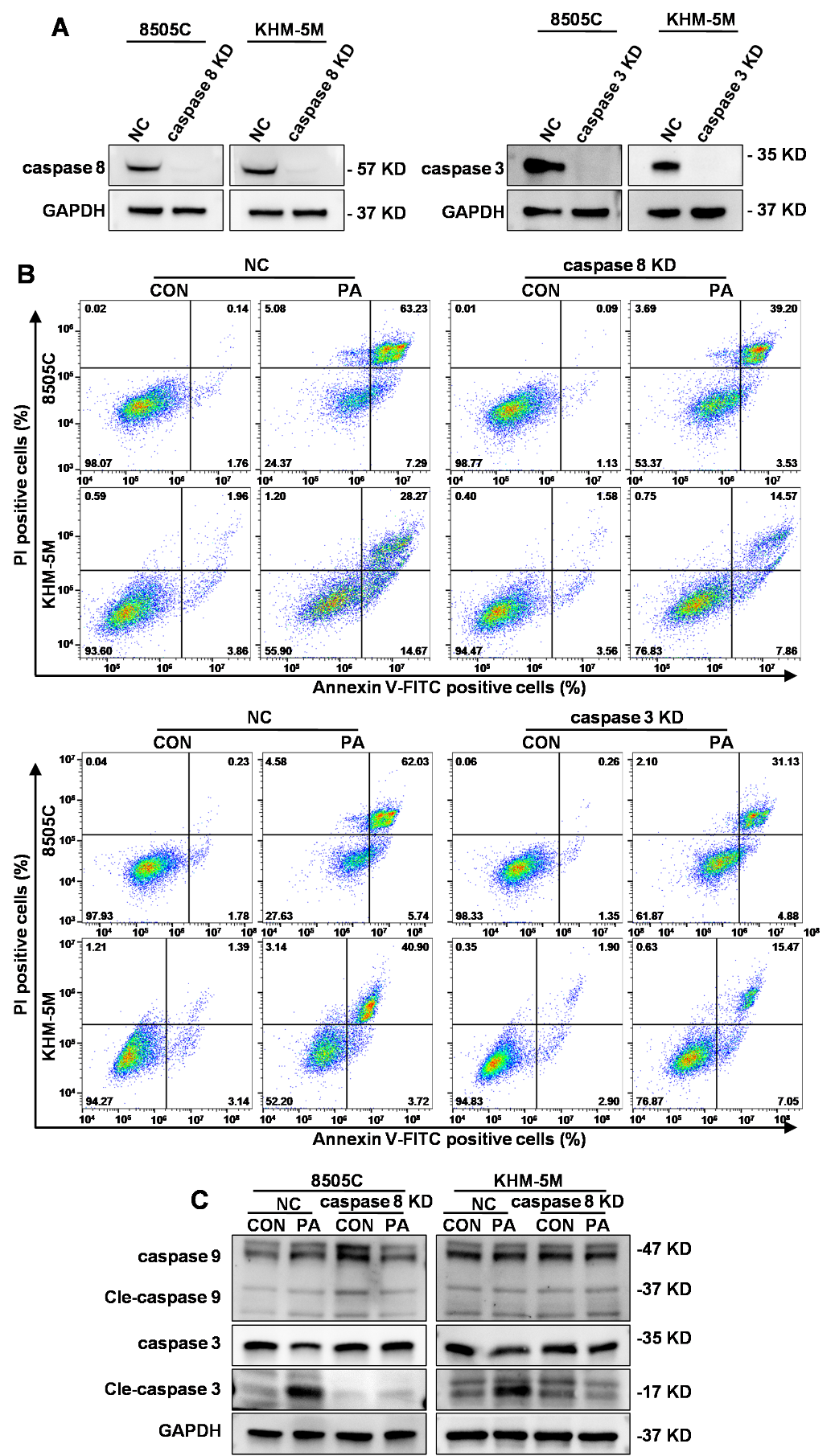
**

**Figure S11 PA induced GSDME-dependent pyroptosis through the caspase 8/3 pathway. (A)** After knockdown caspase 8/3 of 8505C and KHM-5M cells, caspase 8/3 and GAPDH protein levels were detected by western blot. After knockdown caspase 8/3 of 8505C and KHM-5M cells and treated with PA (5 μM) for 24 h, **(B)** cell death was measured by flow cytometry, **(C)** caspase 9/3 and cleaved-caspase 9/3 protein levels were detected by western blot.

**
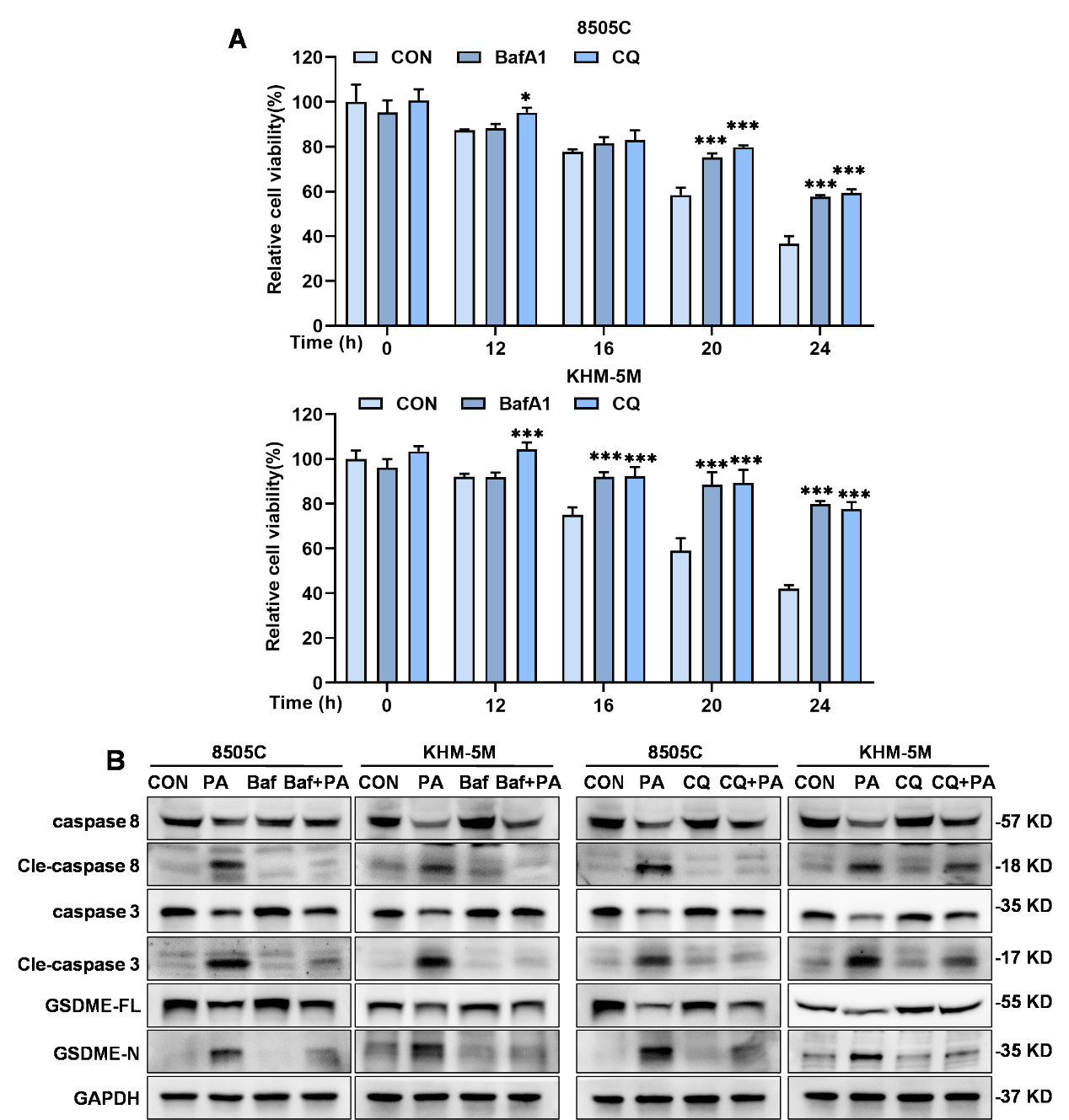
**

**Figure S12 Both CQ and BafA1 block the activation of the caspase 8/3-GSDME pathway in PA-treated-ATC cells. (A)** In 8505C and KHM-5M cells PA (5 μM) is used in combination with BafA1 (100 nM, pre-treated for 2 h) and CQ (10 μM, pre-treated for 2 h) for 0, 12, 16, 20, 24 h, the cell viabilities were measured by the CCK-8 assay. **(B)** In 8505C and KHM-5M cells, PA (5 μM) is used in combination with BafA1 (100 nM, pre-treated for 2 h) and CQ (10 μM, pre-treated for 2 h) for 24 h, and the GAPDH, caspase 8/3, cleaved-caspase 8/3, full-length GSDME, and GSDME-N terminus protein levels were measured by western blot. Data are shown as mean ± SD for n = 3 (biological replicates). **p* < 0.05, ****p* < 0.001.

**
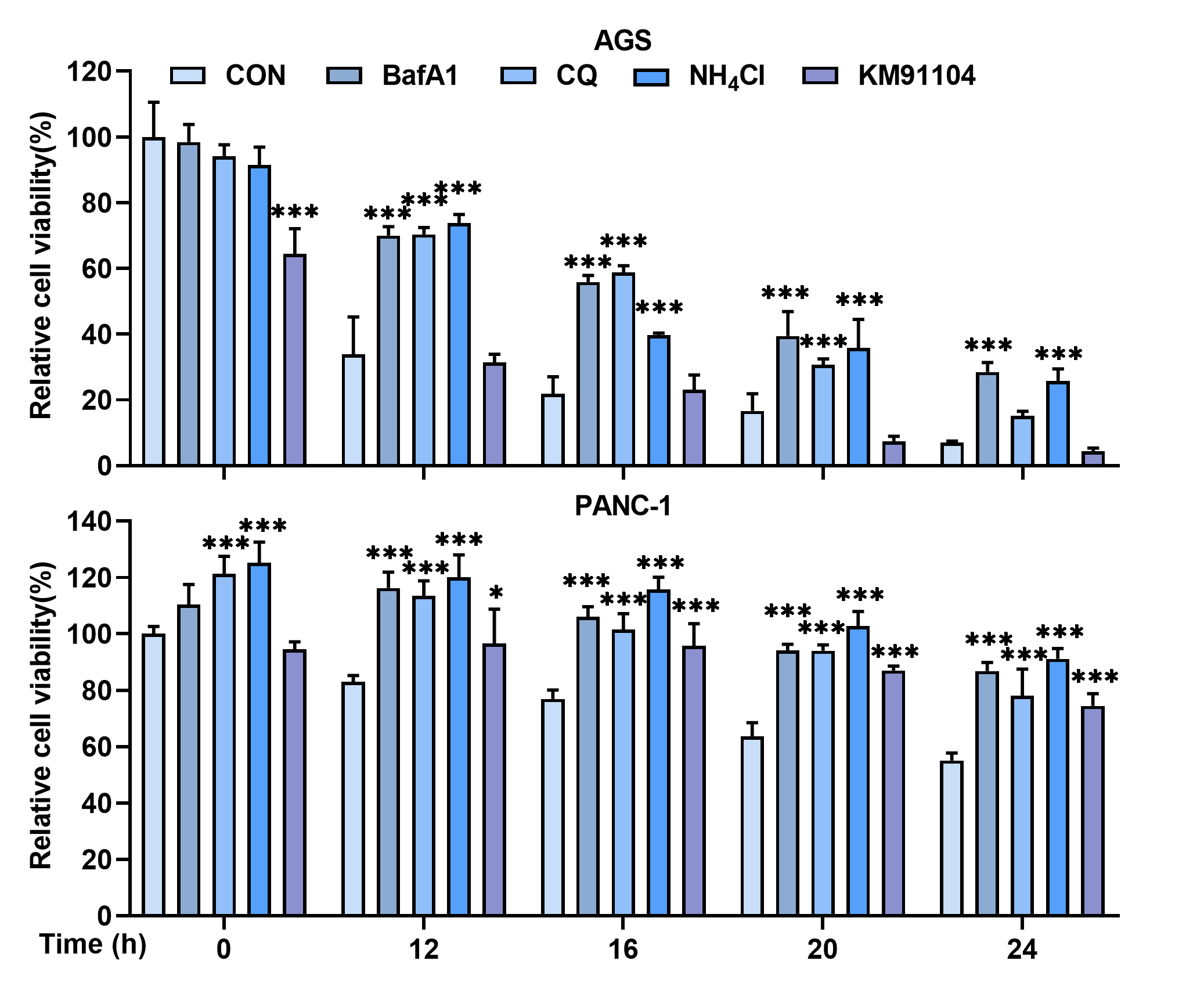
**

**Figure S13 Effects of PA on other human cancer cell lines.** In AGS (gastric cancer) and PANC-1 (pancreatic cancer) cells, PA (5 μM) is used in combination with BafA1 (100 nM, pre-treated for 2 h), CQ (10 μM, pre-treated for 2 h), NH_4_Cl (20 μM, pre-treated for 2 h) and KM91104 (1 nM, pre-treated for 2 h) for 0, 12, 16, 20, 24 h, the cell viabilities were measured by the CCK-8 assay. Data are shown as mean ± SD for n = 3 (biological replicates). **p* < 0.05, ****p* < 0.001.

**
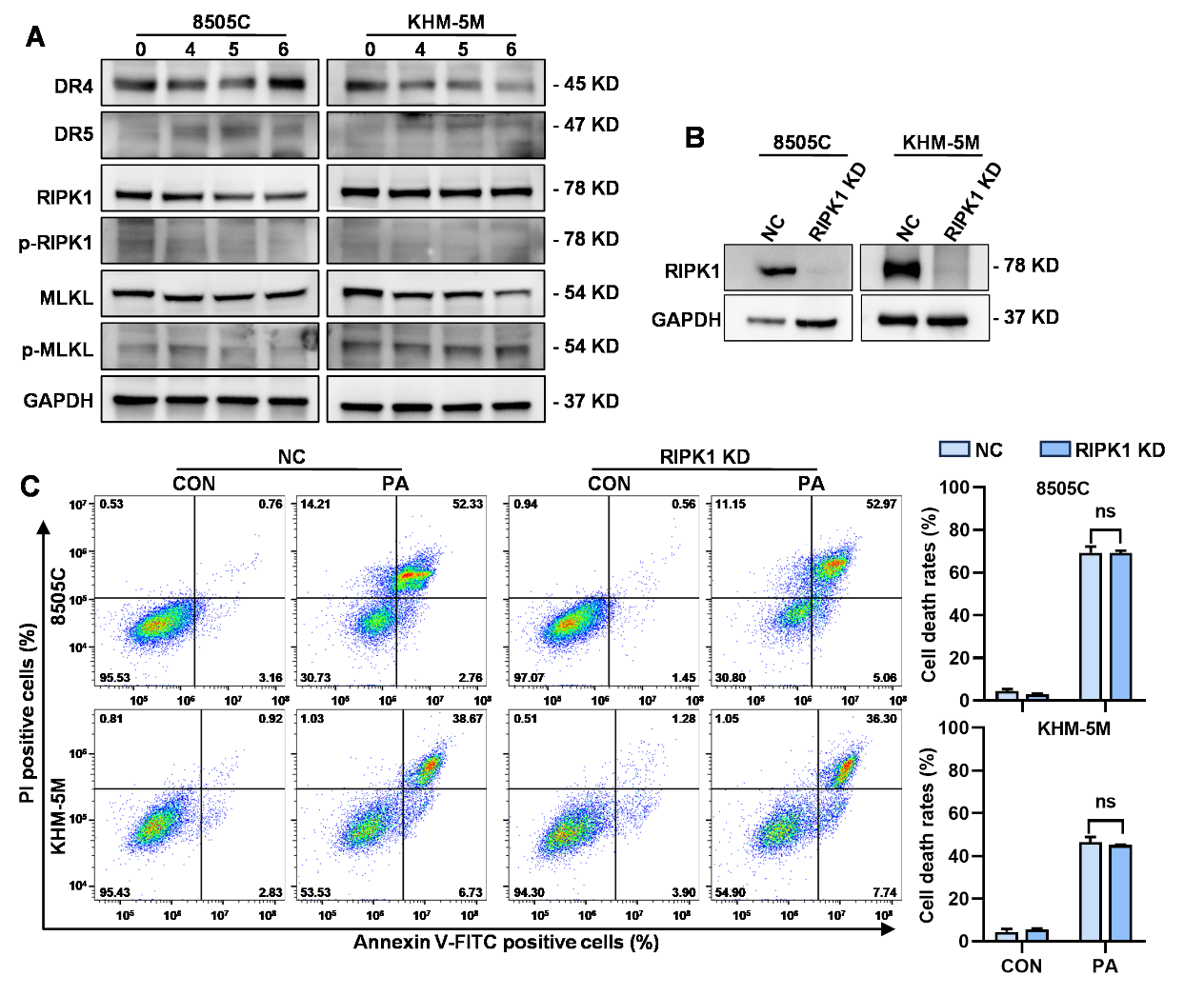
**

**Figure S14 PA induces pyroptosis in ATC cells independent of DR4, DR5 and RIPK1 pathway. (A)** The DR4, DR5, MLKL, p-MLKL, RIPK1, p-RIPK1 and GAPDH protein levels were detected by western blot, after 8505C and KHM-5M cells were treated with PA (0, 4, 5 and 6 μM). **(B)** After knockdown RIPK1 of 8505C and KHM-5M, the RIPK1 and GAPDH protein levels were detected by western blot. **(C)** After knockdown RIPK1 and PA (5 μM) treated of 8505C and KHM-5M, cell death was assessed by flow cytometry. Data are shown as mean ± SD for n = 3 (biological replicates). ns, p ＞ 0.05.

**
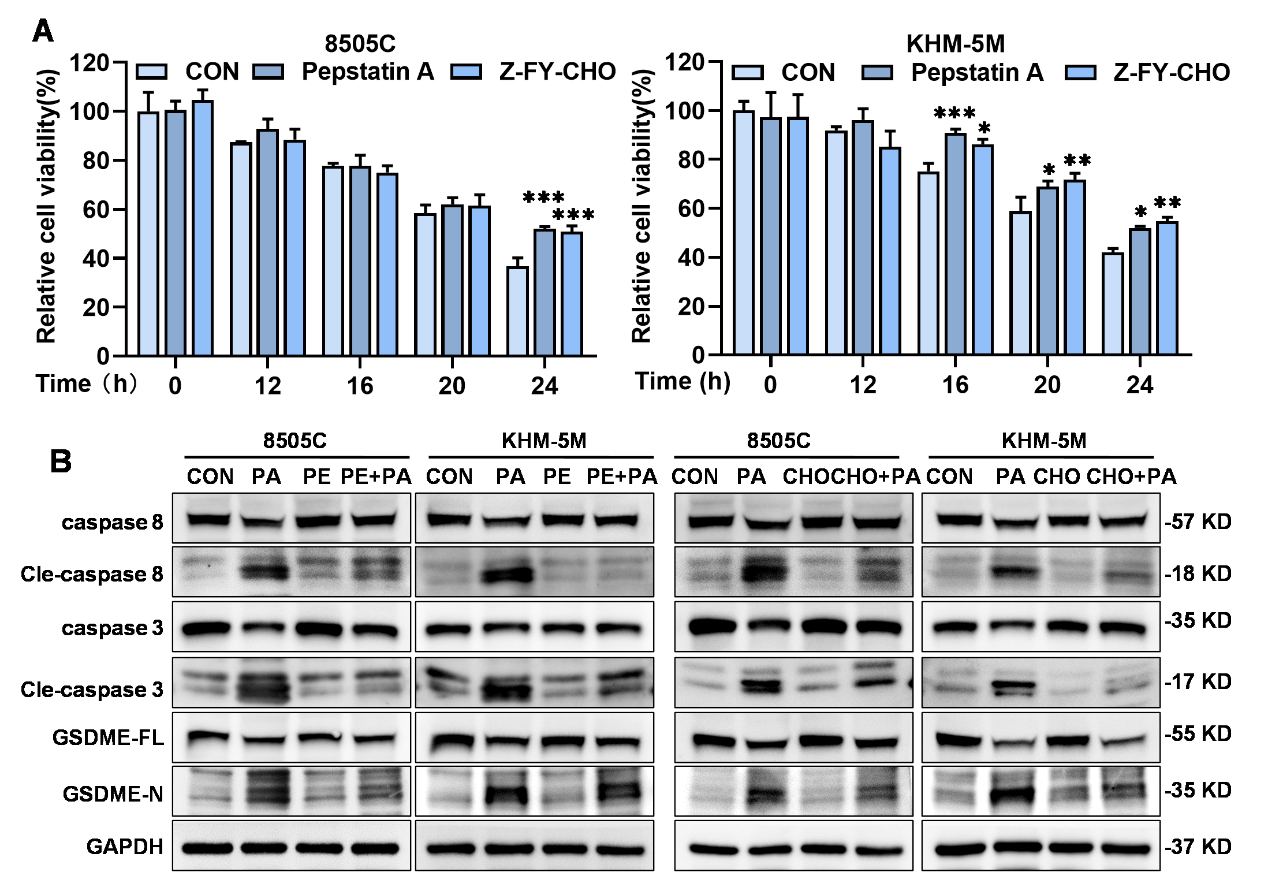
**

**Figure S15 Cathepsins inhibitors reversed the activation of the caspase 8/3-GSDME pathway induced by PA. (A)** In 8505C and KHM-5M cells, PA (5 μM) is used in combination with pepstatin A (10 μM, pre-treated for 2 h) and Z-FY-CHO (20 μM, pre-treated for 2 h) for 0, 12, 16, 20, 24 h, relative cell viabilities were measured by the CCK-8 assay. **(B)** In 8505C and KHM-5M cells, PA (5 μM) is used in combination with pepstatin A (10 μM, pre-treated for 2 h) and Z-FY-CHO (20 μM, pre-treated for 2 h) for 24 h, and the GAPDH, caspase 8/3, cleaved-caspase 8/3, full-length GSDME, and GSDME-N terminus protein levels were measured by western blot, PE: pepstatin A, CHO: Z-FY-CHO. Data are shown as mean ± SD for n = 3 (biological replicates). **p* < 0.05, ***p* < 0.01, ****p* < 0.001.

**
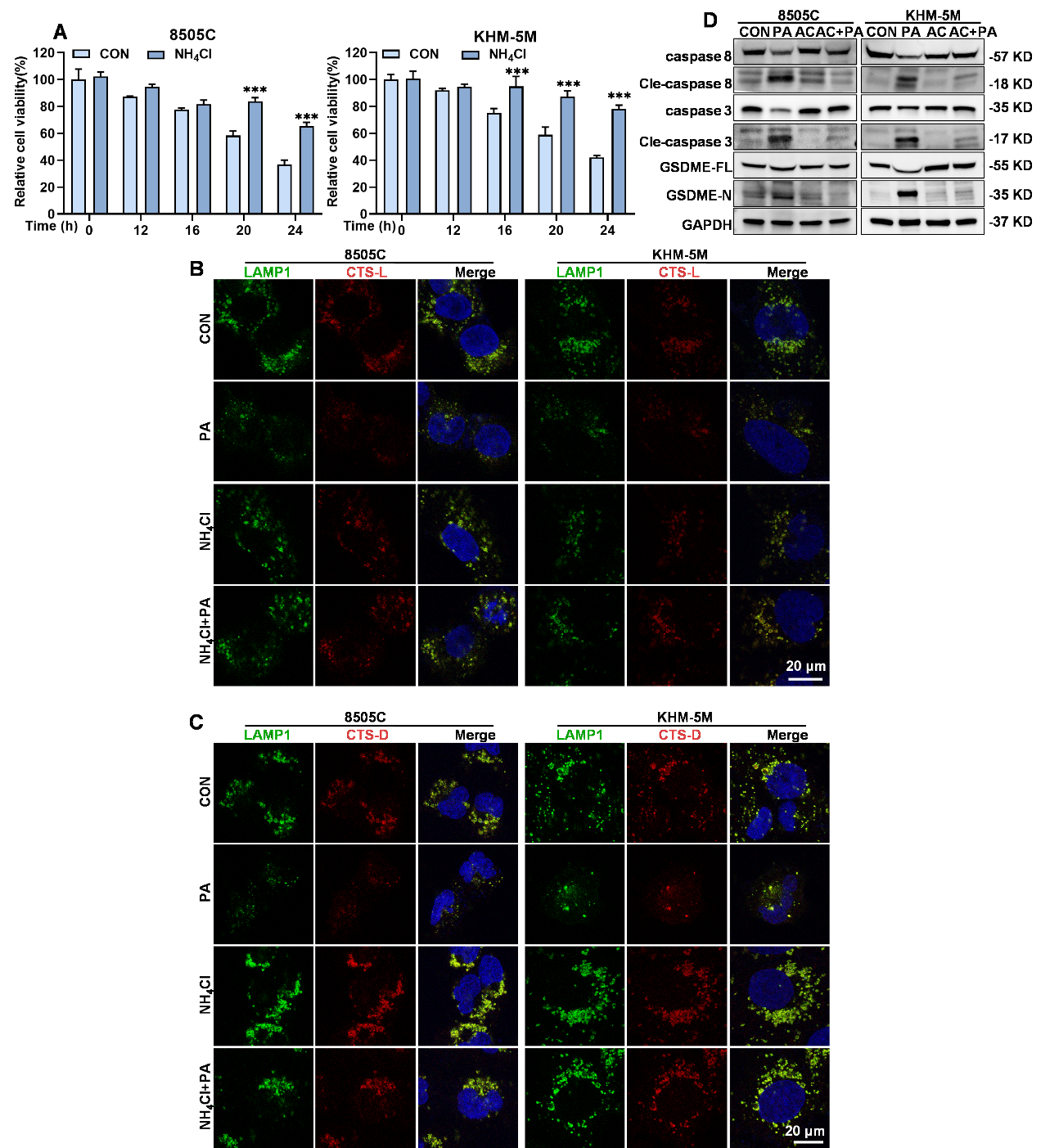
**

**Figure S16 NH_4_Cl blocks the activation of the caspase 8/3-GSDME pathway in PA-treated-ATC cells. (A)** In 8505C and KHM-5M cells, cell viabilities following treatment with PA (5 μM) in the presence or absence of NH_4_Cl (20 μM, pre-treated for 2 h) for 0, 12, 16, 20, 24 h determined by the CCK-8 assay kit. **(B, C)** In 8505C and KHM-5M cells, PA (5 μM) is used in combination with NH_4_Cl (20 μM, pre-treated for 2 h) for 12 h, the immunofluorescence analysis was performed with anti-LAMP1 antibody (green), anti-CTS-L antibody (red), anti-CTS-D antibody (red), and DAPI (blue), the morphologies were determined by confocal microscopy, (scale bar: 20 μm). **(D)** In 8505C and KHM-5M cells, PA (5 μM) is used in combination with NH_4_Cl (20 μM, pre-treated for 2 h) for 24 h, and the GAPDH, caspase 8/3, cleaved-caspase 8/3, full-length GSDME, and GSDME-N terminus protein levels were measured by western blot, AC: NH_4_Cl (Ammonium chloride). Data are shown as mean ± SD for n = 3 (biological replicates). ****p* < 0.001.

**
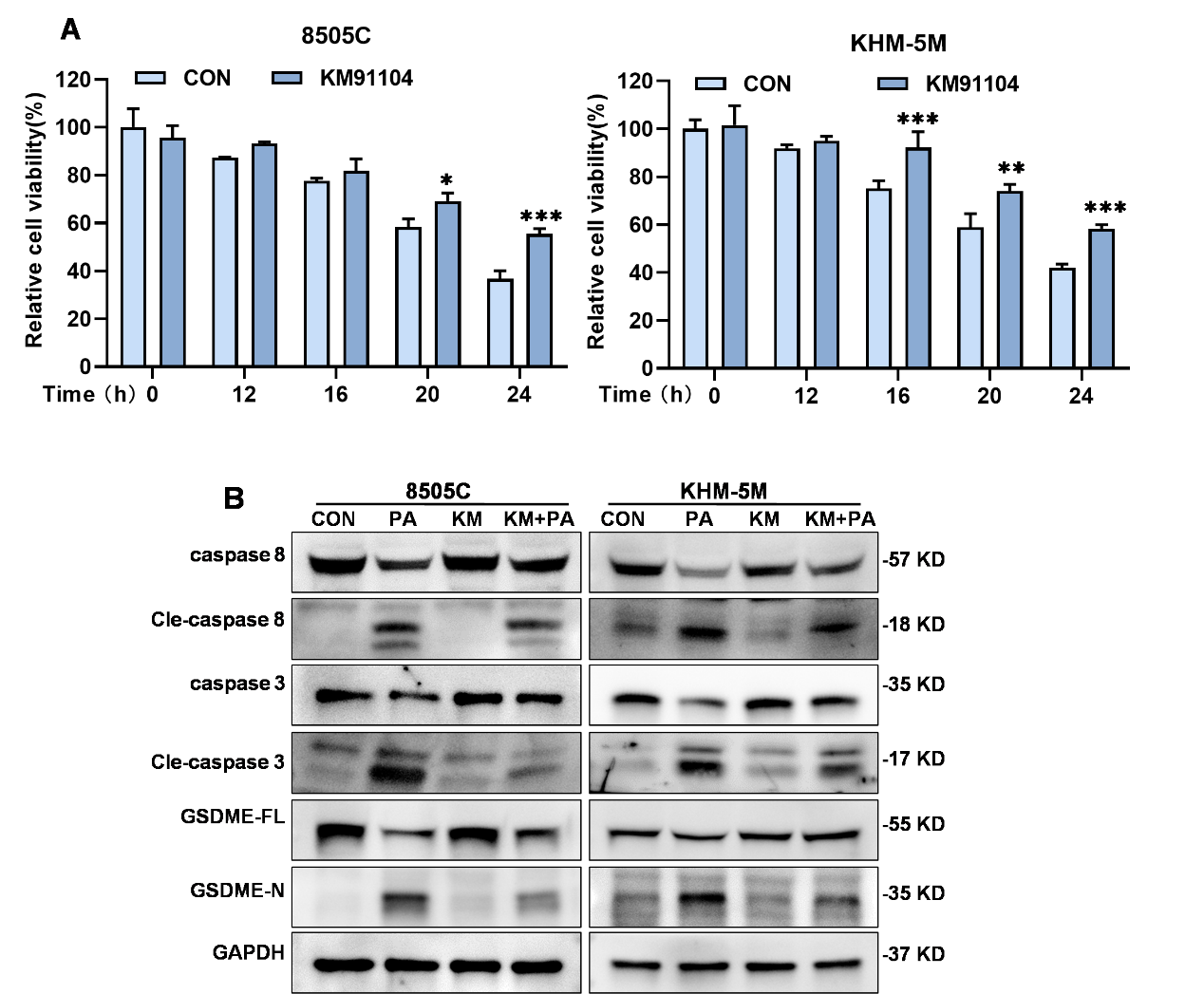
**

**Figure S17 KM91104 blocks the activation of the caspase 8/3-GSDME pathway in PA-treated-ATC cells. (A)** In 8505C and KHM-5M cells, cell viabilities following treatment with PA (5 μM) in the presence or absence of KM91104 (1 nM, pre-treated for 2 h) for 0, 12, 16, 20, 24 h determined by the CCK-8 assay kit. **(B)** In 8505C and KHM-5M cells, PA (5 μM) is used in combination with KM91104 (1 nM, pre-treated for 2 h) for 24 h, and the GAPDH, caspase 8/3, cleaved-caspase 8/3, full-length GSDME, and GSDME-N terminus protein levels were measured by western blot, KM: KM91104. Data are shown as mean ± SD for n = 3 (biological replicates). **p* < 0.05, ***p* < 0.01, ****p* < 0.001.

**
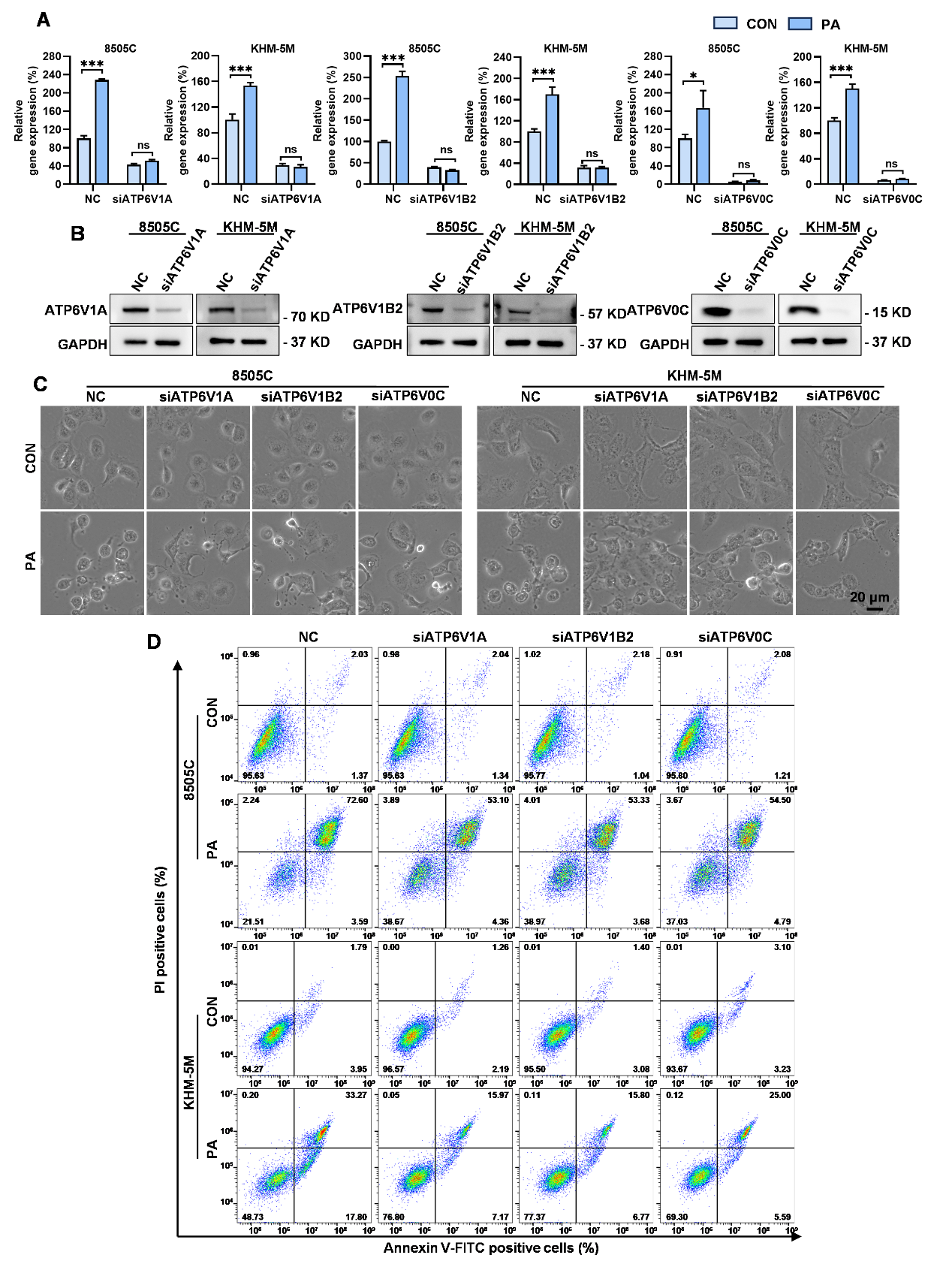
**

**Figure S18 V-ATPase overactivation induced pyroptosis in PA-treated ATC cells. (A)** The 8505C and KHM-5M cells transfected with ATP6V1A, ATP6V1B2, ATP6V0C si-RNA, were treated with 5 μM PA for 24 h, the relative genes expression was calculated. **(B)** The 8505C and KHM-5M cells were transfected with ATP6V1A, ATP6V1B2, ATP6V0C si-RNA, and the GAPDH, ATP6V1A, ATP6V1B2 and ATP6V0C protein levels were measured by western blot, respectively. **(C)** The 8505C and KHM-5M cells transfected with ATP6V1A, ATP6V1B2, ATP6V0C si-RNA, were treated with 5 μM PA for 24 h, the morphologies were determined by microscopy, (scale bar: 20 μm), and **(D)** cell deaths were determined by flow cytometry. Data are shown as mean ± SD for n = 3 (biological replicates). **p* < 0.05, ****p* < 0.001, ns, *p* ＞ 0.05.

**
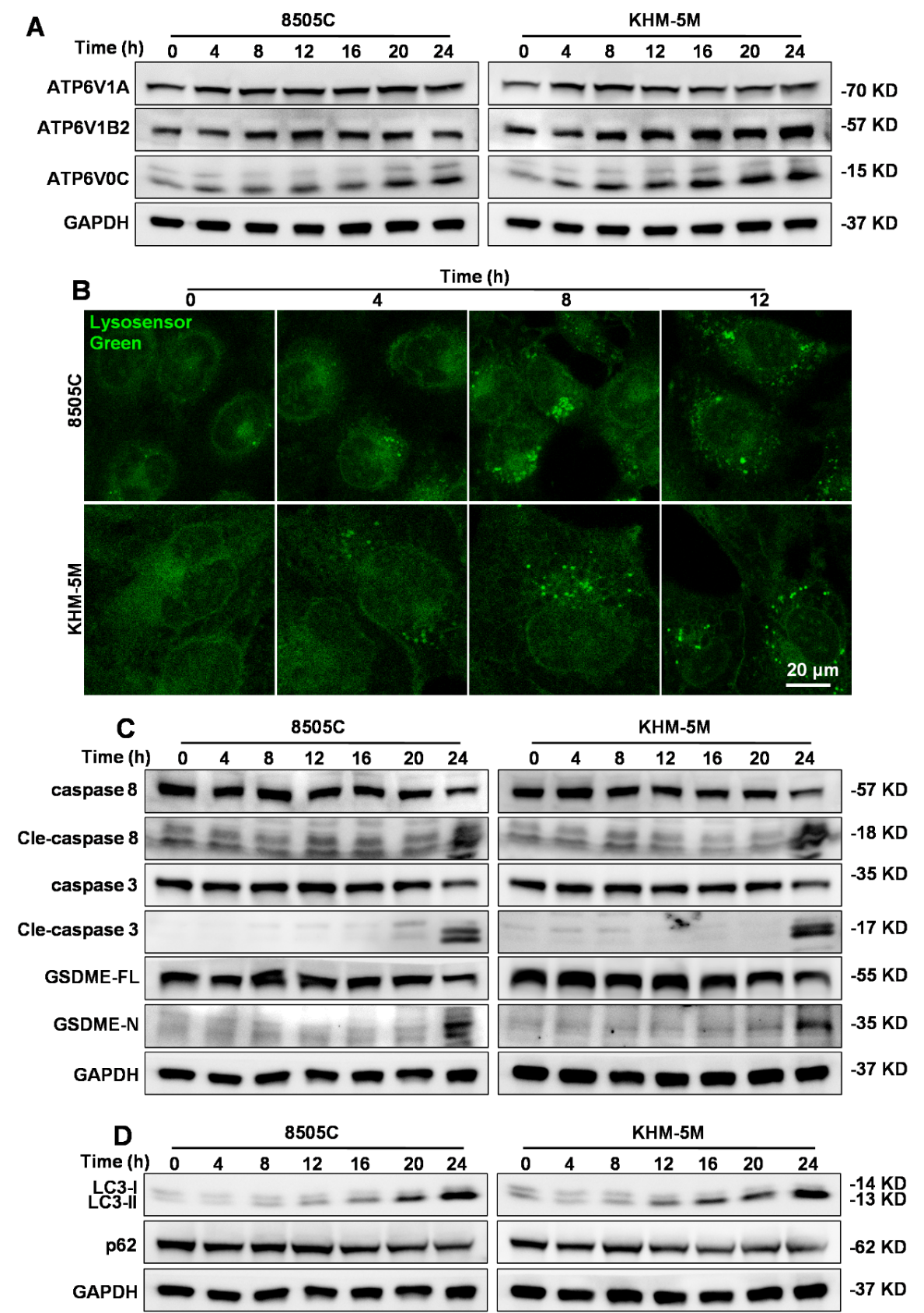
**

**Figure S19 PA induced the activation of the caspase 8/3-GSDME pathway in ATC cells by time-dependent manner. (A)** After PA (5 μM) treated for 0, 4, 8, 12, 16, 20, 24 h in 8505C and KHM-5M cells, ATP6V1A, ATP6V1B2, ATP6V0C and GAPDH protein levels were detected by western blot. **(B)** After treated with PA (5 μM) for 0, 4, 8, 12 h of 8505C and KHM-5M cells, stained by Lysosensor Green DND-189, the morphologies were determined by confocal microscopy (Scale bar: 20 μm). **(C, D)** After PA (5 μM) treatment for 0, 4, 8, 12, 16, 20, 24 h in 8505C and KHM-5M cells, the GAPDH, caspase 8/3, cleaved-caspase 8/3, full-length GSDME, GSDME-N terminus, p62 and LC3-I/II protein levels were measured by western blot.

**
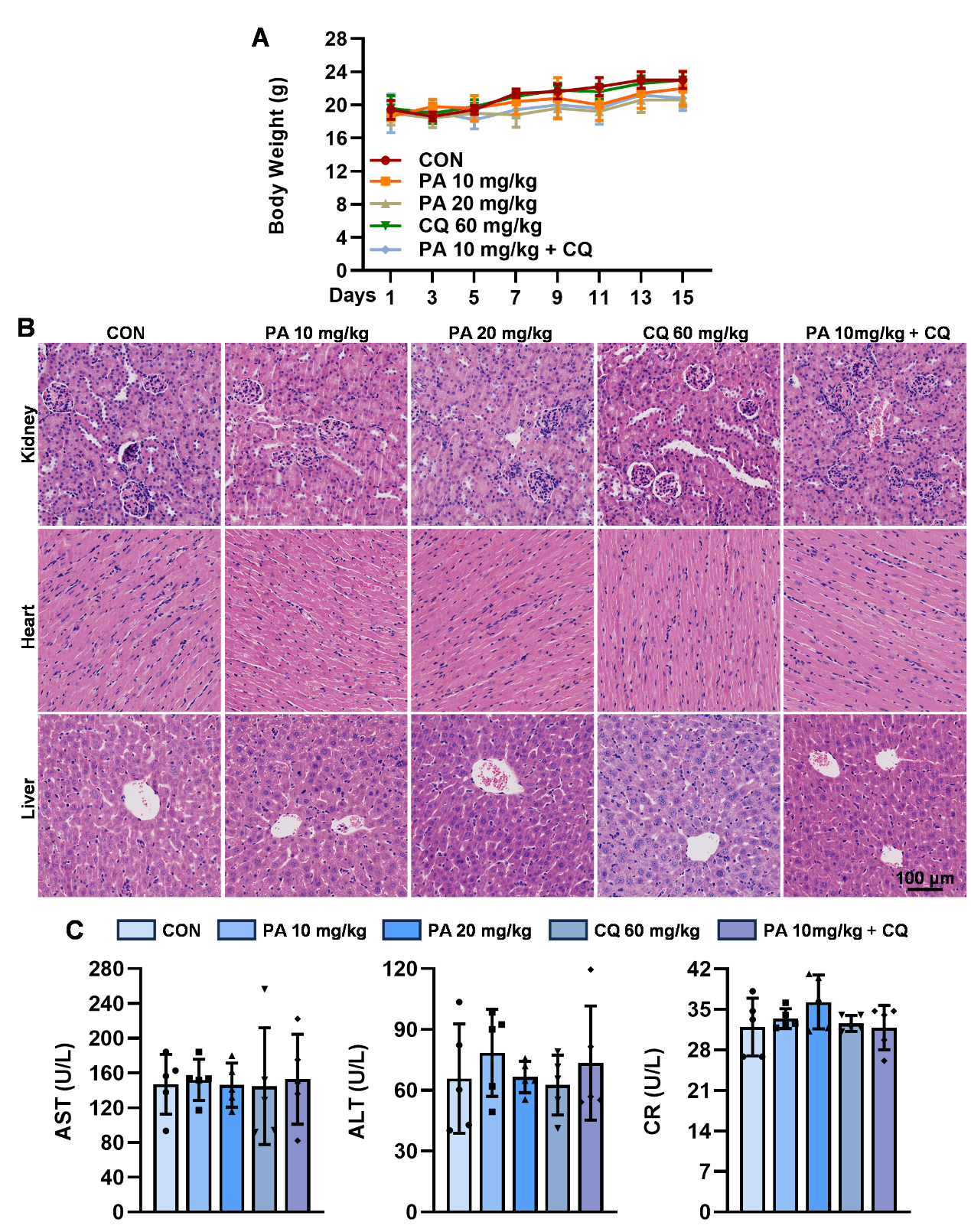
**

**Fig. S20 PA exhibits high safety *in vivo*. (A)** Body weight of nude mice for 15 days. **(B)** Histological analysis of kidney, heart, and liver, (scale bar: 100 μm). **(C)** AST, ALT, CR expression in mice. Data are shown as mean ± SD for n = 5 (biological replicates).
